# Cross-Feeding and Enzymatic Catabolism for Mannan-Oligosaccharide Utilization by the Butyrate-Producing Gut Bacterium *Roseburia hominis* A2-183

**DOI:** 10.3390/microorganisms10122496

**Published:** 2022-12-16

**Authors:** Abhishek Bhattacharya, Lovisa Majtorp, Simon Birgersson, Mathias Wiemann, Krishnan Sreenivas, Phebe Verbrugghe, Olivier Van Aken, Ed W. J. Van Niel, Henrik Stålbrand

**Affiliations:** 1 Division of Biochemistry and Structural Biology, Department of Chemistry, Lund University, Naturvetarvägen 14, 221 00 Lund, Sweden; lovisa.majtorp@biochemistry.lu.se (L.M.); simon.birgersson@biochemistry.lu.se (S.B.); mathias.wiemann@biochemistry.lu.se (M.W.); 2 Applied Microbiology, Department of Chemistry, Lund University, Naturvetarvägen 14, 221 00 Lund, Sweden; krishnan.sreenivas@tmb.lth.se (K.S.); ed.van_niel@tmb.lth.se (E.W.J.V.N.); 3 Department of Food Technology, Engineering and Nutrition, Lund University, Naturvetarvägen 14, 221 00 Lund, Sweden; vphebe4@yahoo.com; 4 Department of Biology, Lund University, Sölvegatan 35, 223 62 Lund, Sweden; olivier.van_aken@biol.lu.se

**Keywords:** β-mannan-oligosaccharides, *Roseburia hominis*, *Bifidobacterium adolescentis*, cocultivation, cross-feeding, butyrate production, differential gene expression, β-1,4 mannan-oligosaccharide phosphorylase, α-galactosidase, exo-oligomannosidase

## Abstract

β-Mannan is abundant in the human diet and in hemicellulose derived from softwood. Linear or galactose-substituted β-mannan-oligosaccharides (MOS/GMOSs) derived from β-mannan are considered emerging prebiotics that could stimulate health-associated gut microbiota. However, the underlying mechanisms are not yet resolved. Therefore, this study investigated the cross-feeding and metabolic interactions between *Bifidobacterium adolescentis* ATCC 15703, an acetate producer, and *Roseburia hominis* A2-183 DSMZ 16839, a butyrate producer, during utilization of MOS/GMOSs. Cocultivation studies suggest that both strains coexist due to differential MOS/GMOS utilization, along with the cross-feeding of acetate from *B. adolescentis* E194a to *R. hominis* A2-183. The data suggest that *R. hominis* A2-183 efficiently utilizes MOS/GMOS in mono- and cocultivation. Notably, we observed the transcriptional upregulation of certain genes within a dedicated MOS/GMOS utilization locus (*Rh*MosUL), and an exo-oligomannosidase (*Rh*Man113A) gene located distally in the *R. hominis* A2-183 genome. Significantly, biochemical analysis of β-1,4 mannan-oligosaccharide phosphorylase (*Rh*MOP130A), α-galactosidase (*Rh*Gal36A), and exo-oligomannosidase (*Rh*Man113A) suggested their potential synergistic role in the initial utilization of MOS/GMOSs. Thus, our results enhance the understanding of MOS/GMOS utilization by potential health-promoting human gut microbiota and highlight the role of cross-feeding and metabolic interactions between two secondary mannan degraders inhabiting the same ecological niche in the gut.

## 1. Introduction

The human gastrointestinal tract contains a complex microbial community known as the human gut microbiota (HGM) [1,2]. The metabolic capabilities of the HGM assist the human gut to utilize recalcitrant complex nutrients [3,4]. The metabolic activity of the HGM is not only associated with health benefits but also with disease [2,5]. A decrease in the relative abundance of some gut bacteria, e.g., *Bifidobacteria* spp. or *Roseburia* spp., has been associated with health-related problems [5,6]. In the context of human health and physiology, the role of short-chain fatty acids (SCFAs), such as acetate and butyrate, generated during the fermentation of complex glycans by the HGM has gained much attention [7,8]. In particular, the role of butyrate as an important energy source for colonocytes has been extensively studied [5]. Furthermore, other beneficial aspects of butyrate-producing *Roseburia* have also contributed to an increased interest in them [2,5,7]. An interesting possibility could be the health-beneficial manipulation of the HGM composition through dietary carbohydrates such as prebiotic oligosaccharides [9,10,11].

β-Mannans are complex glycans naturally occurring as dietary fibers and are also extensively used as thickeners in the food and feed industry [12,13]. β-Mannans such as galactomannan carry α-galactosyl side groups [12]. Even though β-mannan metabolism has been identified as one of several important pathways for glycan utilization in the human gut microbiota [14], limited studies have focused on understanding the mechanisms of β-mannan or (galacto)-β-mannan-oligosaccharides (MOS/GMOS) utilization by the HGM [15,16,17,18,19,20]. Glycan-processing proteins are often organized into multigene clusters which encode glycan-binding proteins, carbohydrate-active enzymes, and transporters that work together to target a particular glycan [21,22]. Notably, such clusters are prevalent in Gram-negative genus *Bacteroides* and are termed polysaccharide utilization loci (PULs), characterized by outer membrane-bound solute-binding proteins (SBPs), Ton-B dependent transporters, and often endo-acting glycoside hydrolases [1]. In contrast, certain Gram-positive members of the HGM, such as Firmicutes (*Roseburia, Clostridia*) and Actinobacteria (*Bifidobacteria*), harbor glycan-processing proteins in specific utilization loci often characterized by distinct ATP-binding cassette (ABC) transporters that moderate the capturing of oligosaccharides by SBPs located extracellularly [22]. Significantly, several impactful studies on inulin and xylan utilization by gut bacteria belonging to Firmicutes and Actinobacteria harboring specific glycan utilization loci have been published [21,23]. However, very few studies on β-mannan and/or MOS/GMOS utilization loci in *Roseburia* spp. [17] and *Bifidobacterium* spp. [24] have been reported.

The interplay within the diverse gut microbiota allows for the breakdown of complex glycans by potential primary degraders such as members of *Bacteroides*, thereby making recalcitrant glycans available to secondary degraders which may lead to cooperation or competition among bacteria in shared nutrient niches within the human gut [22,25]. Notably, some primary β-mannan degraders, such as *Roseburia intestinalis* [17] and *Bifidobacterium animalis* subsp. *lactis* BI-04 [24], have been shown to compete with *Bacteroides ovatus*, another primary mannan degrader [17,18,24]. Interestingly, cooperative interaction was observed between a secondary degrader (MOS/GMOS consumer), *Faecalibacterium prausnitzii,* and the primary degraders *R. intestinalis* L1-82 or *Ba. ovatus* [15]. Recently, we demonstrated that *Roseburia hominis* A2-183 DSMZ 16839 (hereafter *R. hominis*) and *Bifidobacterium adolescentis* E194a ATCC 15703 (hereafter *B. adolescentis*) are secondary degraders (MOS/GMOS consumers) [16], and both the species have been associated with health-promoting effects on the host [26,27]. Some investigations have reported cross-feeding and metabolic interactions, using acetate producers (*Bifidobacterium* spp.) and butyrate producers (*Eubacterium* spp. or *Faecalibacterium* spp.) during coculturing on arabinoxylo-oligosaccharides (AXOSs), starch, or fructo-oligosaccharides (FOSs) [28,29]. However, little attention has been devoted to such cross-feeding during the utilization of MOS/GMOS, which is investigated in this study. Thus, we have chosen two strains that, as secondary mannan degraders, can utilize MOS/GMOS [16], namely the acetate-producing *B. adolescentis* and the acetate-consuming and butyrate-producing *R. hominis*, who are suggested to share the same nutrient niche in the human colon [30]. The mechanistic understanding of the MOS/GMOS utilization by *R. hominis* and *B. adolescentis* would not only assist in strategies for HGM manipulation and contribute to new perspectives regarding glycan-dependent metabolic interactions but would also help us to identify unexplored carbohydrate-active enzymes (CAZymes) from gut bacteria.

In this study, we investigated whether cross-feeding and metabolic interactions occur between *B. adolescentis* and *R. hominis* during the fermentation of MOS/GMOS or glucose, assessing whether competitive or cooperative behavior occurs during cocultivation. Moreover, we investigated the mechanistic role of a putative *R. hominis* (galacto)-β-mannan-oligosaccharides (MOS/GMOSs) utilization locus (*Rh*MosUL, locus tag: RHOM_RS11115-11180), which was brought to attention by previous in silico sequence analyses of *R. hominis* genome [16,17]. Thus, herein we conducted a transcript analysis of certain *Rh*MosUL putative genes. Furthermore, we characterized selected proteins encoded by *Rh*MosUL, including a β-mannoside phosphorylase (*Rh*MOP130A, RHOM_RS11135) and an α-galactosidase (*Rh*Gal36A, RHOM_RS11140), which were upregulated during the growth of *R. hominis* on MOS/GMOS, in addition to a distally expressed β-mannoside hydrolase (*Rh*GH113A, RHOM_RS14610). Together, these data allowed us to throw light on the uptake and initial hydrolysis of MOS/GMOS by *R. hominis* and understand aspects of the interplay between *R. hominis* and *B. adolescentis* under in vitro conditions.

## 2. Materials and Methods

### 2.1. Bioinformatic Analysis

#### 2.1.1. Bioinformatic Prediction of *Roseburia hominis* A2-183 Gene Functions

An amino acid sequence similarity analysis was conducted where the RefSeq annotated protein sequences listed in Supplementary Table S1 from the genome of *R. hominis* A2-183 (BioProject accession number PRJNA33399) were subjected to BLASTp searches against *Roseburia intestinalis* L1-82 strain (BioProject Accession PRJNA30005) [17] (Supplementary Table S1). The BLASTp (BLOSUM 62-matrix) suite of the National Center for Biotechnology Information (NCBI) was used at the default threshold e-value of 1 × e^−5^. The functional annotation for the *R. hominis* target proteins was predicted based on the high sequence similarity to the homologs in *R. intestinalis* [17] (Supplementary Table S1).

#### 2.1.2. Bioinformatic Analysis of *Rh*MosBP, *Rh*MOP130A, *Rh*Man113A, and *Rh*Gal36A

The gene sequences of *Rh*MosBP (RHOM_RS11160, GenBank protein-id, AEN97401.1), *Rh*MOP130A (RHOM_RS11135, GenBank protein-id AEN97396.1), *Rh*Man113A (RHOM_RS14610, GenBank protein-id: AEN98108.1), and *Rh*Gal36A (RHOM_RS11175, GenBank protein-id: AEN97404.1) were collected from the GenBank database [31]; open reading frames (ORFs) were identified with the Expasy Translate tool [32]; and SignalP-6.0 [33] was used to predict signal peptides. Homology searches were conducted by BLASTp (NCBI) (https://blast.ncbi.nlm.nih.gov/Blast.cgi, accessed on 5 September 2022) against the Protein Data Bank (PDB). Multiple sequence alignments were carried out by using Clustal Omega (www.ebi.ac.uk/, accessed on 5 September 2022). The homology model for *Rh*MosBP was generated by using SWISS-MODEL [34] with *Bl*MnBP (PDB 6I5R) as a template. The model structure was evaluated by using the programs MolProbity [35] (molprobity.biochem.duke.edu/, accessed on 5 September 2022) and PROCHECK [36] (www.ebi.ac.uk/thornton-srv/software/PROCHECK/, accessed on 5 September 2022), resulting in low QMEAN (<|2|), and few Ramachandran outliers (<0.95%). PyMOL (Version 2.0, Schrödinger, LLC, New York, NY, USA) was used for structure superimposition and figure generation (pymol.org/2/, accessed on 5 September 2022).

### 2.2. Strains and Media

*Bifidobacterium adolescentis* E194a ATCC 15703, which was isolated from the human adult intestine, and *Roseburia hominis* A2-183 DSMZ 16839, which was isolated from a human fecal sample, were purchased from American Type Culture Collection (ATCC; Manassas, VA, USA) and German Collection of Microorganisms and Cell Cultures, GmbH (The Leibniz Institute; DSMZ, Braunschweig, Germany), respectively. Stock cultures were stored at −80 °C in a reinforced clostridial medium (RCM, Oxoid, Hampshire, UK) containing 25% (*v*/*v*) glycerol. *B. adolescentis* and *R. hominis* were cultivated anaerobically, using a medium for colon bacteria (MCB) and a modified medium for colon bacteria (mMCB), respectively [16]. For coculture studies, MCB was used. Both MCB and mMCB have the same composition, except that mMCB is enriched with sodium acetate (5 g/L). All cultivation studies were carried out in triplicates.

### 2.3. Monoculture and Coculture Fermentation Studies

Filter sterilized (0.22 µm syringe filter) glucose, mannose, galactose, and (galacto)-β-mannan-oligosaccharides (MOS/GMOSs) at 10 g/L were used as carbohydrate sources. The MOS/GMOSs were produced in-house by enzymatic hydrolysis of low-viscosity locust bean gum (lvLBG) (Megazyme, Bray, Ireland), using a β-mannanase (*Bo*Man26B) from *Bacteroides ovatus*; the procedure and characterization of the preparation have been published before [16]. The growth medium was prepared, dispersed under nitrogen, and autoclaved at 121 °C for 20 min. For the cultivation of *B*. *adolescentis* or *R. hominis*, precultures were passaged three times on MCB or mMCB containing glucose or MOS/GMOS (details provided in Supplementary Materials Text S1). A 2.0% (*v*/*v*) preculture of *B. adolescentis* or *R. hominis* was used to inoculate 100 mL of MCB or mMCB for monocultivations. For cocultivation, 2% (*v*/*v*) values of both *B. adolescentis* and *R. hominis* were used as preculture for inoculating 100 mL of MCB. All fermentation studies were carried out at 37 °C for 48 h, without shaking in screw-capped vials in triplicates (referred to as biological replicates) [16]. Aliquots were collected after 0, 3, 6, 9, 12, 16, 24, 36, and 48 h of cultivation and were centrifuged at 8000× *g* for 15 min at 4 °C. For determining extracellular α-galactosidase and β-mannosidase activity, 100 µL of the cell-free supernatant was incubated with 0.9 mL of 1.0 mM para-nitrophenyl α-galactopyranoside (pNP-Gal) or para-nitrophenyl β-mannopyranoside (pNP-Man) (Sigma Aldrich, St. Louis, MO, USA) in 0.05 M phosphate buffer, pH 6.8. The reaction mixture was incubated at 37 °C for 10 min, and the reaction was stopped by adding 0.5 mL 1 M sodium carbonate, and the absorbance was measured at 405 nm. The rest of the supernatant was stored at −20 °C for carbohydrate utilization and short-chain fatty acids (SCFAs) analysis. The cell pellets were stored at −80 °C for genomic DNA (gDNA) extraction, quantitative PCR (qPCR), and gene-expression analysis.

### 2.4. Primer Design

Primer sets for determining the cell concentration of *B. adolescentis* and *R. hominis* by qPCR in mono- and cocultures were designed (Supplementary Table S2), and those used for amplification of reference genes and target genes in *R. hominis* by reverse-transcription-quantitative PCR (RT-qPCR) are listed in Supplementary Table S3. The primers were designed by using Primer3 plus software [37] and were based on the whole-genome sequences of *Roseburia hominis* A2-183 DSMZ 16839 (BioProject accession number PRJNA33399, RefSeq accession number NC_015977.1) and *Bifidobacterium adolescentis* E194a ATCC 15703 (BioProject accession number PRJNA16321, RefSeq accession number NC_008618.1). The in vitro confirmation of primer specificity and selectivity was performed by the qPCR analysis of gDNA from both the strains for evaluating population dynamics (Supplementary Table S2) and with gDNA from *R. hominis* for gene expression analysis (Supplementary Table S3). The details for qPCR amplification are mentioned below, in Section 2.5.2.

### 2.5. Bacterial Growth Analysis

The optical density at 600 nm (OD_600_) was determined by using a cell densitometer. All measurements were carried out in triplicates. The cell concentration of *R. hominis* and *B. adolescentis* during monocultivation and cocultivation on glucose and MOS/GMOS were quantified by using strain-specific qPCR targeting the *rho* gene (locus tag: RHOM_RS09380) encoding the RNA polymerase beta subunit protein and *rec*A gene (locus tag: BAD_RS05455) encoding the recombination protein, respectively (Supplementary Table S2).

#### 2.5.1. Genomic DNA Extraction

A culture volume of 2 mL was used for the extraction of gDNA, using a GeneJET Genomic DNA Purification kit (K0721, ThermoFisher Scientific, Waltham, MA, USA) according to the manufacturer’s protocol for Gram-positive bacteria, but with minor modifications [28], including the addition of 20 U/mL mutanolysin (Sigma-Aldrich, St Louis, MO, USA) and 30 mg/mL lysozyme (Sigma-Aldrich, St Louis, MO, USA) in the lysis buffer and an additional incubation (37 °C for 60 min) with 2.0 µg/mL DNase-free RNase (Sigma-Aldrich, St Louis, MO, USA). The concentration (absorbance at 260 nm) and purity (absorbance ratio, 260/280 nm) of the extracted gDNA were estimated by using a Nanodrop ND 1000 spectrophotometer (ThermoFisher Scientific, MA, USA), and the gDNA was stored in aliquots at −20 °C until further use.

#### 2.5.2. Quantitative PCR (qPCR)

The extracted DNA from mono- and coculture studies were analyzed by qPCR (CFX384 Touch Real-Time PCR, Bio-Rad, USA). A master mix (6.0 µL) containing 2× SsoAdvanced Universal SYBR Green Supermix (Bio-Rad, Hercules, MA, USA), primers at 300 nM (final concentration), and 4.0 µL (4.0 ng or 1.0 ng/µL) of the sample (gDNA) were mixed. Ten-fold serial dilutions of known quantities of *recA* and *rho* gene-amplification products (4.0 ng to 4.0 pg) were used as qPCR standards. The amplification included a denaturation step at 98 °C for 120 s, followed by 40 two-step cycles at 98 °C for 15 s and 58 °C for 30 s. A melt curve analysis (65–98 °C) was conducted at the end of the run. A template without gDNA was used as the negative control. The cycle threshold (C*_T_*) values were determined based on CFX Maestro software (Bio-Rad, Hercules, MA, USA). The *rho* or *rec*A copy numbers per mL were calculated based on C*_T_* values obtained during the qPCR analysis [38] (see Supplementary Materials Text S2 for details), using the corresponding standard curve. The standard curve was prepared by plotting the C*_T_* values obtained from qPCR analysis of the dilution series as a linear function of the logarithm of their calculated gene copy numbers per mL. The sum of calculated gene copy number values was used to determine the relative population of the different species [28,39]. Averages and standard deviations were calculated from three biological replicates and a technical duplicate of each biological replicate.

### 2.6. Determination of Carbohydrate Utilization and Short-Chain Fatty Acids (SCFAs) Production

#### 2.6.1. Utilization of Glucose and MOS/GMOS

The utilization of glucose and MOS/GMOS was determined during the growth of *R. hominis* and *B. adolescentis* as mono- and cocultures on these carbohydrate sources by analyzing the cell-free supernatant (see Section 2.3), using high-performance anion-exchange chromatography with pulsed amperometric detection (HPAEC–PAD), as described before [16,40] in Section 2.11

#### 2.6.2. Determination of Short-Chain Fatty Acids

The presence of SCFAs in the fermentation broth during the growth on different carbohydrate sources was determined as described by Bhattacharya et al. [41]. Briefly, cell-free supernatant (1 mL) was analyzed for lactic acid and SCFAs, including formate, propionate, acetate, and butyrate, by using high-performance liquid chromatography (HPLC) (Dionex Ultimate 3000, Thermo Fisher Scientific, MA, USA) equipped with an RI-detector (Shodex, New York, NY, USA). Values from control cultures without a carbohydrate source were subtracted.

### 2.7. Gene Expression Analyses

#### 2.7.1. RNA Extraction and Purification

Samples (8 mL) for RNA extraction were collected, and two volumes of RNA protect (Qiagen, Hilden, Germany) were added, mixed by vortexing, and then allowed to stand for 5 min at room temperature, followed by centrifugation at 10,000× *g* for 10 min. The supernatant was removed carefully, and the pellets were stored at −80 °C. Total RNA was extracted by using the RNeasy mini kit (Qiagen, Hilden, Germany) according to the manufacturer’s instructions, but with minor modifications. Cell pellets were suspended in 100 µL of Bioultra TE buffer (30 mM Tris base, 1 mM EDTA, pH 8.0) (Sigma-Aldrich, St. Louis, MO, USA) containing 30 mg/mL of lysozyme (Sigma-Aldrich, St. Louis, MO, USA), 20 mg/mL of proteinase K (Sigma-Aldrich, St. Louis, MO, USA), and 40 U/mL of mutanolysin (Sigma-Aldrich, St. Louis, MO, USA) and incubated for 45 min at 37 °C. Then 700 µL of RLT buffer (RNeasy kit, Qiagen, Hilden, Germany) was added to the cell suspension, vortexed, and mechanically disrupted by using a bead-beater with the Cryolys attachment (Precellys 24, Bertin technologies, Montigny-le-Bretonneux, France) maintained at 4 °C, using liquid nitrogen in 2 mL standard tubes containing 0.1 mm glass beads (Precellys Lysing Kit VK01, Bertin technologies, France). The tubes were subjected to a lysis program of 3 repetitions at 6500 rpm for 30 s, with an interval of 30 s. An on-column RNase-free DNase I (79254, Qiagen, Hilden, Germany) was used for removing DNA according to the manufacturer’s instructions. RNA was eluted with 60 µL of RNase-free water, and 2 µL of protector RNase inhibitor (Merck, Darmstadt, Germany) was added. The concentration (absorbance at 260 nm) and purity (absorbance ratio, 260/280 nm) of the extracted total RNA were estimated by using a Nanodrop ND 1000 spectrophotometer (ThermoFisher Scientific, Waltham, MA, USA).

#### 2.7.2. Monitoring Gene Expression

Reverse transcription of total RNA (diluted to a concentration of 100 ng/µL) into complementary DNA (cDNA) in a final volume of 20 µL was carried out with Revert Aid H Minus First Strand cDNA Synthesis Kit (K1632, ThermoFisher Scientific, MA, USA) as per the manufacturer’s instructions, using a Peltier thermal cycler PTC-200 (MJ Research, Waltham, MA, USA). For cDNA syntheses, the reaction was applied by using the following temperature profile: 40 °C for 5 min, 42 °C for 60 min, and 70 °C for 5 min. The cDNA samples were stored in aliquots at −80 °C. The qPCR analysis was carried out in a CFX384 Touch Real-Time PCR apparatus, Bio-Rad, USA. A master mix (6.0 µL) containing 2× Sso Advanced Universal SYBR Green Supermix (Bio-Rad, Hercules, CA, USA) and each primer at 300 nM final concentration was prepared, and 4.0 µL of the diluted cDNA (0.5 ng/µL). The qPCR amplification program consisted of an initial denaturation step at 95 °C for 20 s, followed by 40 two-step cycles at 95 °C for 10 s and 58 °C for 30 s. A melt curve analysis (65–98 °C) was conducted at the end of the run. In each run, gDNA from *R. hominis* was used as a positive control, while a no-template control (with nuclease-free water instead of cDNA) was used as the negative control. The C*_T_* values were determined based on CFX Maestro software.

#### 2.7.3. Relative Quantification of Gene Expression

Reference genes are used to normalize the variations in transcriptomic data that arise due to variations during the quantification of RNA or cDNA synthesis. For normalization, multiple reference genes (Supplementary Table S3) were screened, and their selection was based on four different computational programs: BestKeeper tool, which ranks the genes in agreement with the standard deviation of their C*_T_* values in correlation with intragroup alterations [42]; NormFinder tool, which evaluates gene stability, using both intragroup and intergroup changes [43]; geNorm tool, which calculates the stability of each gene through intragroup differences and mean pairwise variation [44]; and comparative delta C*_T_* method tool, which values the fluctuation of the delta C*_T_*, making a comparison between two or more reference genes [45]. The data were integrated to obtain a final rank, based on the geometric mean, using the RefFinder tool [46].

The expression of certain putative genes (locus tag: RHOM_RS11135, 11140, 11145, 11160, and 11175) within the (galacto)-β-mannan-oligosaccharides (MOS/GMOS) utilization locus, *Rh*MosUL (RHOM_RS11115-11180) and other putative target genes (locus tag: RHOM_RS14610, 05895, 06295, 13400, and 15425) spread across the *R. hominis* genome were analyzed (Supplementary Table S3). The level of expression of each target gene during the growth of this strain on glucose after 9 h was compared to its level of expression during the cultivation on glucose, mannose, galactose, and MOS/GMOS after 9 and 12 h of incubation and was expressed as the fold change on a logarithmic scale. The relative expression for each target gene was calculated based on the efficiency-corrected methodology, using multiple reference genes [47]. One-way ANOVA with Tukey’s post hoc test was used to determine whether the differential expression of the genes was significant during growth on different substrates.

### 2.8. Construction of Expression Plasmids for RhMOP130A, RhMan113A, and RhGal36A

The gene encoding *Rh*MOP130A was codon optimized for expression in *E. coli* BL21(DE3) star cells (Thermo Fischer, Waltham, MA, USA), synthesized, and then inserted between the *NcoI* and *NotI* restriction sites of the plasmid pET-52b(+) by Genscript (Piscataway, NJ, USA). Positioned immediately after the *NcoI* site, the *Rh*MOP130A gene was in frame with the plasmid-encoded start codon within the *NcoI* site. The native stop codon was replaced by a sequence encoding a Tobacco Etched Virus (TEV) protease site and subsequently followed by a plasmid-encoded Thrombin protease site, His_10_-tag, and stop codon. The TEV and Thrombin sites allow for the removal of the added His-tag but were not utilized within this work. The gene encoding *Rh*Man113A was similarly codon optimized for expression in *E. coli* BL21(DE3) star cells, synthesized, and then inserted between the *NcoI* and *NotI* sites of the plasmid pET-28b(+) by Genscript (Piscataway, NJ, USA). The *Rh*Man113A gene was positioned immediately after the *NcoI* site in frame with the plasmid-encoded start codon within the *NcoI* site. The native stop codon was replaced by a plasmid-encoded Thrombin protease site, His_6_-tag, and stop codon. Likewise, the gene encoding *Rh*Gal36A was codon optimized for expression in *E. coli* BL21(DE3) star cells, synthesized, and inserted between the *NcoI* and *NotI* sites of the plasmid pET-52b(+) by Genscript (Piscataway, NJ, USA). The *NcoI* start codon was replaced by the native one, followed by the coding region of the gene. The native stop codon was replaced by a plasmid-encoded Thrombin protease site, His_10_-tag, and stop codon. DNA sequencing was performed to verify the coding sequences and positions within the respective plasmids (Eurofins, Luxembourg). The plasmids were subsequently transformed into *E. coli* BL21(DE3) star cells.

### 2.9. Expression and Purification of RhMOP130A, RhMan113A, and RhGal36A

Transformed *E. coli* BL21 (DE3) star cells harboring the expression plasmid of either *Rh*MOP130A, *Rh*Man113A, or *Rh*Gal36A were cultured until mid-exponential phase (OD_600_~0.6) in 500 mL lysogeny broth (LB) medium at 37 °C, supplemented with 100 µg/mL ampicillin (for *Rh*MOP130A and *Rh*Gal36A) or 50 µg/mL kanamycin (*Rh*Man113A). Isopropyl β-D-thiogalactopyranoside (IPTG) was added (500 µM) to induce protein production, and the cultivation was continued for an additional 3 h. The cells were harvested by centrifugation and subsequently lysed by sonication or French press (*Rh*Man113A). Recombinant proteins were purified from collected cell lysates by immobilized metal ion affinity chromatography (IMAC), using a 5 mL HisTrap Ni Sepharose Fast flow column (Cytiva, Marlborough, MA, USA) in an NGC FPLC-system (Bio-Rad, Hercules, CA, USA). A 20 to 500 mM imidazole gradient was used for elution. Fractions of pure recombinant protein (assessed by SDS–PAGE) were pooled, and the buffer was exchanged by using Vivaspin 20 centrifugal concentrators of appropriate molecular cutoffs to 20 mM sodium phosphate buffer pH 6 (*Rh*Man113A), 20 mM TRIS-HCl pH 8 (*Rh*Gal36A), and 20 mM TRIS-HCl pH 7.4 (*Rh*MOP130A) with storage at +4 °C. Protein concentrations were determined by measuring the absorbance at 280 nm, using a nano-drop ND 1000 spectrophotometer and the recombinant protein extinction coefficients: 80,790 M^−1^cm^−1^ for *Rh*MOP130A, 92,820 M^−1^cm^−1^ for *Rh*Man113A, and 117,580 M^−1^cm^−1^ for *Rh*Gal36A, calculated by using ProtParam (https://web.expasy.org/protparam/, accessed on 5 September 2022).

### 2.10. Protein Electrophoresis of RhMOP130A, RhMan113A, and RhGal36A

Sodium dodecyl sulfate–polyacrylamide gel electrophoresis (SDS–PAGE) analysis was conducted as previously described in Bågenholm et al. [18], using either an unstained PageRuler protein ladder (for *Rh*Man113A and *Rh*Gal36A) (Thermo Scientific, Waltham, MA, U.S, product number: 26614) or a pre-stained PageRuler (*Rh*MOP130A) (26616, Thermo Scientific, Waltham, MA, USA) as reference. Electrophoresis was carried out by using 12% Mini-PROTEAN^®^ TGXTM Precast Protein gels (4561046, Bio-Rad, CA, USA) for 50 min at 150 V. Native polyacrylamide gel electrophoresis (native-PAGE) was conducted with Native PAGE marker (Thermo Fischer, Waltham, MA, USA, product number LC0725). Protein samples were loaded onto 4–15% Mini-PROTEAN^®^ TGXTM Precast Protein Gels (4561086, Bio-Rad, CA, USA), and the electrophoresis was run for 30 min at 150 V, using the manufacturer’s instructions.

### 2.11. Saccharide Analysis by High-Performance Anion Exchange Chromatography–Pulsed Amperometric Detection (HPAEC–PAD)

Analysis of mono- and oligosaccharides was performed by using HPAEC–PAD, using CarboPac PA20 (060142, Dionex CarboPac PA20 BioLC, 3 × 150 mm, ThermoFisher Scientific, Sunnyvale, CA, USA) and PA200 (062896, Dionex CarboPac Pa200 BioLC, 3 × 250 mm, ThermoFisher Scientific, Sunnyvale, CA, USA) analytical columns coupled to guard columns of the same material on a Dionex ICS-5000 system (ThermoFisher Scientific, Sunnyvale, CA, USA) according to previously described methods [16,40]. Standards of appropriate monosaccharides, including mannose (M1) or galactose (G1) (Sigma Aldrich, St Louis, MO, USA); linear mannan-oligosaccharides (MOS), including mannobiose (M2), mannotriose (M3), mannotetraose (M4), and mannopentaose (M5); and galactosylated mannan-oligosaccharides (GMOS), including galactosyl mannotriose (GM3) and di-galactosyl mannopentaose (G2M5), were used (Megazyme, Bray, Ireland) (2.5–50 µM mannose (M1) or galactose (G1) for PA20, 2.5–30 µM M1–M5 and GMOS for PA200). For the analysis of bacterial saccharide utilization, the elution was performed according to the previously described method optimized for fermentation media, using PA200 [16]. The analysis of monosaccharides (PA20) in enzyme incubations was according to [40], and for oligosaccharides, the PA 200 method described by Morrill et al. [48] was used, but with a slightly reduced time (10 instead of 15 min) for the sodium acetate gradient (0–50 mM), applied in 78 mM NaOH. An additional wash step (3 min, 200 mM NaOH) was added. Analytes were identified based on correlation with retention times of the known standards. Representative chromatograms from analyzed triplicate incubations are shown in the figures.

### 2.12. Enzyme Activity Characterization of RhMOP130A

The activity, stability, and products of *Rh*MOP130A were investigated in enzyme reactions set up in triplicates. The activity was determined by reverse phosphorolysis (synthesis) [49,50] by incubation (60 µL total reaction volume) of 0.14 mg/mL enzyme at 37 °C with α-D-mannose 1-phosphate (10 mM) and M4 (10 mM) in 100 mM sodium citrate buffer (pH 5.5, for pH-optimum pH 4.0–6.2). After 10 min, the reaction was stopped by boiling (5 min), and the released phosphate was quantified by using a colorimetric malachite green phosphate assay kit (MAK308, Sigma-Aldrich, St. Louis, MO, USA). The specific activity was determined by using either 10 mM glucose, M1, M2, M3, or M5 instead of M4. The stability of *Rh*MOP130A was determined by incubating the enzyme in 100 mM sodium citrate buffer (pH 5.5 and 6.0) at 37 °C and sampling for activity determination after 0, 1, 3, 5, 24, and 72 h. The activity of *Rh*MOP130A in the phosphorolysis reaction was determined by incubating 0.14 mg/mL of the enzyme in 100 mM sodium citrate buffer (pH 6.0) with 10 mM of either M2, M3, M4, M5, GM3, or G2M5 with 10 mM phosphate at 37 °C for 10 min (1 h and 24 h for the galactosyl mannosides) and analyzed by using HPAEC–PAD, as described above. The reactions were stopped by boiling (5 min) and were appropriately diluted with Milli-Q water and filtered through a 0.2 µM PTFE syringe filter into HPAEC vials. The detected phosphate release when using mannose 1-phosphate and oligosaccharides in synthesis activity determinations (as presented in the results section, Section 3.2.5, “Catalytic Properties of *Rh*MOP130A”) indicates that *Rh*MOP130A cleaves and phosphorylates terminal non-reducing mannosyl units by phosphorolysis, in accordance with other characterized mannan-oligosaccharide phosphorylases belonging to glycoside hydrolase (GH) family 130 [49]. Therefore, the assumed non-phosphorylated reaction product was quantified to determine the phosphorylase activity of the enzyme; for example, the release of M3 was used to assess the enzymatic phosphorylase activity against M4.

### 2.13. Enzyme Activity Characterization of RhMan113A

The activity, stability, and product formation of *Rh*Man113A were characterized in triplicate enzyme incubations. The activity of *Rh*Man113A towards the mannose containing polysaccharides galactomannan (Locust Bean Gum, Sigma-Aldrich, St. Louis, MO, USA) and glucomannan (Konjac, Low viscosity, MegaZyme, Bray, Ireland) was analyzed with the 3,5-dinitrosalicylic acid (DNS)-reducing sugar assay [18], using 84 µg/mL enzyme and 0.5% (*w*/*v*) substrate at 30 °C in 20 mM pH 6 sodium phosphate buffer and an extended time to 16 h. The specific activity assay of *Rh*Man113A towards different MOS and GMOS was conducted by analyzing the mannose release with HPEAC–PAD, using a PA20 column after incubating enzyme (5 µg/mL) with 5 mM substrate at 30 °C in 50 mM pH 6 sodium phosphate buffer for 10 min, terminated by boiling for 5 min. For product formation, the enzyme reaction time was extended with sampling at 0, 1, 3, and 24 h, and an analysis was carried out with a PA200 HPAEC column (enzyme loading for M5 was 37 µg/mL). The sampling time for product formation from M2 and GM3 was 24 h, with analysis, using the PA20 HPAEC-column. For pH optimum, the specific activity assay was used at various pHs (50 mM buffers) and up to 30 min. Sodium citrate buffer was used for pH 4–6 and sodium phosphate buffer for pH 6–8. The protein stability was determined with the specific activity assay after incubation of the enzyme for 0, 1, 3, and 24 h at 30 °C. Michaelis–Menten kinetics was conducted by following the specific activity assay with increasing substrate concentration from 1 to 20 mM and reaction times 30 min. The K_M_ and *k*_cat_ constants were obtained by nonlinear regression of the data, using GraphPad Prism 9 software (version 9.4.1) (GraphPad Software LLC, San Diego, CA, USA).

### 2.14. Enzyme Activity Characterization of RhGal36A

The α-galactosidase-specific activity of *Rh*Gal36A was assayed in triplicate, using 1 mM pNP-Gal, as described by Reddy et al. [51]. The appropriately diluted enzyme was incubated with the substrate in 50 mM citrate buffer pH 6 at 37 °C for 10 min. For the determination of the pH optimum, the specific activity assay was used with buffers of varying pH (50 mM citrate buffer for pH 4–6 and 50 mM phosphate buffer for pH 6–8, duplicate incubations at each pH). The specific activity of *Rh*Gal36A with oligosaccharide substrates was assessed by studying the galactose release with HPEAC–PAD, using a PA20 column after incubating triplicate reactions of the enzyme (5 µg/mL) with 5 mM galactosylated mannotriose (GM3) at 30 °C in 50 mM sodium citrate buffer pH 5.5 for 10 min and termination by boiling for 5 min. The same setup conditions were used for HPAEC–PAD analysis of product formation when 5 mM of GM3, G2M5, or raffinose was used as the substrate, but with sampling at 0, 10, and 30 min and 24 h and using a PA200 column. Raffinose incubations were further analyzed with a PA20 column for the separation of galactose from sucrose.

## 3. Results

### 3.1. Mono- and Cocultivation of Bifidobacterium adolescentis EB1a (ATCC 15703) and Roseburia hominis A2-183 (DSMZ 16839)

#### 3.1.1. Effect of Substrates on Bacterial Growth

**Figure 1:**
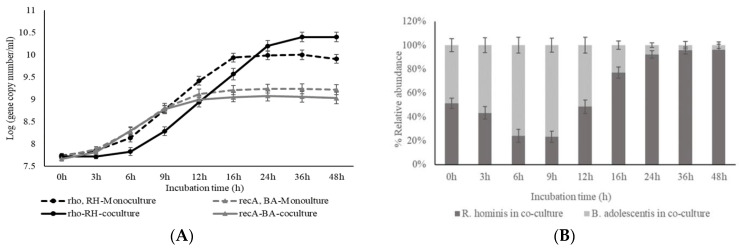

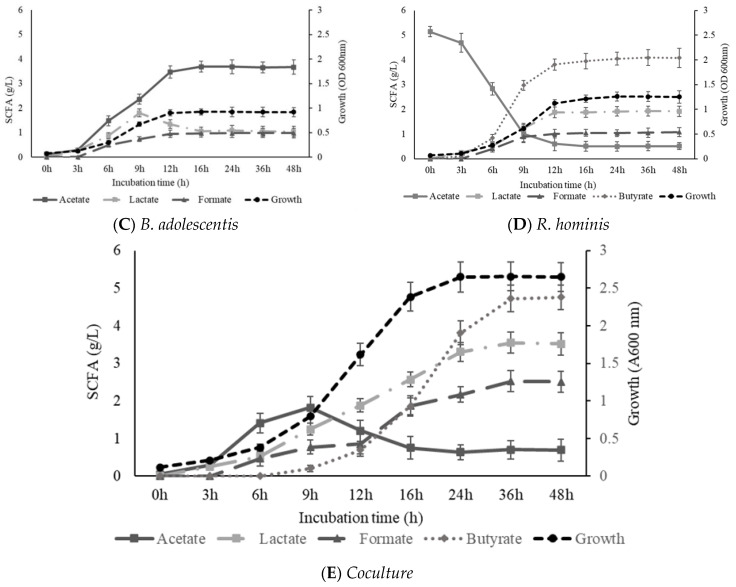
Growth and production of short-chain fatty acids (SCFAs) during cultivation of *Bifidobacterium adolescentis* (BA) and *Roseburia hominis* (RH) on (galacto)-β-mannan-oligosaccharide (MOS/GMOS). (**A**) Cell concentration by qPCR-analysis for BA and RH in mono- and cocultures. (**B**) Relative strain abundance (%) in the coculture. (**C**) The BA monocultivation (in the medium for colon bacteria, MCB). (**D**) The RH monocultivation (in the modified medium for colon bacteria, mMCB). (**E**) The cocultivation of RH and BA in MCB. *Rec*A (for BA) and *rho* (for RH) genes were used as markers in the qPCR-analysis to determine the cell concentration, expressed as log (copy number/mL) in the mono- and cocultures. The sum of calculated gene-copy-number values was used to determine the relative population (%) of RH and BA in the cocultures. The optical density (OD) was determined at 600 nm. The amount of SCFAs was determined by HPLC.

The growth (OD 600 nm) of *B. adolescentis* and *R. hominis* cultivated on glucose (10 g/L) as monocultures in the presence and absence of sodium acetate (5 g/L) indicated that, as expected, *R. hominis* is dependent on acetate [16], but *B. adolescentis* is not (Supplementary Figure S1). Further studies with *B. adolescentis* and *R. hominis* were carried out by using the medium for colon bacteria (MCB, without acetate) and a modified medium for colon bacteria (mMCB, with acetate), respectively. Coculture studies were carried out in the MCB medium. The cell concentrations of *R. hominis* and *B. adolescentis* were quantified by using strain-specific quantitative PCR (qPCR) targeting the *rho* gene (RHOM_RS09380) and *rec*A gene (BAD_RS05455), respectively (Figure 1A; Supplementary Figure S2A and Table S2). The growth curve of either strain during the monoculture fermentation of glucose was different compared to coculture fermentation (Supplementary Figure S2A). In monocultures, the highest cell concentration for *B. adolescentis* and *R. hominis* was determined to be 11.4 ± 0.2 log units (*rho* copy number mL^−1^) and 10.9 ± 0.1 log units (*rec*A copy number mL^−1^), respectively, after 16 h of incubation (Supplementary Figure S2A). However, during cocultivation, the highest cell concentration for *B. adolescentis* (10.3 ± 0.1 log units) and *R. hominis* (10.0 ± 0.1 log units) was observed after 12 h of fermentation (Supplementary Figure S2A), which was appreciably lower compared to monocultures. This potentially indicates competition for glucose between the two strains.

When (galacto)-β-mannan-oligosaccharides (MOS/GMOSs) were used as the substrate, the growth curves of *B. adolescentis* in mono- and cocultures were comparable (Figure 1A), reaching similar cell concentrations after 24 h (9.1–9.2 log units). However, a slightly higher cell concentration was observed for *R. hominis* after 24 h in coculture compared to monoculture fermentations (Figure 1A). Notably, compared to monocultures, the growth of *R. hominis* is considerably slower during the first 6 h of coculture on either glucose or MOS/GMOS (Supplementary Figure S2A and Figure 1A).

#### 3.1.2. Production of Short-Chain Fatty Acids (SCFAs) during Mono- and Cocultivation

Monocultivation of *B. adolescentis* on glucose resulted in the production of acetate (5.2 ± 0.5 g/L), followed by lactate (2.1 ± 0.4 g/L) and formate (0.31 ± 0.1 g/L), after 24 h (Supplementary Figure S2C). In the case of *R. hominis* monocultures, acetate consumption was accompanied by an appreciable production of butyrate (5.1 ± 0.4 g/L), followed by lactate (1.9 ± 0.3 g/L) and formate (1.0 ± 0.3 g/L), after 24 h (Supplementary Figure S2D).

When *B. adolescentis* was cultivated on MOS/GMOS, the concentration of acetate (3.7 ± 0.3 g/L) and formate (1.0 ± 0.2 g/L) increased up to 16 h and remained stable thereafter (Figure 1C); however, an increase in lactate (1.8 ± 0.2 g/L) was observed up to 9 h, followed by a significant decrease (1.1 ± 0.2 g/L) till 16 h of fermentation (Figure 1C). Such a decrease in lactate concentration was not observed when *B. adolescentis* was grown on glucose (Supplementary Figure S2C)**.** Reduced lactate formation was also observed previously when *B. longum* was grown on arabinoxylo-oligosaccharides, and it was proposed that, for certain *Bifidobacterium* spp., acetate, formate, and/or ethanol are formed at lactate’s expense, possibly as a response to high energy demands during growth on complex carbohydrates [28]. During monocultivation of *R. hominis* on MOS/GMOS, butyrate (4.1 ± 0.3 g/L) was the major organic acid to be produced, followed by lactate (1.9 ± 0.2 g/L) and formate (1.0 ± 0.1 g/L) after 24 h of fermentation (Figure 1D)**.**

In contrast to the monocultivation of *B. adolescentis*, during cocultivation with *R. hominis* on glucose or MOS/GMOS, a substantial decrease of acetate was observed after 9 h (Supplementary Figure S2 and Figure 1E). This phenomenon may potentially be associated with the initial dominance of *B. adolescentis* in coculture (Figure 1A,B and Supplementary Figure S2A,B), resulting in acetate production, which is then utilized by *R. hominis* (Figure 1E and Supplementary Figure S2E). This is further supported by the significant growth of *R. hominis* that was observed only after 3 or 6 h of cultivation on glucose (Supplementary Figure S2A) or MOS/GMOS (Figure 1A), respectively. This view is further supported by the fact that, during the fermentation of MOS/GMOS, a significant amount of butyrate was only detected in the fermentation medium after 12 h during cocultivation (Figure 1E) compared to 6 h in monocultivations (Figure 1D).

#### 3.1.3. Effect of Cocultivation on Glucose and MOS/GMOS Consumption

**Figure 2:**
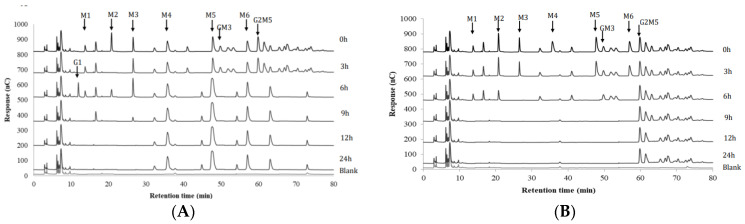

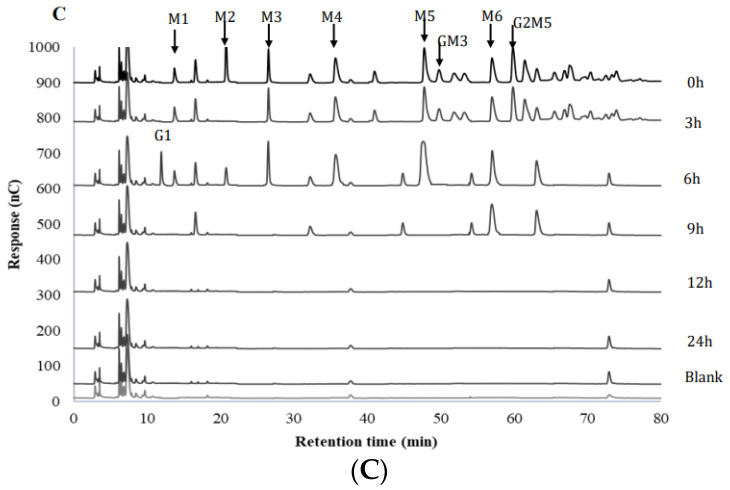
High-performance anion-exchange chromatography with pulsed amperometric detection (HPAEC–PAD) analysis of (galacto)-β-mannan-oligosaccharide (MOS/GMOS) utilization during the growth of (**A**) *B. adolescentis*, (**B**) *R. hominis*, (**C**) *B. adolescentis*, and *R. hominis* in coculture. The HPAEC–PAD analysis of the fermentation medium was carried out by using CarboPac PA200 column, as mentioned in Materials and Methods, in Section 2.11, Saccharide analysis by HPAEC–PAD. The elution was according to the previously described method optimized for fermentation media, as described previously in Bhattacharya et al. 2021 [16]. The arrows point to the retention time of galactose (G1), mannose (M1), linear mannan-oligosaccharides, MOS (mannobiose, M2, to mannohexaose, M6), and galactosylated mannan-oligosaccharides, GMOS (galactosyl mannotriose, GM3; di-glactosyl mannopentaose, G2M5) that have been used as standards in this study. All substrates were used at 5 g/L. An injection volume of 10 µL was used for all analytes.

To further understand the effect of cocultivation on the utilization of different carbohydrate sources, the utilization of glucose and MOS/GMOS in mono- and coculture fermentations were analyzed. The monocultivation on glucose indicated that both of the strains were able to efficiently utilize this monosaccharide (Supplementary Figure S2F) and thus would potentially compete for glucose during cocultivation. That this is the case is indicated by the disappearance of glucose within 9 h of cocultivation (Supplementary Figure S2F), along with lower growth of both strains compared to their monocultures (Supplementary Figure S2A). The utilization profile of MOS/GMOS in monocultures of *B. adolescentis* and *R. hominis* has previously been studied by us [16] and was repeated in the current study (Figure 2A,B). The results mirrored our previous findings, wherein *R. hominis* exhibited a preference for linear β-mannan-oligosaccharides, MOS (M2–M6), and certain galactosylated β-mannan-oligosaccharides, GMOS (GM3) [16]. *B. adolescentis* could internalize MOS with a degree of polymerization (DP) of 2–3 (M2–M3) and take up galactose possibly resulting from the enzymatic hydrolysis of GMOS by an extracellular α-galactosidase [16]. Interestingly, the MOS/GMOS utilization profile observed in the coculture (Figure 2C) is different when compared to monocultures of either strain (Figure 2A,B). The detection of galactose and uptake of mannobiose (M2) and mannotriose (M3) during the initial stages of coculture fermentations (0–6 h) are consistent with the profile observed for *B. adolescentis* monocultures, whereas the disappearance of mannotetraose (M4), mannopentaose (M5), and mannohexaose (M6) after 12 h of fermentation is not (Figure 2A). However, these M4–M6 can be utilized by *R. hominis* (Figure 2B) [16]. Remarkably, most of the detectable oligosaccharides disappeared from the fermentation medium in the coculture (Figure 2C) after 12 h of incubation, which was in stark contrast to monocultures of either strain (Figure 2A,B).

To understand the presence of galactose in the fermentation medium during the growth of *B. adolescentis* on MOS/GMOS in monoculture (Figure 2A) and in coculture (Figure 2C), we investigated the extracellular fractions for α-galactosidase activity using the pNP-α-galactopyranoside assay. A substantial amount of α-galactosidase activity (5.3–8.9 U/mL) was determined in the extracellular fractions after 6, 9, and 12 h fermentation of MOS/GMOS in *B. adolescentis* monoculture and during cocultivation. No α-galactosidase activity was detected in extracellular fractions of *R. hominis* (Supplementary Figure S3A). Moreover, after incubating the 9 h extracellular fractions from *B. adolescentis* and *R. hominis* monocultures with galactosyl-mannotriose (GM3) and di-galactosyl-mannopentaose (G2M5), galactose release was observed only with the extracellular fraction of *B. adolescentis* (Supplementary Figure S3B), suggesting that an extracellular α-galactosidase of *B. adolescentis* hydrolyzes these substrates. No β-mannosidase activity using pNP- β-mannopyranoside was detected in any of the extracellular fractions.

### 3.2. Role of the Locus RhMosUL and Other Genes in MOS/GMOS Utilization

To understand the mechanism behind the comparably efficient growth of *R. hominis* on MOS/GMOS and their utilization, we investigated the role of the putative (galacto)-β-mannan-oligosaccharides (MOS/GMOSs) utilization locus, *Rh*MosUL, and other distal genes in the potential utilization of MOS/GMOSs.

#### 3.2.1. Bioinformatic Analysis

**Figure 3:**
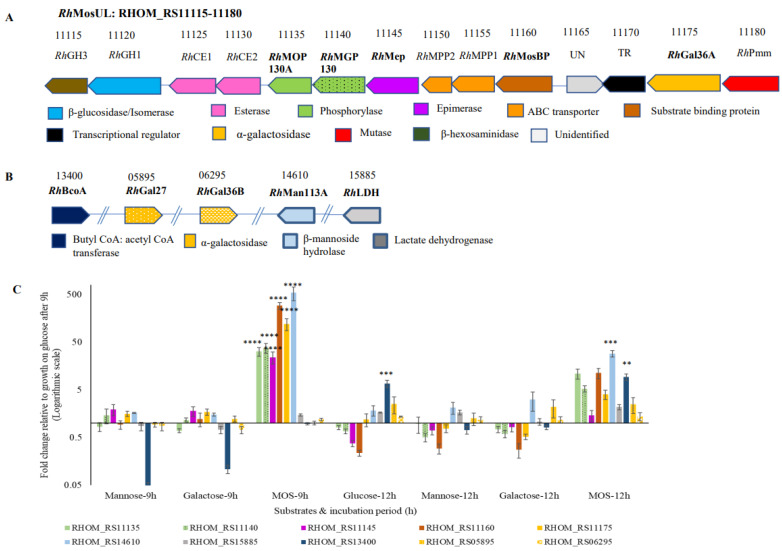
Gene expression analysis, using reverse transcription-quantitative PCR (RT-qPCR) during the growth of *R. hominis* on different substrates (glucose, mannose, galactose, and (galacto)-β-mannan-oligosaccharide, MOS/GMOS). (**A**) A putative MOS/GMOS utilization locus (*Rh*MosUL, RHOM_RS11115-11180) is predicted to be involved in MOS/GMOS utilization. (**B**) Other selected putative genes distributed across the genome were predicted to potentially be involved in MOS/GMOS utilization and short-chain fatty acid (SCFA) production. (**C**) Relative expression of 10 target genes expressed as fold change (logarithmic) relative to growth on glucose after 9 h. The putative function of the target genes RHOM_RS11135, RHOM_RS11140, RHOM_RS11145, RHOM_RS11160, RHOM_RS11175, RHOM_RS14610, RHOM_RS15885, RHOM_RS06425, and RHOM_RS13400 was functionally predicted as β-mannoside phosphorylase (*Rh*MOP130A), mannosylglucose phosphorylase (*Rh*MGP130), epimerase (*Rh*Mep), ABC substrate-binding protein (*Rh*MosBP), α-galactosidase (*Rh*Gal36A), β-mannoside hydrolase (RhGH113A), lactate dehydrogenase (*Rh*LDH), and butyl CoA: acetate CoA transferase (*Rh*BCoA). One-way ANOVA with Tukey’s post hoc test was used to determine whether the differential expression of the genes was significant during growth on different substrates over two different time points (9 and 12 h). The significantly different means are shown as ** *p* < 0.01, *** *p* < 0.001, and **** *p* < 0.0001. Data from triplicate cultivations and mean values ± SD (standard deviation) are presented.

Based on amino acid sequence similarity with a previously characterized β-mannan utilization locus in *Roseburia intestinalis* L1-82 (*Ri*MULL) [17], putative genes encoding potential proteins involved in the utilization of MOS/GMOSs were identified to be present in the putative *R. hominis* MOS/GMOS utilization locus (*Rh*MosUL, RHOM_RS11115-11180) (Figure 3A and Supplementary Table S1) with additional relevant genes for this function distributed across the genome (Figure 3B and Supplementary Table S1)**.** The *Rh*MosUL harbors fourteen putative genes, apparently encoding an ABC substrate-binding protein (*Rh*MosBP), components of an ABC transporter (*Rh*MPP1 and *Rh*MPP2), a regulatory protein (TR), an unidentified protein, and nine putative enzymes. These predicted enzymes include a β-hexosaminidase belonging to glycoside hydrolase (GH) family 3 (*Rh*GH3), a bifunctional protein (*Rh*GH1-M6P) with an N-terminal β-glucosidase domain (GH1) and C terminal mannose 6-phosphate isomerase domain, two carbohydrate esterases (*Rh*CE1 and *Rh*CE2), a β-mannoside phosphorylase (GH130_2) (*Rh*MOP130A), a mannosylglucose phosphorylase (GH130_1) (*Rh*MGP130), an epimerase (*Rh*Mep), an α-galactosidase (GH36) (*Rh*Gal36A), and a phosphomannomutase (*Rh*Pmm) (Figure 3A and Supplementary Table S1). Furthermore, genes encoding a putative GH113 β-mannoside hydrolase (*Rh*Man113A) and two other α-galactosidases belonging to GH36 (*Rh*Gal36B) and GH27 (*Rh*Gal27) were also identified elsewhere in the *R. hominis* A2-183 genome (Figure 3B and Supplementary Table S1). Additionally, genes predicted to encode lactate dehydrogenase (*Rh*LDH) and butyrate CoA: acetate CoA transferase (*Rh*BCoA) that play a key role in lactate and butyrate formation [28] were also identified in the *R. hominis* genome (Figure 3B and Supplementary Table S1).

#### 3.2.2. Gene Expression Analysis, Using Reverse Transcription-Quantitative PCR (RT-qPCR)

The role of *Rh*MosUL (five putative genes) and three other putative genes predicted to be involved in MOS/GMOS utilization was investigated based on expression analysis, using RT-qPCR. Moreover, the expression of two putative genes involved in butyric and lactic acid production was also analyzed. Therefore, for normalization of the transcriptomic data, six candidate reference genes in *R. hominis* were selected, and the variation in the gross expression data based on cycle threshold (C*_T_*) values were compared during growth on either glucose, mannose, galactose, or MOS/GMOS (Supplementary Table S3 and Figure S4A). These data were analyzed by four different statistical tools (Supplementary Figure S4B–E), and a comprehensive final ranking of the most stable reference genes was obtained by using RefFinder (Supplementary Figure S4F). This led to the selection of the three most stable genes, namely *rho*, *Dna*J, and *rpo*B, which were used to normalize the gene expression of the target genes. Thus, the expression of 10 target genes (Figure 3C and Supplementary Table S3) in *R. hominis* during growth on four different substrates (glucose, galactose, mannose, and MOS/GMOS) at two different time points (9 and 12 h) was evaluated. The C*_T_* values for all the target genes analyzed after 9 and 12 h of fermentation are illustrated in Supplementary Figures S5 and S6, respectively. The five target genes which are part of the locus *Rh*MosUL were all differentially expressed at significantly higher levels during growth on MOS/GMOS after 9 h compared to the monosaccharides (*p* < 0.0001) (Figure 3A,C). These genes encode *Rh*MOP130A (RHOM_RS11135), *Rh*MGP130 (RHOM_ RS 11140), *Rh*Mep (RHOM_ RS 11145), *Rh*MosBP (RHOM_RS11160), and *Rh*Gal36A (RHOM_RS11175). Similarly, the gene encoding *Rh*Man113A (RHOM_RS14610)*,* which is not part of the *Rh*MosUL, was also differentially expressed at a significantly higher level (Figure 3B,C). The genes coding for *Rh*BCoA (RHOM_ RS13400) and *Rh*LDH (RHOM_RS15885) were likewise significantly differentially expressed after 12 h of cultivation on MOS/GMOS (Figure 3B,C). Interestingly, the gene encoding *Rh*Gal36B (RHOM_RS06295) was found to be constitutively expressed during growth on all the substrates after 9 and 12 h (Supplementary Figures S5G and S6G; Figure 3) of fermentation, while the gene encoding *Rh*Gal27 (RHOM_RS05895) was not expressed (Supplementary Figures S5H and S6H; Figure 3).

#### 3.2.3. Bioinformatic Analysis of *Rh*MOP130A, *Rh*Man113A, *Rh*Gal36A, and *Rh*MosBP

Certain genes (RHOM_RS11135, RHOM_RS 11160, RHOM_RS11175, and RHOM_RS 14610) that were transcriptionally upregulated during growth on MOS/GMOS were further selected for biochemical and functional analysis of the proteins encoded by them. BLASTp analyses of the sequences of *Rh*MOP130A, *Rh*Man113A, *Rh*Gal36, and *Rh*MosBP against the PDB database supported the respective putative classification and function of the respective protein (Supplementary Table S4). The top hit for *Rh*MOP130A was a *Thermotoga* GH130 mannoside-phosphorylase (*tm*1225) acting on mannan-oligosaccharides (61.5% identity) [52]. For *Rh*Man113A and *Rh*Gal36A, a β-1,4-mannanase (*Ax*Man113A) from *Amphibacillus xylanus* (51.0%) [53] and a GH36 α-galactosidase (*Aga*B) from *Geobacillus stearothermophilus* (49.4%) [54] were the top hits, respectively (Supplementary Table S4). The top hit for *Rh*MosBP was a mannan-oligosaccharide binding protein (*Bl*MnBP1) of an ABC-transporter from *Bifidobacterium animalis* subsp. *lactis* ATCC 27673 [24] (Supplementary Table S4). The multiple sequence analysis with selected characterized homologs displayed the conservation of important catalytic residues in all three of the *R. hominis* enzymes (Supplementary Figure S7A–C). For *Rh*MosBP, all stacking tryptophans and several other polar mannotriose interacting residues of the bifidobacterial protein were conserved (Supplementary Figure S7D). The conservation of these residues was further supported by generating a homology model of *Rh*MosBP and superimposing it onto a crystal structure of *Bl*MnBP1 in a complex with mannopentaose [24] (Supplementary Figure S8). This indicates that *Rh*MosBP possibly also has a MOS-binding function and is involved in the MOS/GMOS utilization.

#### 3.2.4. Expression and Purification of *Rh*MOP130A, *Rh*Man113A, and *Rh*Gal36A

To functionally investigate the predicted key enzymes involved in the initial hydrolysis of MOS/GMOS, the coding DNA sequences of *Rh*Man113A, *Rh*Gal36A, and *Rh*MOP130A were inserted in plasmids, equipped with codons for His-affinity tags (Supplementary Figure S9). The recombinant proteins were expressed in *E. coli*, purified, and analyzed by SDS–PAGE (Supplementary Figure S10A–C), resulting in bands of apparent molecular weights, agreeing with the expected molecular weights of the His-tagged proteins (38.6, 86.1, and 42.3 kDa, respectively). Analyzed by native-PAGE, *Rh*MOP130A and *Rh*Gal36A showed bands at 232 and 346 kDa, (5.5-fold- and 4.0-fold-higher values than the respective monomer) (Supplementary Figure S10D), indicating that the proteins are hexameric and tetrameric, respectively. This is in line with previous (but not all) quaternary structures reported for GH130 [55] and GH36 enzymes [51].

#### 3.2.5. Catalytic Properties of *Rh*MOP130A

**Figure 4:**
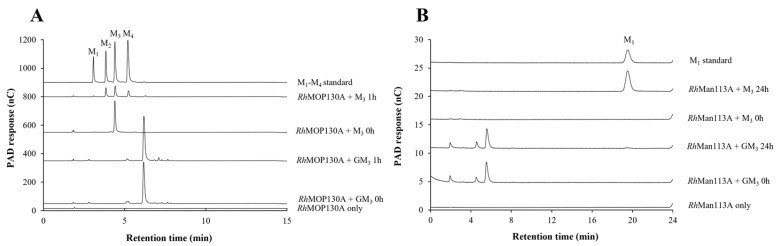
Incubation of mannotriose and galactosyl mannotriose with (**A**) *Rh*MOP130A or (**B**) *Rh*Man113A analyzed by anion-exchange chromatography with pulsed amperometric detection (HPAEC–PAD). (**A**) Column PA200 chromatograms of phosphorolysis incubations of *Rh*MOP130A (0.14 mg/mL) with 10 mM of either mannotriose (M3) or galactosylmannotriose (GM3). Standards of mannose (M1) to mannotetraose (M4) were applied for product quantification. Incubations were carried out at 37 °C for 0–1 h in 100 mM sodium citrate buffer (pH 6.0). (**B**) Column PA20-chromatograms for analysis of M1 release in incubations of *Rh*Man113A (5 µg/mL) with 5 mM of M3 or GM3. M1 was used as the standard for quantification. Incubations were carried out at 30 °C for 0–24 h in 50 mM sodium phosphate buffer (pH 6.0). Data from triplicate incubations and mean values ± SD (standard deviation) are presented.

The activity of the predicted β-mannoside phosphorylase *Rh*MOP130A was determined by using reverse phosphorolysis (synthesis), incubating α-mannose 1-phosphate and mannotetraose at pH 5.5. *Rh*MOP130A was stable (>95% remaining activity) over 72 h at 37 °C and pH 5.5. At pH 6, *Rh*MOP130A was stable (>95%) for 5 h. The activity was determined at various pH values, and the highest activity was obtained at pH 5.5, with a specific activity at 37 °C of 1.16 ± 0.11 kat/mol (Supplementary Figure S11A). Varying the acceptor saccharide, the highest specific activity was detected for M2 at 2.98 ± 0.1 kat/mol. *Rh*MOP130A displayed no activity towards either mannose or glucose, whilst showing similar specific activity with either M3, M4, and M5 when used as the acceptor saccharide (1.1–1.4 kat/mol, respectively) (Table 1). For phosphorolysis activity, *Rh*MOP130A was incubated (pH 6.0, 37 °C) with 10 mM phosphate and a mannan-oligosaccharide, using HPAEC for product detection (Supplementary Figure S12). No activity was detected towards M2 or without phosphate. The activity of *Rh*MOP130A peaked against M3 at 1.02 ± 0.13 kat/mol and was notably lower against M4 and M5 (Table 1). Thus, *Rh*MOP130A holds a preference for the same reaction complex consisting of three mannosyl units in either reaction direction (synthesis and phosphorolysis). The obtained phosphorolysis activities of *Rh*MOP130A are of the same order of magnitude as previously characterized gut bacterial GH130 enzymes analyzed with mannobiose or β-mannan [49,56]. The activity of *Rh*MOP130A towards MOS is in line with the activity of subfamily GH130_2 enzymes such as *Ra*MP, with which *Rh*MOP130A shows high identity (Supplementary Table S4), and contrasts with the specificity of GH130_1 mannoside hydrolases, which often act on shorter substrates, including mannosyl-glucose [56]. Interestingly, during HPEAC–PAD analysis of the phosphorolysis reactions, some of the generated reaction products appeared to have a higher degree of polymerization than the initial substrate (Supplementary Figure S12), indicating synthesis activity (without the addition of α-mannose 1-phosphate). When *Rh*MOP130A was incubated with GM3 or G2M5 for phosphorolysis as above (but with extended time), no substrate consumption or product peaks were detected by HPAEC–PAD (Figure 4A and Supplementary Figure S13), suggesting that this mannan-oligosaccharide phosphorylase is inactive against galactosylated mannan-oligosaccharides, in contrast to undecorated saccharides (Table 1).

#### 3.2.6. Catalytic Properties of *Rh*Man113A

**Figure 5:**
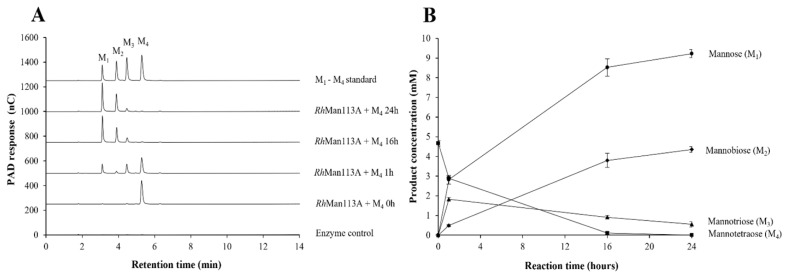
Progression of mannotetraose (M4) hydrolysis catalyzed by *Rh*Man113A. *Rh*Man113A (5 µg/mL) was incubated with 5 mM M4 at 30 °C in 50 mM sodium phosphate buffer (pH 6.0). (**A**) HPEAC–PAD analysis of hydrolysis reactions sampled over time. Mannose (M1) and mannan-oligosaccharides mannobiose to mannotetraose (M2–M4) were used as standards. (**B**) Profile of generated and consumed reaction products by *Rh*Man113A over time. Data from triplicate incubations and mean values ± SD (standard deviation) are presented.

Guided by the homology with GH113 endo-β-mannanases (Supplementary Table S4), *Rh*Man113A was initially incubated with polymeric β-mannans. The activity was very low, ≤0.02 katal/mol for locust bean gum galactomannan and ≤0.01 for konjac glucomannan at pH 6. Both *Aa*ManA [57] and *Ax*Man113A [53], two previously characterized endo-acting GH113 β-mannanases, show high activity towards locust bean gum (724.2 ± 20.0 U/mg, 22.4 ± 0.21 U/mg) and konjac glucomannan (1055.7 ± 26.2 U/mg, 54.4 ± 0.66 U/mg). When M4 was used as a substrate in incubations with *Rh*Man113A, higher activity was obtained (Table 2). At 30 °C and pH 6, *Rh*Man113A retained 95% of its activity towards M4 after 16 h, and approx. 80% was retained after 24 h. The HPAEC–PAD analysis of M4 incubations showed the formation of the hydrolytic reaction products M1, M2, and M3, with M1 being formed initially and the main reaction product over the course of the 24 h long reaction (Figure 5A). In contrast to M3, M2 was not consumed but instead accumulated, resulting in M1 and M2 as reaction end-products (Figure 5B).

*Rh*Man113A showed optimal activity at pH 6 (Supplementary Figure S11B). With oligosaccharides M2–M6, no detectable activity towards M2 was revealed, and M1 remained a dominant product in conversions, again ending with M1 and M2 as end-products. The specific activity (M1 formation at pH 6) was highest for M4 (10.2 ± 0.2 katal/mol), with M5 being the second most favored substrate (7.0 ± 0.1 katal/mol) (Table 2 and Supplementary Figure S14). The specific activity towards M3 was 4.9 ± 0.02 katal/mol, and for M6, it was 5.8 ± 0.4 katal/mol. No activity was detected towards GM3 (Table 2 and Figure 4B), suggesting that a non-galactosylated reducing end mannosyl unit is crucial for the hydrolytic activity of *Rh*Man113A. *Rh*Man113A exhibited Michaelis–Menten kinetics (Supplementary Figure S15), with the catalytic efficiency (k_cat_/K_M_) towards M4 3.1 s^−1^mM^−1^ (K_M_ = 3.3 ± 1.1 mM, k_cat_ = 10.2 ± 1.1 s^−1^), and 1.5 s^−1^mM^−1^ towards M5 (K_M_ = 1 ± 1.8 mM, k_cat_ = 3.2 ± 0.7 s^−1^) (Table 2).

The dominant release of mannose suggests that *Rh*Man113A is an exo-oligomannosidase, an activity which, in addition to endo-mannanase, is included in GH113 [58] and observed for the homolog *Ri*GH113 of *R. intestinalis* [17]. The lack of activity towards GM3, furthermore, is consistent with an attack from the reducing end, as is the case for *Ri*GH113. However, *Ri*GH113 slowly degraded M2, whereas *Rh*Man113A seemingly cannot. In contrast to the endo-mannanase *Ax*Man113A [53], the specific activity towards mannooligosaccharides of *Rh*Man113A peaks for M4, with lower activity towards M5. A deviation that could further highlight the differences between endo- and exo-enzyme activity.

#### 3.2.7. Catalytic Properties of *Rh*Gal36A

The specific activity of *Rh*Gal36A towards para-nitrophenyl α-galactopyranoside (pNP-Gal) was determined to be 234.7 ± 2.9 kat/mol at pH 6 (Table 3), which was also the pH-optimum (Supplementary Figure S11C). The specific activity towards galactosylated mannotriose (GM3) was 17.1 ± 0.7 katal/mol (30 °C, pH 5.5) (Table 3 and Supplementary Figure S16), which is similar to the GH36 α-galactosidase *Bo*Gal36A of the mannan utilization locus of *Bacteroides ovatus* [51]. The *Rh*Gal36A product profiles of GM3 and G2M5 show the reaction progressions and the galactose removal over time (Supplementary Figure S16A,B). After 24 h, the α-galactose substituents of both substrates were fully removed, leaving galactose and undigested M3 and M5 as reaction end-products. The digestion of raffinose generated detectable amounts of galactose and sucrose first after 24 h (Supplementary Figure S16C,D), indicating that *Rh*Gal36A may be optimized for GMOS rather than raffinose. Thus, *Rh*Gal36A could not only release terminal galactose substituents from galactosylated mannotriose and raffinose, but also internal galactose units from di-galactosylated mannopentaose (Table 3). The latter property is commonly observed for GH27 α-galactosidases [59] but only rarely observed for GH36 α-galactosidases [51], including the corresponding *R. intestinalis* GH36 α-galactosidase [17]. The capacity of *Rh*Gal36A to release galactosyl units from galactosylated mannotriose would make the remaining saccharide accessible for the herein characterized mannan-oligosaccharide phosphorylase *Rh*MOP130A and the suggested exo-β-oligomannosidase *Rh*Man113A, since these enzymes appear to be restricted by galactose substituents in the substrate (Figure 4).

## 4. Discussion

The differential in vitro utilization of (galacto)-β-mannan-oligosaccharides (MOS/GMOSs) during monocultivation by two potentially health-promoting gut bacteria, *B. adolescentis* and *R. hominis,* was demonstrated by Bhattacharya et al. [16] and laid the foundation for the current study. Herein, we tested our hypothesis that cross-feeding and metabolic interactions occur between *B. adolescentis* and *R. hominis* during growth on MOS/GMOSs, and this is supported by the significantly lower acetate concentrations during cocultivation on MOS/GMOSs (Figure 1E) compared to *B. adolescentis* monocultures (Figure 1C)**.** The gut microbiome’s interaction can be both competitive and cooperative, including substrate and metabolite cross-feeding [22]. Some cocultivation studies involving dietary fibers such as inulin, xylan, and pectin have exemplified the cross-feeding of short-chain fatty acids (SCFAs) between different gut bacteria [23,60]. Notably, the importance of cross-feeding, particularly of acetate between bifidobacteria and butyrate producers, has been reported for the growth on inulin-type fructans [61] and arabinoxylo-oligosaccharides [28]. Our study expands the understanding and the potential prebiotic application of MOS/GMOSs as bifidogenic and butyrogenic agents and contributes to further insight into acetate sharing among *Bifidobacterium* spp. and *Roseburia* spp., using MOS/GMOSs.

In previous studies [16,41] and as reported in this study, acetate is essential for the growth of *R. hominis* (Supplementary Figure S1). Since the cocultures do not contain any external acetate source, the growth of *R. hominis* is dependent on the acetate provided by *B. adolescentis*. This is clearly reflected during cocultivation on MOS/GMOS since the increase in cell concentration of *R. hominis* was observed only after 6 h, which was accompanied by a substantial decrease in acetate concentrations in cocultures compared to monocultures of *B. adolescentis* (Figure 1). Similar results were observed during the growth on glucose (Supplementary Figure S2).

Interestingly, the growth of *B. adolescentis* in coculture fermentation of MOS/GMOS remains unaffected when compared to monoculture fermentations (Figure 1A)**,** even though *R. hominis* becomes the more abundant strain in coculture after 12 h of incubation (Figure 1A,B). A possible explanation could be that, although mannobiose (M2) and mannotriose (M3) can be utilized by both strains, the initial relative abundance of *B. adolescentis* in the coculture (0–6 h) (Figure 1B) most likely allows it to utilize most of the available M2 and M3 before *R. hominis* starts competing for these oligosaccharides, due to the acetate dependence of this strain as discussed above.

The cocultures indicate faster and more comprehensive utilization of MOS/GMOS when compared to the selective utilization by either strain in monocultures (Figure 2). As a concrete example of differential and non-competitive utilization of MOS/GMOS in cocultures is the fact that only *R. hominis* can utilize linear β-mannan-oligosaccharides (MOS) longer than M3, including mannotetraose (M5), mannopentaose (M5), and mannohexaose (M6) (Figure 2B), and it is, therefore, likely responsible for their utilization in coculture (Figure 2C). Interestingly, *R. hominis* seems to benefit from the extracellular α-galactosidase activity of *B. adolescentis* (Supplementary Figure S3), which appears to result in the formation of linear β-mannan-oligosaccharides from galactosylated β-mannan-oligosaccharides (GMOSs) (Figure 2C). The benefit to *R. hominis* is substantiated by the fact that a higher cell concentration of *R. hominis* was determined during coculture compared to monoculture fermentation of MOS/GMOS (Figure 1A)**.** Furthermore, the butyrate production was also clearly (20%) higher in coculture compared to the monoculture of *R. hominis* (Figure 1D,E).

These results, along with the fact that near complete utilization of detectable MOS/GMOS occurs during cocultivation (Figure 2C), lead us to suggest that the differential utilization of MOS/GMOS by *B*. *adolescentis* and *R. hominis* is an important element in their successful coexistence. This is in stark contrast to the coculture fermentation of glucose, wherein the cell concentration of both *R. hominis* and *B. adolescentis* were significantly lower compared to their monoculture fermentations (Supplementary Figure S2A), indicating competition for the same substrate. The results of this study provide strong support for our hypothesis regarding cross-feeding and metabolic interactions between the *R. hominis* and *B. adolescentis* during coculture fermentation of MOS/GMOS. Thus, our study describes cross-feeding from *B. adolescentis* to *R. hominis*. Unidirectional cross-feeding in general seems to be important and prevalent in gut bacteria [62].

Both monoculture and coculture studies strongly indicated that *R. hominis* is better adapted to MOS/GMOS utilization compared to *B. adolescentis* (Figure 1A), and this could be attributed to the presence of a dedicated MOS/GMOS utilization locus (*Rh*MosUL) in *R. hominis* (Figure 3A). To further understand the role of *Rh*MosUL in MOS/GMOS utilization, a gene expression analysis was undertaken during the fermentation of MOS/GMOS by *R. hominis*. The importance of *Rh*MosUL in MOS/GMOS utilization was reflected by the significant transcriptional upregulation of genes encoding for *Rh*MosBP, *Rh*MOP130A, *Rh*MOP130, *Rh*Mep, and *Rh*Gal36A belonging to the *Rh*MosUL after 9 h of fermentation (Figure 3A,C). Furthermore, significant upregulation of the gene encoding *Rh*Man113A, which is not part of the *Rh*MosUL, was also observed after 9 h of fermentation (Figure 3). The upregulation of these genes and the predicted protein functions are consistent with significant roles in the uptake and utilization of MOS with a degree of polymerization (DP) of 2–3, along with galactosyl mannotriose (GM3) (Figure 2). Furthermore, a high level of expression of the genes encoding for *Rh*LDH and *Rh*BCoA after 12 h of MOS/GMOS fermentation was in accordance with the significant production of lactate and butyrate, respectively (Figure 1D and Figure 3C).

In this context, the present study not only throws light on the role of *Rh*MosUL but also on the enzymatic machinery during the utilization of MOS/GMOS through the biochemical characterization of three enzymes, namely *Rh*MOP130A, *Rh*Man113A, and *Rh*Gal36A. The genes encoding these enzymes were found to be transcriptionally upregulated during growth on MOS/GMOS (Figure 3) and indeed most likely play an important role in the initial hydrolysis of MOS/GMOS in *R. hominis*. The characterization of proteins encoded by *Rh*MosUL further elucidates the mechanism of MOS/GMOS utilization by *R. hominis*. The characterization clearly displays functions that align with the uptake and utilization of MOS/GMOS. Sequence analysis (Supplementary Figure S7D) and homology modeling (Supplementary Figure S8) support that *Rh*MosBP is a MOS/GMOS binding protein of an ABC-transporter system [24]. The co-expression of the distal *Rh*Man113A gene with *Rh*MosUL genes and its functional characterization suggest that the role of this enzyme is to act together with *Rh*MosUL-encoded enzymes in the conversion of MOS. This contrasts with the homologous *Ri*GH113 with similar catalytic properties, but which is encoded within the mannan-utilization locus of *R. intestinalis* [17] and underlines that the enzymes encoded by saccharide-utilization loci may act in synergy with distally expressed enzymes. The carbohydrate utilization data demonstrate that MOS with DP up to 6 is utilized by *R. hominis* (in contrast to *B. adolescentis* which does not utilize M4–M6) (Figure 2). This is consistent with the catalytic specificities of *Rh*Man113A and *Rh*MOP130A. The hydrolytic exo-oligomannosidase *Rh*Man113A acts on M3-M6 and shows the highest catalytic capability towards M4 (Table 2). In contrast to *Ri*GH113 [17], it is inactive towards M2. The mannan-oligosaccharide phosphorylase *Rh*MOP130A is active on the MOS with DP 3–6, with a slight preference for M3 (Table 1). Although GM3 is utilized by *R. hominis* (Figure 2B), both *Rh*Man113A and *Rh*MOP130A are hindered by galactosyl side groups carried by mannan-oligosaccharides (Figure 4). However, the α-galactosidase *Rh*Gal36A was shown to de-galactosylate GM3 and G2M5 (Table 3), and it is highly likely that this enzyme acts in sequential synergy with *Rh*Man113A and/or *Rh*MOP130A, generating linear saccharides for them, in a similar fashion as observed with some other α-galactosidases, such as *Bo*Gal36A in the mannan polysaccharide utilization locus (PUL) from *Ba. ovatus* [18], *Ri*GH36 in *Ri*MULL from *R. intestinalis* L1-82 [17], and *Fp*GH36 in *Fp*MULL from *Faecalibacterium prausnitzii* SL3/3 [15].

**Figure 6:**
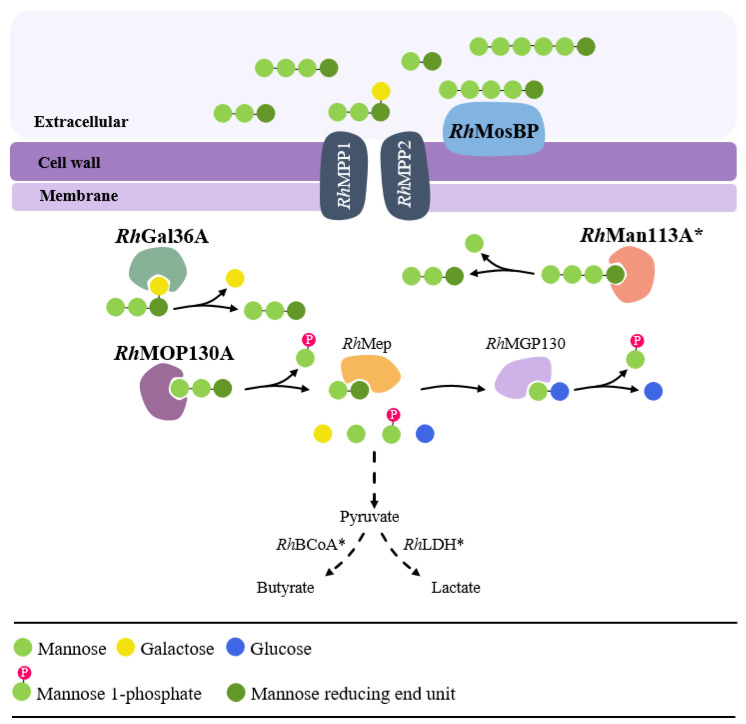
Schematic model of proposed (galactosyl-)mannan-oligosaccharide (MOS/GMOS) utilization mechanism of *R. hominis* A2-183. The figure shows a selection of proteins/enzymes proposed to be involved in the uptake and initial degradation of MOS/GMOS. All proteins and enzymes are encoded by the MOS/GMOS utilization locus (*Rh*MosUL, RHOM_RS11115-11180), except those marked with an asterisk (*). Gene products for which expression was studied and shown to be upregulated during growth on MOS/GMOS are in black text. Proteins and enzymes that were functionally investigated in this work are highlighted with bold text. The uptake of extracellular mannan-oligosaccharides (mannobiose to mannohexaose) and galactosyl-mannotriose is facilitated by ABC-transporter proteins, here exemplified by the solute-binding protein *Rh*MosBP and transport proteins (white text, not studied). Based on biochemical data, the α-galactosidase *Rh*Gal36A removes α-galactosyl substitutions making linear mannan-oligosaccharides available for depolymerization by the exo-oligomannosidase *Rh*Man113A and the mannan-oligosaccharide phosphorylase *Rh*MOP130A (phosphoryl shown in red). The dotted arrows illustrate several putative reactions.

Thus, neither of the characterized GH130 or GH113 enzymes convert mannobiose, which may be converted to mannosyl-glucose by the predicted epimerase *Rh*Mep, encoded from *Rh*MosUL, and transcriptionally upregulated during growth on the MOS/GMOS (Figure 3), in accordance with the β-mannan and MOS/GMOS utilization pathway characterized in *R. intestinalis* [17] and *F. prausnitzii* [15]. Furthermore, the mannosyl glucose could be converted by the similarly upregulated *Rh*MGP130, a predicted subfamily GH130_1 mannosyl glucose phosphorylase (Figure 3), as suggested in the above pathways, completing the conversion to monosugar and monosugar phosphate. A model for uptake and catabolism of MOS/GMOS by *R. hominis* A2-183 is presented in Figure 6.

Our study sheds light on part of a possible mechanism for polysaccharide and oligosaccharide utilization among certain gut bacteria. We used a β-mannanase, *Bo*Man26B from *Bacteroides ovatus*, to produce MOS/GMOS [16], which, in turn, was used as an oligosaccharide carbon source in this study. Notably, while *Ba. ovatus* is considered a primary degrader of many polysaccharides [22,23], *B. adolescentis* and *R. hominis* have been suggested to be secondary degraders, particularly for xylan and mannan [16,41]. Recent studies have highlighted the beneficial role of butyrate-producing gut bacteria [7] and underlined the potential role of *R. intestinalis* as a primary degrader of mannan [17] and *R. hominis* as a secondary degrader ([16], this study).

β-Mannans are naturally occurring polysaccharides that are commonly found in the human diet [12]. Logically, genomic analysis has indicated that β-mannan metabolism is prevalent within human gut microbiota [14]. This is further substantiated by the presence of the β-mannan polysaccharide utilization locus in *Ba. ovatus,* and *Ba. fragilis* [18,20], along with the recent identification of β-mannan utilization loci (*Ri*MULs) in *R. intestinalis* [17] and *Faecalibacterium prausnitzi* [15]. In this context, our study extends our understanding of β-mannan utilization by gut bacteria and highlights the role of *R. hominis* as an efficient secondary degrader of MOS/GMOS characterized by the presence of a dedicated MOS/GMOS utilization locus (*Rh*MosUL), along with a distally encoded exo-β-oligomannosidase.

## 5. Conclusions

In conclusion, microbiological, transcriptional, and biochemical analyses suggest that *R. hominis* A2-183 harbors a comprehensive apparatus for the utilization of MOS/GMOSs. This is in line with coculture studies involving *B. adolescentis,* wherein the ability to utilize a wide range of MOSs allows *R. hominis* to eventually become the more abundant strain, even though during the initial phase, growth is limited due to its dependence on acetate, which is provided by *B. adolescentis*. Moreover, the characterization of *Rh*MosUL-encoded *Rh*Gal36A and *Rh*MOP130A and distally encoded *Rh*Man113A indicated a synergistic action of these enzymes for initial internal hydrolysis of MOS/GMOSs, and this is substantiated by their simultaneous transcriptional upregulation during growth on MOS/GMOSs.

Overall, our study highlights the differential utilization of MOS/GMOSs and metabolite cross-feeding interactions between two health-promoting gut bacteria *B. adolescentis,* and *R. hominis*. Considering the complexity of the gut microbiome and competition for nutrients in the gut, our findings can contribute to the design of customized prebiotics, probiotics, and/or synbiotics for improving overall health by selective manipulation of gut microbiota.

## Figures and Tables

**Table 1:** The specific activity of the β-mannan-oligosaccharide phosphorylase *Rh*MOP130A in synthesis and phosphorolysis reaction directions.

	Specific Activity of *Rh*MOP130A (kat/mol)	
Substrate	Synthesis Direction	Phosphorolysis Direction
M1	-	NA
M2	2.98 ± 0.10	-
M3	1.13 ± 0.08	1.02 ± 0.13
M4	1.16 ± 0.11	0.65 ± 0.04
M5	1.45 ± 0.08	0.58 ± 0.02
GM3	NA	-
G2M5	NA	-

Enzyme activity was measured at 37 °C for 10 min in 50 mM sodium citrate buffer and 10 mM mannose 1-phosphate (synthesis reaction) or 100 mM sodium citrate buffer and 10 mM inorganic phosphate (phosphorolysis reaction). NA, not analyzed; “-”, no detectable activity. Commercially available mannose (M1), mannobiose (M2), mannotriose (M3), mannotetraose (M4), mannopentaose (M5), galactosyl mannotriose (GM3), and di-galactosyl mannopentaose (M5) were used as substrates. Data from triplicate incubations and mean values ± SD (standard deviation) are presented.

**Table 2:** Specific activity and enzyme kinetic parameters of *Rh*Man113A, using different substrates.

*Rh*Man113A				
Substrate	Specific Activity (kat/mol)	k_cat_ (s^−1^)	K_M_ (mM)	k_cat_/K_M_ (s^−1^ mM^−1^)
M2	-	NA	NA	NA
M3	4.9 ± 0.02	NA	NA	NA
M4	10.2 ± 0.2	10.2 ± 1.1	3.3 ± 1.1	3.1 ± 0.4
M5	7.0 ± 0.1	3.2 ± 0.7	2.1 ± 1.8	1.5 ± 0.9
M6	5.8 ± 0.4	NA	NA	NA
GM3	-	NA	NA	NA

Incubations were at 30 °C in 50 mM phosphate buffer. The assay time was 10 min for specific activity analysis and 30 min for Michaelis–Menten kinetics. NA, not analyzed; “-”, no detectable activity. Data from triplicate incubations and mean values ± SD (standard deviation) are presented.

**Table 3:** Activity of *Rh*Gal36A on different substrates.

Substrate	Specific Activity of *Rh*Gal36A in (kat/mol)
pNP-gal	234.7 ± 2.9
Raffinose	+
GM3	17.1 ± 0.7
G2M5	+

Incubations were at 30 °C in 50 mM sodium citrate buffer for 10 min (for determination of specific activity values) or 24 h (for detection of product formation as galactose release, using HPAEC–PAD with a PA20-column); “+”, activity detected on 24 h incubations. Data from triplicate incubations, mean values ± SD (standard deviation) are presented.

## Data Availability

Data is contained within the article or Supplementary Materials.

## Supplementary Materials

The following supporting information can be downloaded at https://www.mdpi.com/2076-2607/10/12/2496/s1. Table S1: Amino acid sequence similarity between *R. hominis* target proteins involved in this study with *R. intestinalis* and predicted functional annotation and localization of *R. hominis* proteins. Table S2: Primers designed and used in this study for quantitative PCR analysis (qPCR) in analysis of population dynamics. Table S3: Primers designed and used in this study for RT-qPCR analyses of reference and target genes in *Roseburia hominis* A2-183. Figure S1: Growth of *Roseburia hominis* (RH) and *Bifidobacterium adolescentis* (BA) on glucose (10 g/L) as mono- and coculture in the presence and absence of sodium acetate (5 g/L). Figure S2: Monitoring the growth, production of short-chain fatty acids (SCFAs) and glucose consumption during cultivation on glucose, as monoculture for *Bifidobacterium adolescentis*, BA (A, C, and F), *Roseburia hominis*, RH (A, D, and F), and as coculture (A, B, E, and F). Figure S3: Determining the extracellular (Ex) α-galactosidase activity in *B. adolescentis* (BA) and *R. hominis* (RH). Figure S4: Expression of reference genes and analysis of their stability in *R. hominis*. Figure S5: Expression data represented as cycle threshold (C*_T_*) values determined by using RT-qPCR for analysis of target genes during cultivation of *R. hominis* after 9 h of incubation. Figure S6: Expression data represented as cycle threshold (C*_T_*) values determined by using RT-qPCR for analysis of target genes during cultivation of *R. hominis* after 12 h of incubation. Table S4: Top 3 BLASTp results for selected MOS/GMOS utilization locus, *Rh*MosUL, encoded protein sequences against the Protein Data Bank (PDB). Figure S7: Multiple Sequence Alignment (MSA) with selected *R. hominis* protein sequences, *R. intestinalis* homologs and proteins obtained by the BLASTp search of the PDB database, as mentioned in Table S4. Figure S8: Predicted mannan-oligosaccharide binding site of homology model generated for *Rh*MosBP, using *Bl*MnBP1 (pdb: 6I5R) as a template. Figure S9: The amino acid sequences of the studied protein constructs (A) *Rh*MOP130A, (B) *Rh*Man113A, and (C) *Rh*Gal36A. Figure S10: Protein analysis by polyacrylamide gel electrophoresis. Figure S11: The effect of pH on enzyme activity. Figure S12: *Rh*MOP130A incubated with different mannan-oligosaccharides analyzed by HPAEC. Figure S13: *Rh*MOP130A incubated with G2M5 analyzed on a PA200 column, using HPAEC–PAD. Figure S14: HPEAC–PAD (PA20 column) analysis of the mannose release when *Rh*Man113A (5 µg/mL) was incubated with 5 mM mannan-oligosaccharides. Figure S15: Michaelis–Menten kinetics of *Rh*Man113A. Figure S16: Galactose release by *Rh*Gal36A when incubated with galactose-containing substrates. Supplementary text.
