# Supplementary material for: Cross-Feeding and Enzymatic Catabolism for Mannan-Oligosaccharide Utilization by the Butyrate-Producing Gut Bacterium Roseburia hominis A2-183
Source: Microorganisms. 2022 Dec 16;10(12):2496. doi: 10.3390/microorganisms10122496 (PMC9784577; doi:10.3390/microorganisms10122496)
Supplement: Supplementary file 1 [file microorganisms-10-02496-s001.zip › microorganisms-2015114-supplementary.pdf]

Supplementary Materials

Cross-Feeding and Enzymatic Catabolism for Mannan-Oligosaccharide Utilization by the Butyrate-Producing Gut Bacterium *Roseburia hominis* A2-183

Abhishek Bhattacharya [1,*,†], Lovisa Majtorp [1,†], Simon Birgersson [1], Mathias Wiemann [1], Krishnan Sreenivas [2], Phebe Verbrugghe [3], Olivier Van Aken [4], Ed W. J. Van Niel [2] and Henrik Stålbrand [1,*]

[1] Division of Biochemistry and Structural Biology, Department of Chemistry, Lund University, Naturvetarvägen 14, 221 00 Lund, Sweden
[2] Applied Microbiology, Department of Chemistry, Lund University, Naturvetarvägen 14, 221 00 Lund, Sweden
[3] Department of Food Technology, Engineering and Nutrition, Lund University, Naturvetarvägen 14, 221 00 Lund, Sweden
[4] Department of Biology, Lund University, Sölvegatan 35, 223 62 Lund, Sweden
* Correspondence: abhishek.bhattacharya@biochemistry.lu.se (A.B.); henrik.stalbrand@biochemistry.lu.se (H.S.)
† These authors contributed equally to this work.

Table S1. Amino acid sequence similarity between *R. hominis* target proteins involved in this study with *R. intestinalis* and predicted functional annotation and localisation of *R. hominis* proteins.

Roseburia hominis A2-183				*Roseburia intestinalis* L1-82		
**Locus tag/RefSeq protein id**	**GH families/Putative function**	**Predicted cellular location[4]**	**Protein Abbreviation**	**Query Coverage (%)/Identity (%)**	**Locus tag/GenBank protein id**	**Description/Function**
RHOM_RS11115/ WP_014080403.1[1]	GH3/ β-hexosaminidase	Transmembrane	*Rh*GH3	99.0/65.15	ROSINTL182_05537/ EEV02519.1	Predicted GH3/ β-hexosaminidase
RHOM_RS11120/ WP_014080404.1[1]	GH1/β-glucosidase /mannose-6-phosphate isomerase	Intracellular	*Rh*GH1-M6P	99.0/83.8	ROSINTL182_05469-70/VCV21232.1	GH1/ mannose-6-phosphate isomerase (Biochemically characterized)[5]
RHOM_RS11125/ WP_014080405.1[1]	CE1/acetyl esterase	Intracellular	*Rh*CE1	99.0/65.03	ROSINTL182_05471/ (6HH9_A)	Acetyl esterase (Biochemically characterized)[5]
RHOM_RS11130/ WP_014080406.1	CE2/acetyl esterase	Intracellular	*Rh*CE2	98.0/67.92	ROSINTL182_05473/ VCV21230.1	Acetyl esterase (Biochemically characterized)[5]
RHOM_RS11135/ WP_014080407.1[1]	GH130_2/β-mannoside phosphorylase	Intracellular	*Rh*MOP130A	100.0/94.41	ROSINTL182_05474/ EEV02551.1	GH130_2/ β-1,4-mannooligosaccharide phosphorylase (Biochemically characterized)[5]
RHOM_RS11140/ WP_014080408.1[1]	GH130_1/4-O-β-D-mannosyl-D-glucose phosphorylase	Intracellular	*Rh*MGP130	98.0/94.57	ROSINTL182_05475/ VCV21228.1	GH130_1/ 4-O-β -D-mannosyl-D-glucose phosphorylase (Biochemically characterized)[5]
RHOM_RS11145/ WP_014080409.1[1]	Mannobiose 2-epimerase	Intracellular	*Rh*Mep	99.0/77.6	ROSINTL182_05476/ EEV02553.1	Mannobiose 2-epimerase (Biochemically characterized)[5]
RHOM_RS11150/ WP_014080410.1[1]	ABC transporter permease protein	Transmembrane	*Rh*MPP1	100.0/79.9	ROSINTL182_05477/ EEV02554.1	ABC transporter permease protein
RHOM_RS11155/ WP_014080411.1[1]	ABC transporter permease protein	Transmembrane	*Rh*MPP2	94.0/77.2	ROSINTL182_05478/ EEV02555.1	ABC transporter permease protein

RHOM_RS11160/ WP_014080412.1[1]	ABC Substrate-binding protein	Secreted (signal) peptide	*Rh*MosBP	99.0/65.0	ROSINTL182_05479/ EEV02556.1	ABC Substrate-binding protein (Thermodynamically characterized)[5]
RHOM_RS11165/ WP_044024994.1[1]	Unidentified function	Intracellular	Unidentified protein	30.0/32.6	ROSINTL182_08687/ EEU99425.1	Transcriptional regulator
RHOM_RS11170/ WP_014080414.1[1]	Transcriptional regulator	Intracellular	TR	98.0/64.7	ROSINTL182_05480/ EEV02557.1	Transcriptional regulator
RHOM_RS11175/ WP_014080415.1[1]	GH36, α-galactosidase	Intracellular	*Rh*Gal36A	99.0/70.1	ROSINTL182_05481/ EEV02558.1	GH36, α-galactosidase (Biochemically characterized)[5]
RHOM_RS11180/WP_ 014080416.1[1]	Phosphomanno-mutase	Intracellular	*Rh*Pmm	99.0/91.4	ROSINTL182_05482/ EEV02559.1	Phosphomanno-mutase (Biochemically characterized)
RHOM_RS14610/ WP_014081071.1[2]	GH113A, β-mannoside hydrolase	Intracellular	*Rh*Man113A	96.0/59.9	ROSINTL182_05483/ EEV02560.1	GH113, β-mannanase (Biochemically characterized)[5]
RHOM_RS05895/ WP_044024878.1[2]	GH27, α-galactosidase	Intracellular	*Rh*Gal27	92.0/62.0	ROSINTL182_03125/ EEV02431.1	GH27, predicted α-galactosidase
RHOM_RS06295/ WP_014079449.1[2]	GH36, α-galactosidase	Intracellular	*Rh*Gal36B	98.0/69.9	ROSINTL182_05846/ EEV02235.1	GH36, predicted α-galactosidase
RHOM_RS13400/ WP_014080837.1[3]	Butyryl-CoA: acetate CoA-transferase	Intracellular	*Rh*BCoA	94.0/100	ROSINTL182_07121/ EEV00989.1	Predicted butyryl-CoA: acetate CoA-transferase
RHOM_RS15425/ WP_014081204.1[3]	L-lactate dehydrogenase	Intracellular	*Rh*LDH	98.0/65.3	ROSINTL182_05160/ EEV02890.1	Predicted L-lactate dehydrogenase

Proteins putatively involved in MOS/GMOS utilisation, encoded by locus [1]*Rh*MosUL (RHOM_RS11115- RS11180) or [2]distally were selected together with proteins putatively involved in synthesis of [3]short-chain fatty acids (SCFAs). [4]The presence of signal peptides and transmembrane sequences was carried out using Signal P6 (https://services.healthtech.dtu.dk/service.php?SignalP) and DeepSig (https://deepsig.biocomp.unibo.it/welcome/default/index), respectively. [5]Biochemical characterisation as reported in La Rosa et al., 2019.

The amino acid sequences were retrieved from the RefSeq protein database (https://www.ncbi.nlm.nih.gov/refseq/). BLASTp (BLOSUM 62-matrix) of NCBI (National Center for Biotechnology Information) was used for analysing the amino acid sequence similarity of target proteins in *R. hominis* A2-183 with *R. intestinalis* L1-82 using the default threshold e-value of 1e-5. The functional annotation for the *R. hominis* target proteins was predicted based on the high sequence similarity to the homologs in *R. intestinalis*. The GH family information was based on the Carbohydrate-Active enzymes (CAZy) database (http://www.cazy.org/bB.html). The proteins involved in SCFAs production were selected based on information obtained from Riviere et al., 2015 and the pathway database Kyoto Encyclopaedia of Genes and Genomes (KEGG) (https://www.genome.jp/kegg/) and BioCyc (https://www.biocyc.org/).

Table S2. Primers designed and used in this study for quantitative PCR analysis (qPCR) in analysis of population dynamics

**Organism**	**Locus tag**	**Primer sequences**		**Annealing temperature (° C)**	**Amplicon length (bp)**	**Efficiency (E)**	**C_T value, genomic DNA of *R. hominis***	**C_T value genomic DNA of *B. adolescentis***
		Forward primer (5'⟶3')	Reverse primer (5'⟶3')					
Roseburia hominis A2-183 (DSMZ 16839)	RHOM_RS09380, *rho*, Transcriptional termination factor	CGGAAGCAAGATGGACGAC	CGATGGCAGGGAACACAC	58	107	1.98	19.8	N.D
B. adolescentis ATCC 15703	BAD_RS05455*, rec*A, Recombination protein	GAAGGCGAGATGGGAGACAG	TGATGAAGATGGCGGTGGT	58	112	1.98	N.D	19.5

The primers were designed based on the whole genome sequences of *R. hominis* A2-183 DSMZ 16839 (BioProject accession number PRJNA33399) and *B. adolescentis* ATCC 15703 (BioProject accession number PRJNA16321). N.D, not determined by qPCR. Forward and reverse primers were used at 0.3µM. The primers were designed using Primer3 plus software (https://www.bioinformatics.nl/cgi-bin/primer3plus/primer3plus.cgi), the primer parameters (GC content, melting temperature, GC clamps, cross-dimers, self-dimers, hairpins) were checked using Beacon designer (http://www.premierbiosoft.com/molecularbeacons/), the *in-silico* analysis for gene and strain specificity was carried out using Primer blast (https://www.ncbi.nlm.nih.gov/tools/primer-blast/) and SnapGene (https://www.snapgene.com/). The primers were synthesized by GenScript (New Jersey, US). Sso Advanced Universal SYBR Green Supermix (Bio-Rad, Hercules, USA) was used.

Table S3. Primers designed and used in this study for RT-qPCR analyses of reference and target genes in *Roseburia hominis* A2-183

Organism	Locus tag/ abbreviation (putative function) [a]	Primer sequences [b]	Amplicon length (bp)	Amplicon length (bp)	Efficiency (E)[d]	C_T value, genomic DNA of *R. hominis*
		Forward primer (5'⟶3')	Reverse primer (5'⟶3')			
Roseburia hominis- Reference genes	RHOM_RS01035/ *EF-Tu* (Elongation factor-Tu)	TGCTCAGATGGATGGTGCT	GACGGGAAAGTAAGATGTG CT	86	1.96	19.5
	RHOM_RS04910/ *Dna*J (chaperone protein DnaJ)	ATTCCACCGCACAGATTTCC	CCAGGCTTCACTTCATACAA CA	97	1.96	19.3
	RHOM_RS00030/ *Gyr*A2 (DNA gyrase subunit A)	GAAGTGCTACAAGATTACCG AGA	CGTCCAAGAAGTGCCGTATC	138	1.95	19.1
	RHOM_RS09380/ *rho* (Transcriptional termination factor)	CGGAAGCAAGATGGACGAC	CGATGGCAGGGAACACAC	107	1.97	19.8
	RHOM_RS00995/ *rpo* (RNA polymerase beta subunit)	ACAACAAGGAGACTGACGA GA	TGGCTGACGATAACACGCT	115	1.97	20.0
	RHOM_RS14135/ *Sec*Y (Preprotein translocase)	ATTACCTGAACCGAATCCTG AA	ATACATCCGCACCAAACACA	108	1.96	20.7
Roseburia hominis- Target genes	RHOM_RS11135/ *Rh*MOP130A (β-mannoside phosphorylase)	GGAGATACAAGGAGAACCC GA	GCGGAAGACGCCGATGAA	111	1.95	20.3
	RHOM_RS11140/ *Rh*MGP130 (mannosylglucose phosphorylase)	CCCTGACACCAATCCGCAC	CGCCACGCCGAAGAAAGA	139	1.95	21.0
	RHOM_RS11145/*Rh*Mep (Epimerase)	TTACCAGAGAGTTCCAGCCG	GCAGGGTGTTCATCGTCTTA TC	81	1.96	19.3
	RHOM_RS11160/*Rh*MosBP (ABC-substrate binding protein)	AAGTGAGTATTTCCTGCCGT	TACCAGTCCGTCGTAATCCC	88	1.95	19.8
	RHOM_RS11175/*Rh*Gal36A (α-galactosidase)	GCACCGTTTGTTCCTTCAC	ATCGCACTCTCCCTGTAATC	107	1.97	20.4

	RHOM_RS14610/ *Rh*Man113 (β-mannoside hydrolase)	CAGGAACTTGACCGTATTGAAAG	ATTGTTAGGCACCATTGAGGAA	108	1.96	19.4
	RHOM_RS05895/*Rh*Gal27A (α-galactosidase)	GGGAAGAGAAGGAGAGAAGCA	ACTGTGGTGTCGTAATAGTCATAG	85	1.95	20.2
	RHOM_RS06295/*Rh*Gal36B (α-galactosidase)	CGGAGAATGGAGACAGGGCA	CCAGCGGAGCAACATCACAC	94	1.98	20.5
	RHOM_RS13400/ *Rh*BCoA (butyryl-CoA: acetate CoA-transferase)	GCAAAGGCAACACAGAAGG	ATGTAACCGTATCCACCAAACA	119	1.98	20.7
	RHOM_RS15425/ *Rh*LDH (lactate dehydrogenase)	GTTGCAGTAATCGGATGTGG	TGGCATCTATGAGCACCATC	94	1.96	20.

[a] The information for gene sequences were retrieved from RefSeq database (https://www.ncbi.nlm.nih.gov/refseq/). [b] Forward and reverse primers were used at 0.3µM. The primers were designed using Primer3 plus software (https://www.bioinformatics.nl/cgi-bin/primer3plus/primer3plus.cgi), the primer parameters (GC content, melting temperature, GC clamps, cross-dimers, self-dimers, hairpins) were checked using Beacon designer (http://www.premierbiosoft.com/molecularbeacons/), the *in-silico* analysis for gene and strain specificity was carried out using Primer blast (https://www.ncbi.nlm.nih.gov/tools/primer-blast/) and SnapGene (https://www.snapgene.com/). The primers were synthesized by GenScript (New Jersey, US). Sso Advanced Universal SYBR Green Supermix (Bio-Rad, Hercules, USA) was used. [c] *rpo*B, RNA polymerase beta subunit and *rec*A, Recombination protein from *R. hominis* and *B. adolescentis*, respectively, were also used *in vitro* for population dynamics analysis using genomic DNA **(Table S2).** [d] The E value shows the PCR amplification efficiency for each primer set.

Figure S1

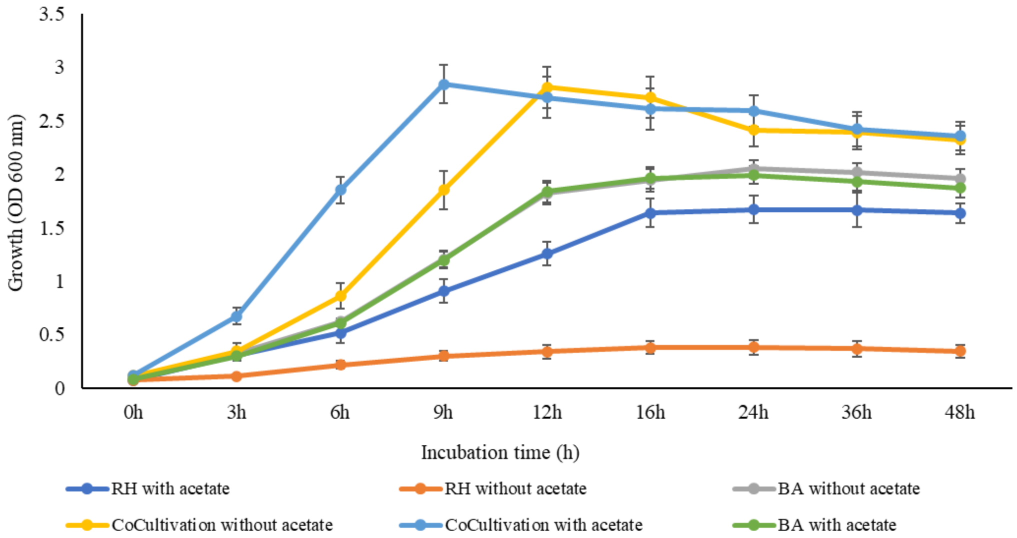

Figure S1. Growth of *Roseburia hominis* (RH) and *Bifidobacterium adolescentis* (BA) on glucose (10 g/L) as mono- and coculture in the presence and absence of sodium acetate (5 g/L). Cultivation (100 mL) for monoculture and coculture was carried out using MCB medium (without acetate) and mMCB medium (with acetate). All incubations were carried out at 37 ºC for 48 h under anaerobic conditions (Bhattacharya et al., 2021). Growth was monitored by removing aliquots and measuring the optical density at 600nm.

Figure S2

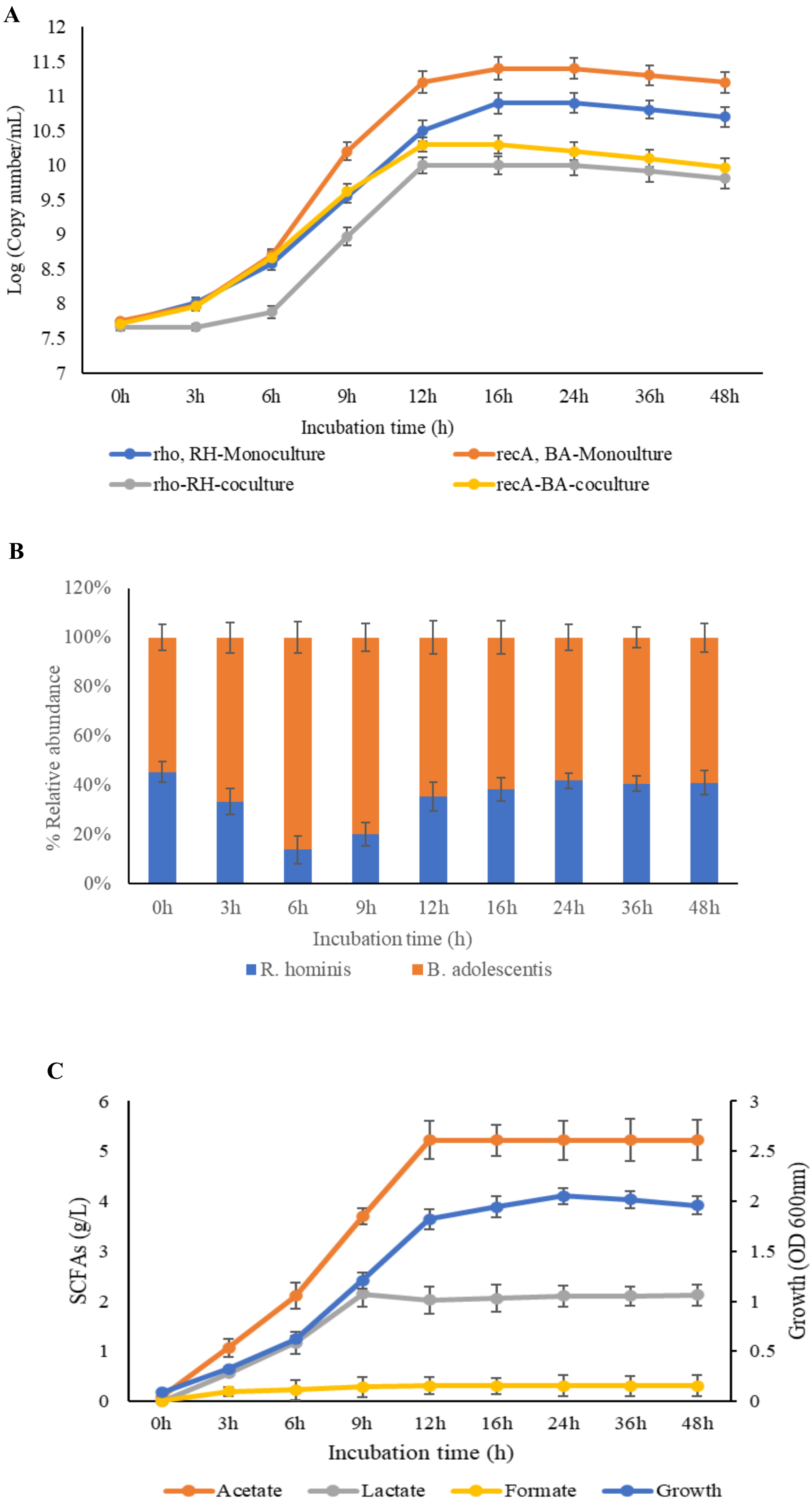
A
12
11.5
11
10.5
10
9.5
9
8.5
8
7.5
7
Log (Copy number/mL)
0h
3h
6h
9h
12h
16h
24h
36h
48h
Incubation time (h)
rho, RH-Monoculture
recA, BA-Monoulture
rho-RH-coculture
recA-BA-coculture
B
120%
100%
80%
60%
40%
20%
0%
% Relative abundance
0h
3h
6h
9h
12h
16h
24h
36h
48h
Incubation time (h)
R. hominis
B. adolescentis
C
6
5
4
3
2
1
0
SCFAs (g/L)
3
2.5
2
1.5
1
0.5
0
Growth (OD 600nm)
0h
3h
6h
9h
12h
16h
24h
36h
48h
Incubation time (h)
Acetate
Lactate
Formate
Growth

D

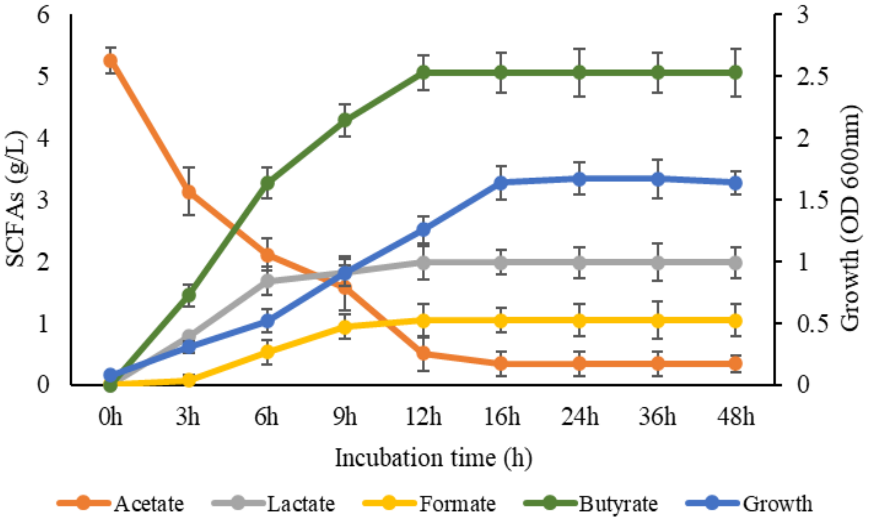
SCFAs (g/L)
Growth (OD 600nm)
Incubation time (h)
0h
3h
6h
9h
12h
16h
24h
36h
48h
Acetate
Lactate
Formate
Butyrate
Growth

E

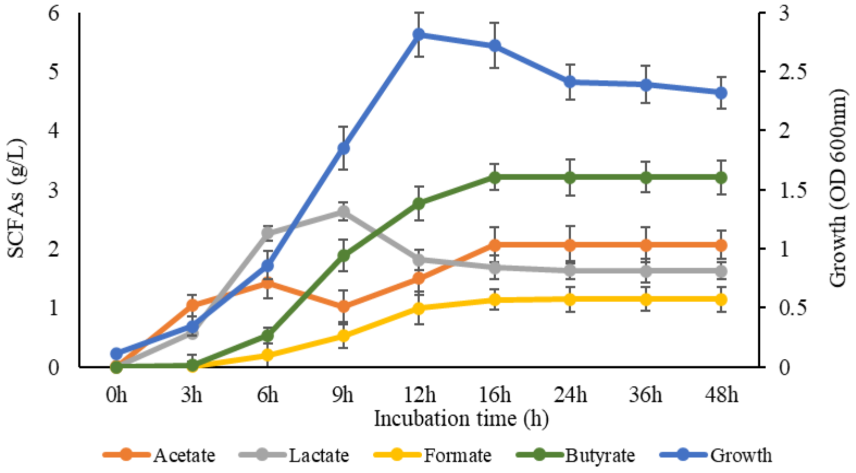
SCFAs (g/L)
Growth (OD 600nm)
Incubation time (h)
0h
3h
6h
9h
12h
16h
24h
36h
48h
Acetate
Lactate
Formate
Butyrate
Growth

F

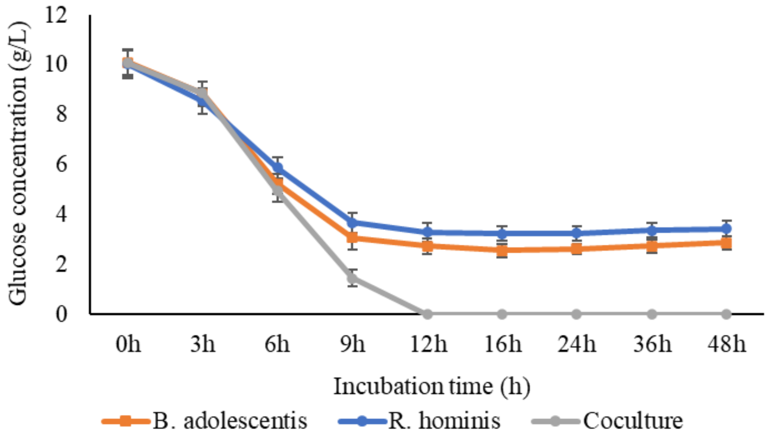
Glucose concentration (g/L)
Incubation time (h)
0h
3h
6h
9h
12h
16h
24h
36h
48h
B. adolescentis
R. hominis
Coculture

Figure S2. Growth and production of short-chain fatty acids (SCFAs) during cultivation of *Bifidobacterium adolescentis* (BA) and *Roseburia hominis* (RH) on glucose. (**A**) Cell concentration by qPCR-analysis for BA and RH in mono- and cocultures. (**B**) Relative strain abundance (%) in the coculture. (**C**) The BA mono-cultivation (in the medium for colon bacteria, MCB). (**D**) The RH mono-cultivation (in the modified medium for colon bacteria, mMCB). (**E**) The cocultivation of RH and BA (in MCB). (**F**) Consumption of glucose in the mono- and cocultures. RecA (for BA) and rho (for RH) genes were used as markers in the qPCR-analysis to determine the cell concentration, expressed as log (copy number/mL) in the mono- and cocultures. The sum of calculated gene copy number values was used to determine the relative population (%) of RH and BA in the cocultures. The optical density (OD) was determined at 600nm. The amount of glucose and SCFAs was determined by HPLC.

Figure S3

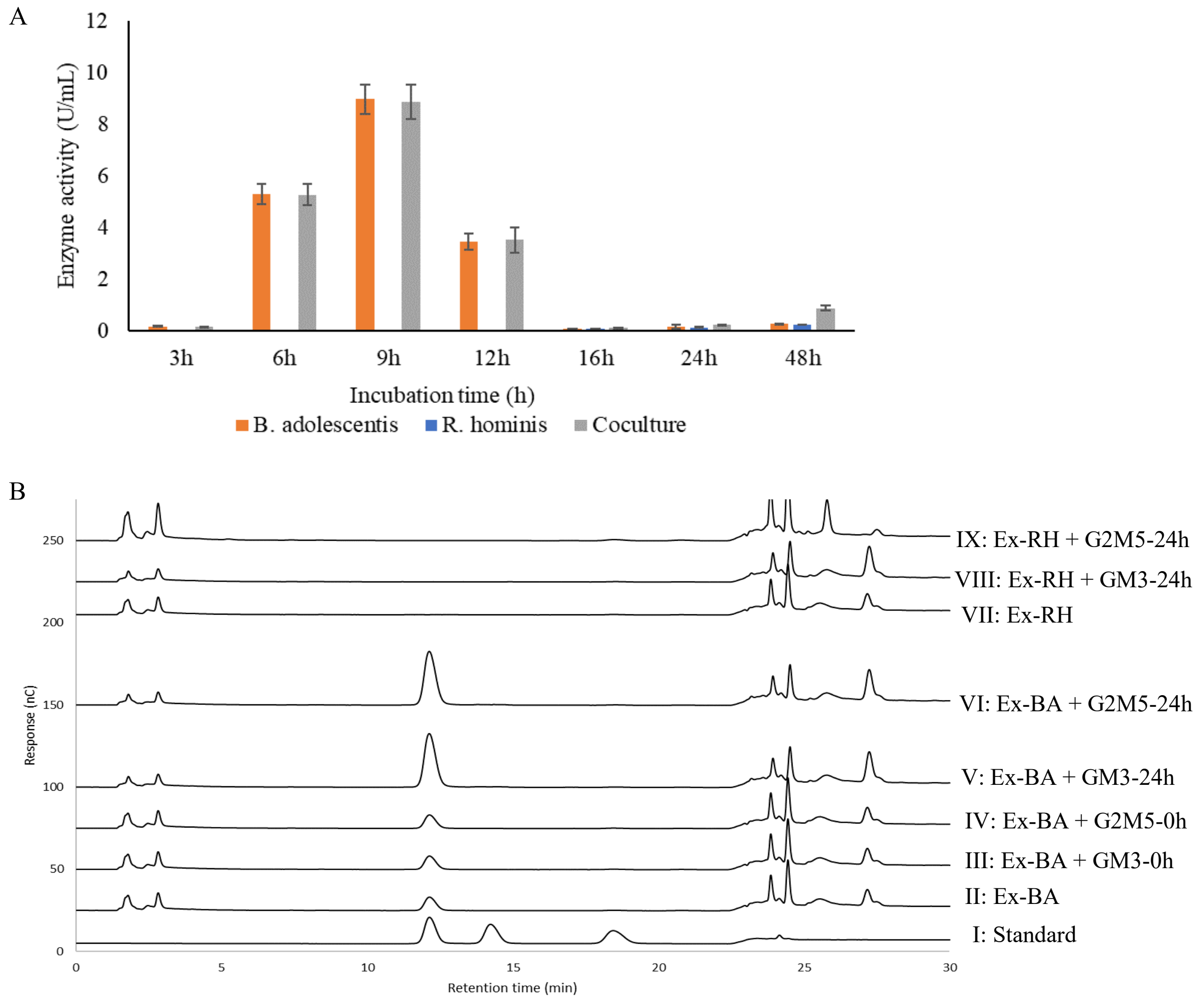

Figure S3. Determining the extracellular (Ex) α-galactosidase activity in *B. adolescentis* (BA) and *R. hominis* (RH). (A) α-galactosidase activity assay. (B) Galactose release estimation by HPAEC-PAD analysis.

A. The activity assay was carried out by incubation of the extracellular fraction (Ex) (9 h) of *B. adolescentis* and *R. hominis* and coculture with *p*NP-α-galactopyranoside (1 mM) for 10 min at 37 ºC.

B. The extracellular fraction (Ex) (9 h) of *B. adolescentis* (BA) and *R. hominis* A2-183 (RH) was incubated separately with either galactosyl-mannotriose (GM3) or di-galactosyl-mannopentaose (G2M5) at 2.5 mM for 24 h at 37ºC. The galactose release was measured by HPAEC-PAD using the CarboPac PA20 column as mentioned in Materials and Methods under subsection carbohydrate analysis.

Samples: I, Standards, II, the extracellular fraction of *B. adolescentis*; III, the extracellular fraction of *B. adolescentis* + GM3-0h; IV, the extracellular fraction of *B. adolescentis* + G2M5-0h; V, the extracellular fraction of *B. adolescentis* + GM3-24h; VI, the extracellular fraction of *B. adolescentis* + G2M5-24h; VII, the extracellular fraction of *R. hominis*; VIII, the extracellular fraction of *R. hominis* + GM3; IX, the extracellular fraction of *R. hominis* + G2M5.

Figure S4.

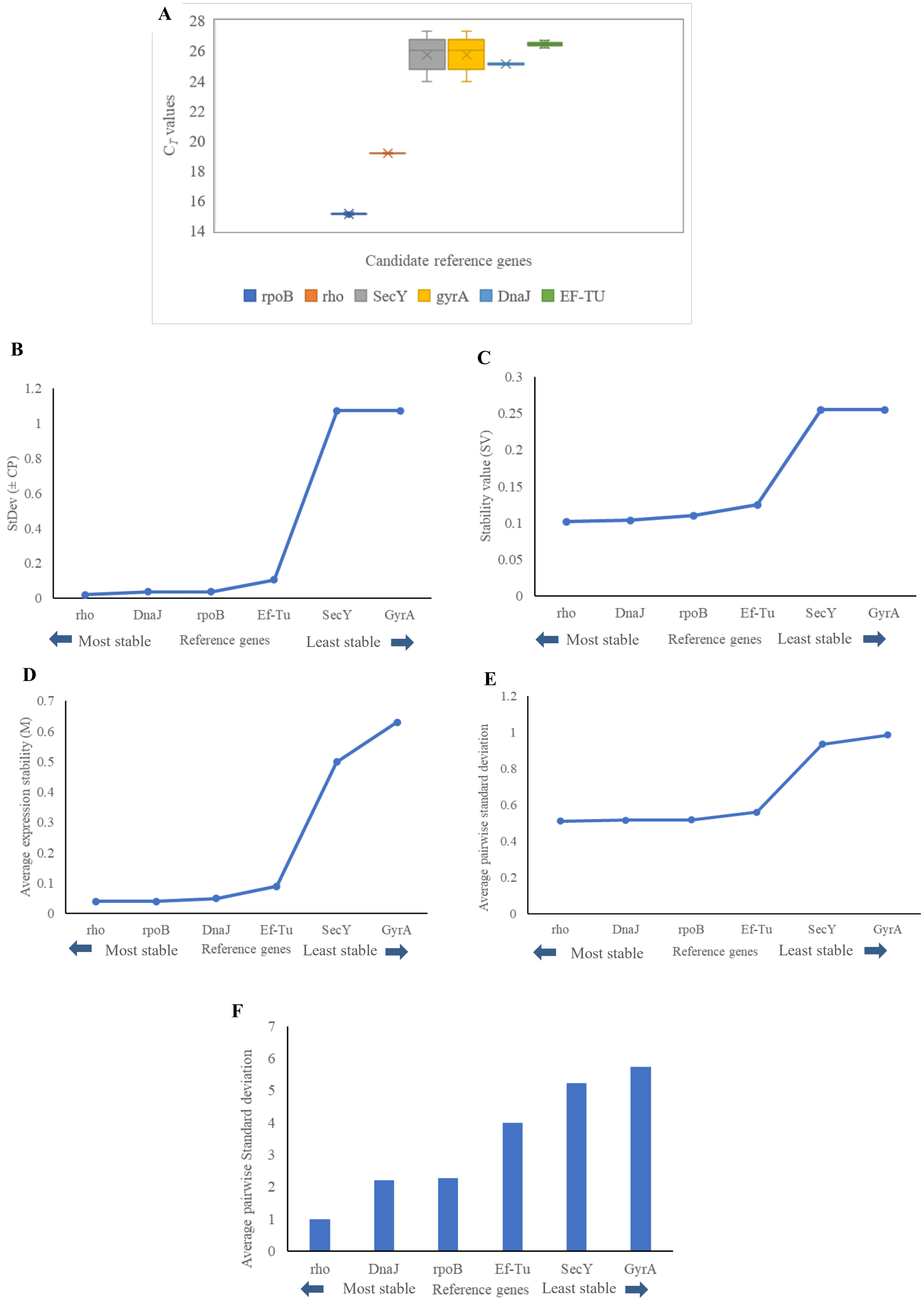
A
28
26
24
22
20
18
16
14
C_T values
Candidate reference genes
rpoB
rho
SecY
gyrA
DnaJ
EF-TU
B
StDev (± CP)
1.2
1
0.8
0.6
0.4
0.2
0
rho
DnaJ
rpoB
Ef-Tu
SecY
GyrA
Most stable
Reference genes
Least stable
C
Stability value (SV)
0.3
0.25
0.2
0.15
0.1
0.05
0
rho
DnaJ
rpoB
Ef-Tu
SecY
GyrA
Most stable
Reference genes
Least stable
D
Average expression stability (M)
0.7
0.6
0.5
0.4
0.3
0.2
0.1
0
rho
rpoB
DnaJ
Ef-Tu
SecY
GyrA
Most stable
Reference genes
Least stable
E
Average pairwise standard deviation
1.2
1
0.8
0.6
0.4
0.2
0
rho
DnaJ
rpoB
Ef-Tu
SecY
GyrA
Most stable
Reference genes
Least stable
F
Average pairwise Standard deviation
7
6
5
4
3
2
1
0
rho
DnaJ
rpoB
Ef-Tu
SecY
GyrA
Most stable
Reference genes
Least stable

Figure S4. Expression of reference genes and analysis of their stability in *R. hominis*. (A) Gross expression data as C_T values for the candidate reference genes during cultivation of *R. hominis* on four different substrates (glucose, galactose, mannose and MOS/GMOS) and two different time points (9 and 12h). The data is represented as box and whisker plot where the line across the box depicts the median. The box indicates the 25th and 75th percentiles. Bars represent the maximum and minimum values.

The selection of the stable reference gene(s) was carried out by focusing on four different methods: **(B)** BestKeeper software, which ranks the genes in agreement with the standard deviation of their C_T values in correlation with intragroup alterations, (**C**) NormFinder software, which evaluates gene stability using both intragroup and intergroup changes, **(D)** geNorm software, which calculates the stability of each gene through intragroup differences and mean pairwise variation, **(E)** ΔC_T method, which values the fluctuation of the ΔC_T making a comparison between two or more reference genes.

The data were integrated to obtain a final rank, based on the geometric mean, using the **(F)** RefFinder tool.

Figure S5

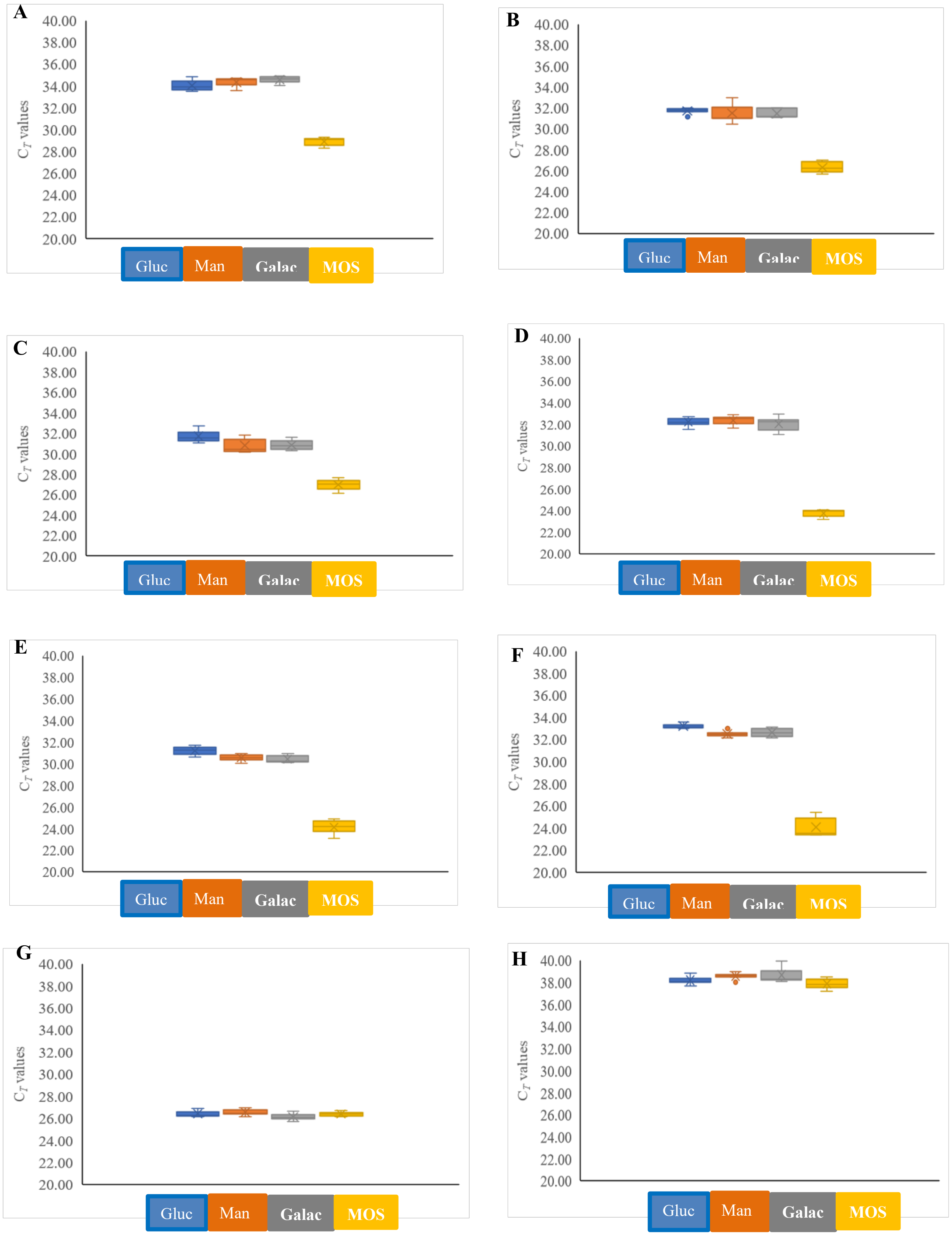
A
B
C
D
E
F
G
H
40.00
38.00
36.00
34.00
32.00
30.00
28.00
26.00
24.00
22.00
20.00
C_T values
Gluc
Man
Galac
MOS

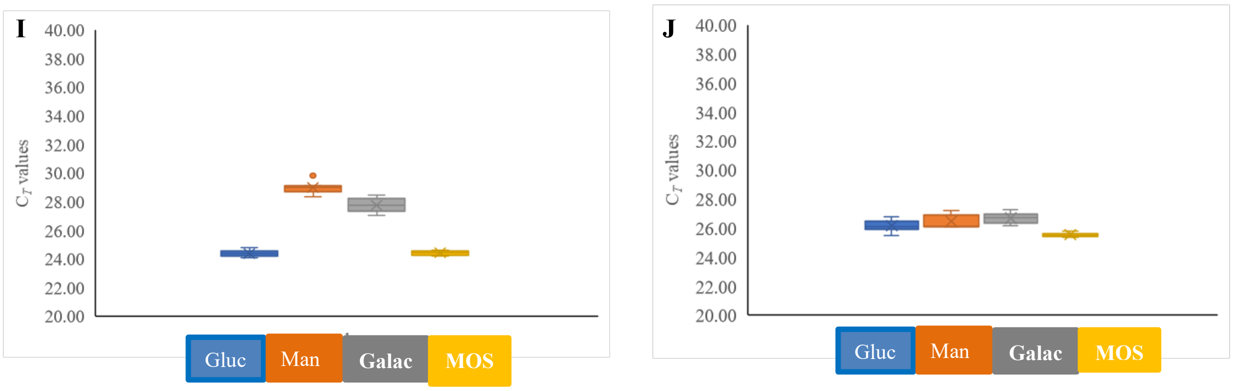

Figure S5. **Expression data represented as cycle threshold (C_T) values determined using RT-qPCR for analysis of target genes during cultivation of *R. hominis* after 9 h of incubation.** The expression of target genes (putative gene function and GH family mentioned in bracket) were analysed during cultivation on four different substrates,glucose (Glu), mannose (Man), galactose (Galac) and MOS/GMOS (MOS), after 9 h of incubation. Target genes: (**A**) RHOM_RS11135 (β-mannoside phosphorylase, GH130A); (**B**) RHOM_RS11140 (4-O-β-D-mannosyl-D-glucose phosphorylase, GH130); (**C**) RHOM_RS11145 (mannobiose 2-epimerase); (**D**) RHOM_RS11160 (ABC Substrate-binding protein); (**E**), RHOM_RS11175 (α-galactosidase, GH 36A); (**F**) RHOM_RS14160 (β-mannoside hydrolase, GH 113A); (**G**) RHOM_RS06295 (α-galactosidase, GH36B); (**H**) RHOM_RS05895 (α-galactosidase, GH 27); (**I**) RHOM_RS13400 (Butyl CoA: acetate CoA transferase); (**J**) RHOM_RS15885 (Lactate dehydrogenase)**.** The data are represented as a box and whisker plot where the line across the box depicts the median. The box indicates the 25th and 75th percentiles. Bars represent the maximum and minimum values. All experiments were carried out with three biological triplicates along with a technical duplicate of each biological replicate.

Figure S6

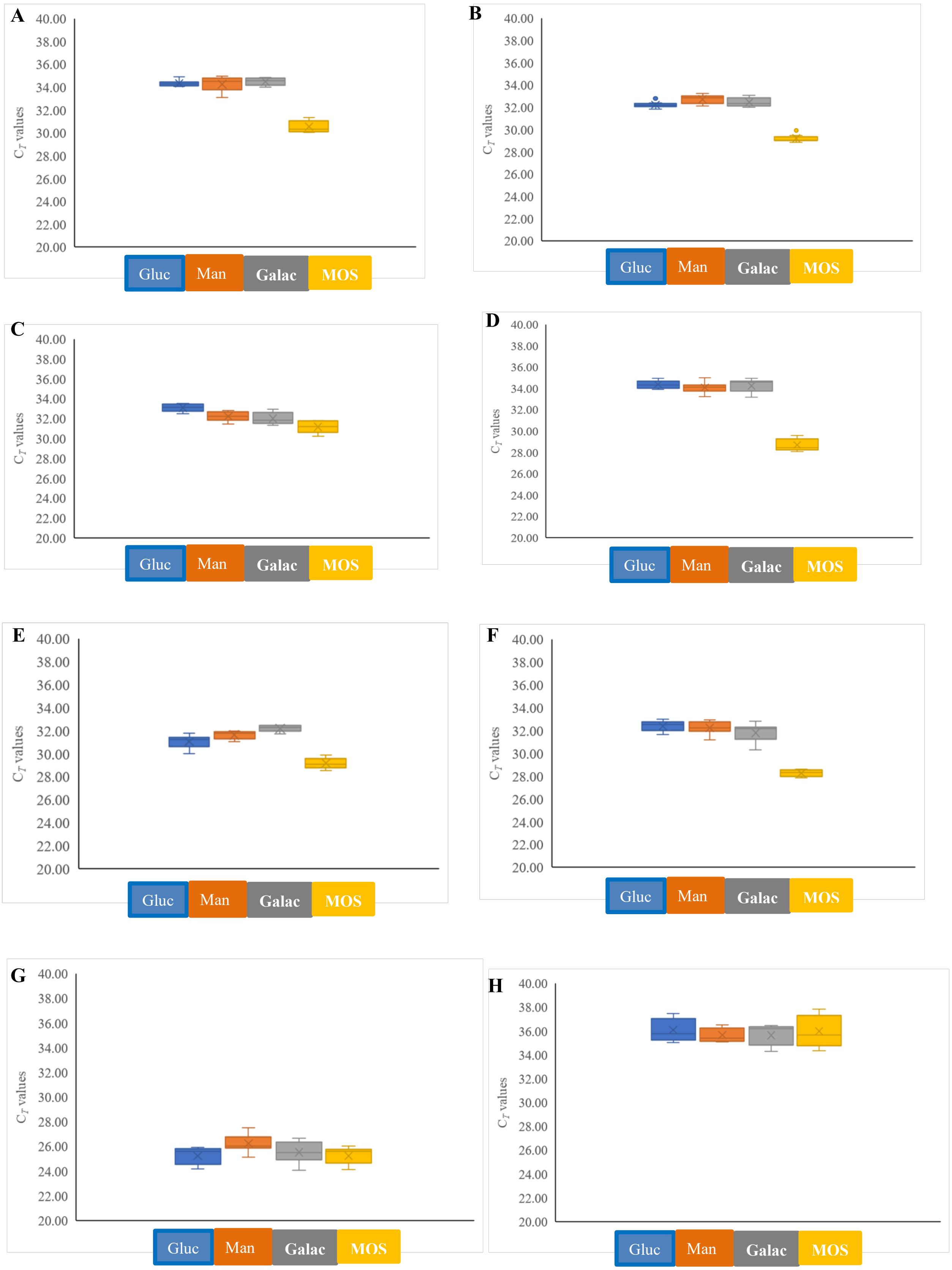
A
B
C
D
E
F
G
H
40.00
38.00
36.00
34.00
32.00
30.00
28.00
26.00
24.00
22.00
20.00
C_T values
Gluc
Man
Galac
MOS

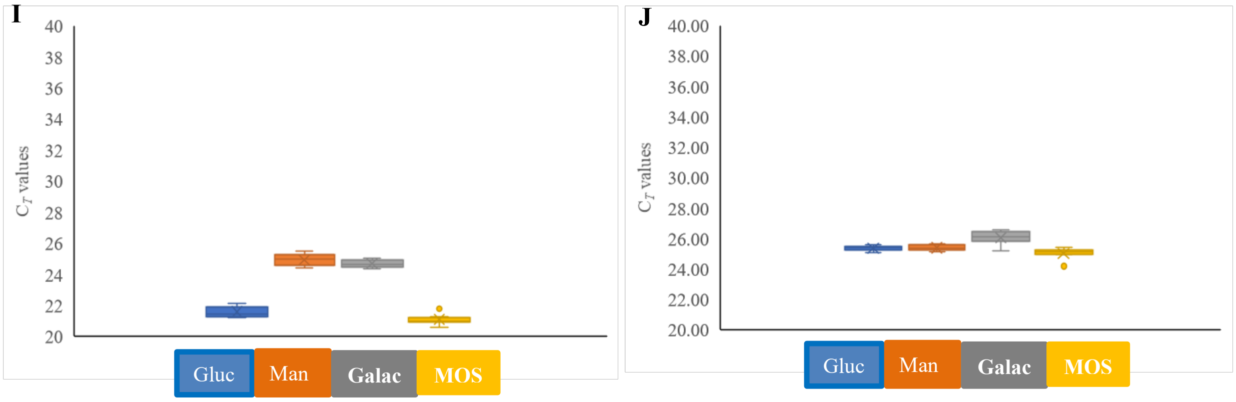

Figure S6. **Expression data represented as cycle threshold (C_T) values determined using RT-qPCR for analysis of target genes during cultivation of *R. hominis* after 12 h of incubation.** The expression of target genes (putative gene functions and GH family mentioned in bracket) were analysed during cultivation on four different substrates,glucose (Glu), mannose (Man), galactose (Galac) and MOS/GMOS (MOS), after 12 h of incubation. Target genes: (**A**) RHOM_RS11135 (β- mannoside phosphorylase, GH130A); (**B**) RHOM_RS11140 (4-O-β-D-mannosyl-D-glucose phosphorylase, GH130); (**C**) RHOM_RS11145 (mannobiose 2-epimerase); (**D**) RHOM_RS11160 (ABC Substrate-binding protein); (**E**), RHOM_RS11175 (α-galactosidase, GH36A); (**F**) RHOM_RS14160 (β-mannanase, GH113A); (**G**) RHOM_RS06295 (α-galactosidase, GH36B); (**H**) RHOM_RS05895 (α-galactosidase, GH27); (**I**); RHOM_RS13400 (Butyl CoA: acetate CoA transferase); (**J**) RHOM_RS15885 (Lactate dehydrogenase)**.** The data are represented as a box and whisker plot where the line across the box depicts the median. The box indicates the 25th and 75th percentiles. Bars represent the maximum and minimum values. All experiments were carried out with three biological triplicates along with a technical duplicate of each biological replicate.

Table S4. Top 3 BLASTp results for selected MOS/GMOS utilisation locus, *Rh*MosUL, encoded protein sequences against the Protein Data Base (PDB).

***Rh*MOP130A**							
Protein Name:	Seq ID %	Query Cover %	AA length:	Function:	Refseq ID:	PDB ID:	GH family
tm1225	61.54%	95%	338	Beta-mannoside phosphorylase	N.A	1VKD	GH130
Uhgb_MP	54.06%	93%	347	Mannoside phosphorylase	N.A	4UDK	GH130
RaMP2	53.66%	94%	335	Mannooligosaccharide (Mos) phosphorylase	WP_013496855.1	5AYD	GH130
***Rh*Man113A**							
AxMan113A	50.98%	96%	309	Beta-1,4-mannanase	WP_015010951.1	5YLH	GH113
*Ba*Man113A	43.31%	98%	348	Endo-beta-1,4-mannanase	N.A	7DV7	GH113
*Aa*ManA	40.26%	95%	343	Endo-beta-1,4-mannanase	N.A	3CIV	GH113
***Rh*Gal36A**							
*Aga*B	49.45%	99%	729	Alpha-galactosidase	(genbank)AAG49421.1	4FNQ	GH36
*Aga*A	49.66%	99%	729	Alpha-galactosidase	(genbank) AAG49420.1	4FNR	GH36
*Aga*SK	45.48%	99%	720	Alpha-galactosidase domain	N.A	2YFN	GH36

Rh*MosBP							
*Bl*MnBP1	56.74%	88%	444	Mos binding (of ABC transporter)	N.A	6I5R	Not defined
*Bl*MnBP2	55.82%	86%	427	Mos binding (of ABC transporter)	N.A	6FUV	Not defined

*Only two homologous sequences of *Rh*MosBP fulfilling the selection criteria were identified in the PDB database. Sequence identities (Seq ID%) higher than 30% are presented. Query Coverage (Query Cover %) refers to query sequence overlap with the reference sequence. AA length refers to number of amino acids. Refseq, Genbank, and PDB entry numbers and -glycoside hydrolase (GH) family (for the enzymes according to CAZy database) are indicated if available

Figure S7

A)

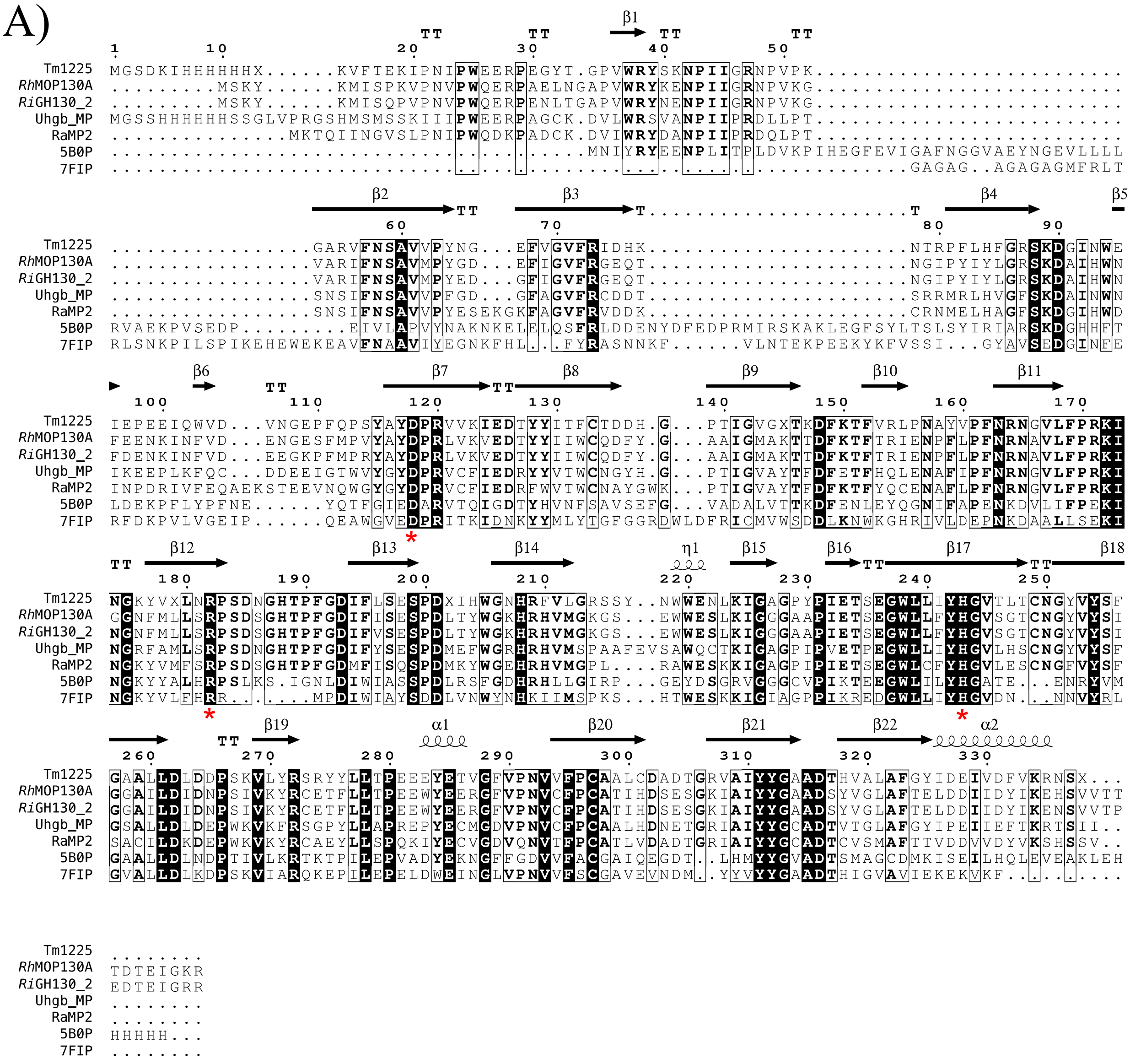

Tm1225 MGSDKIHHHHHHX......KVFTEKIPNIPWEERPEGYT.GPVWRYSKNPIIGRNPVPK
RhMOP130A ....MSKY......KMISPKVPNVPWQERPAELNGAPVWRYKENPIIGRNPVKG
RiGH130_2 ....MSKY......KMISQPVPNVPWQERPENLTGAPVWRYNENPIIGRNPVKG
Uhgb_MP MGSSHHHHHHSSGLVPRGSHMSMSSKIIIPWEERPAGCK.DVLWRSVANPIIPRDLLPT
RaMP2 ..............MKTQIINGVSLPNIPWQDKPADCK.DVIWRYDANPIIPRDQLPT
5B0P MNIYRYEENPLITPLDVKPIHEGFEVIGAFNGGVAEYNGEVLLLL
7FIP GAGAG..AGAGAGMFRLT
Tm1225 GARVFNSAVVPYNG...EFVGVFRIDHK NTRPFLHFGRSKDGINWE
RhMOP130A VARIFNSAVMPYGD...EFIGVFRGEQT NGIPYIYLGRSKDAIHWN
RiGH130_2 VARIFNSAVMPYED...GFIGVFRGEQT NGIPYIYLGRSKDAIHWN
Uhgb_MP SNSIFNSAVVPFGD...GFAGVFRCDDT SRRMRLHVGFSKDAINWN
RaMP2 SNSIFNSAVVPYESEKGKFAGVFRVDDK CRNMELHAGFSKDGIHWD
5B0P RVAEKPVSEDP.........EIVLAPVYNAKNKELELQSFRLDDENYDFEDPRMIRSKAKLEGFSYLTSLSYIRIARSKDGHHFT
7FIP RLSNKPILSPIKEHEWEKEAVFNAAVIYEGNKFHL..FYRASNNKF.....VLNTEKPEEKYKFVSSI..GYAVSEDGINFE
Tm1225 IEPEEIQWVD...VNGEPFQPSYAYDPRVVKIEDTYYITFCTDDH.G...PTIGVGXTKDFKTFVRLPNAYVPFNRNGVLFPRKI
RhMOP130A FEENKINFVD...ENGESFMPVYAYDPRLVKVEDTYYIIWCQDFY.G...AAIGMAKTTDFKTFTRIENPFLPFNRNAVLFPRKI
RiGH130_2 FDENKINFVD...EEGKPFMPRYAYDPRLVKVEDTYYIIWCQDFY.G...AAIGMAKTTDFKTFTRIENPFLPFNRNAVLFPRKI
Uhgb_MP IKEEPLKFQC...DDEEIGTWVYGYDPRVCFIEDRYYVTWCNGYH.G...PTIGVAYTFDFETFHQLENAFIPFNRNGVLFPRKI
RaMP2 INPDRIVFEQAEKSTEEVNQWGYGYDPRVCFIEDRFWVTWCNAYGWK...PTIGVAYTFDFKTFYQCENAFLPFNRNGVLFPRKI
5B0P LDEKPFLYPFNE......YQTFGIEDARVTQIGDTYHVNFSAVSEFG...VADALVTTKDFENLEYQGNIFAPENKDVLIFPEKI
7FIP RFDKPVLVGEIP......QEAWGVEDPRITKIDNKYYMLYTGFGGRDWLDFRICMVWSDDLKNWKGHRIVLDEPNKDAALLSEKI
Tm1225 NGKYVXLNRPSDNGHTPFGDIFLSESPDXIHWGNHRFVLGRSSY..NWWENLKIGAGPYPIETSEGWLLIYHGVTLTCNGYVYSF
RhMOP130A GGNFMLLSRPSDSGHTPFGDIFISESPDLTYWGKHRHVMGKGS...EWWESLKIGGGAAPIETSEGWLLFYHGVSGTCNGYVYSI
RiGH130_2 NGNFMLLSRPSDSGHTPFGDIFVSESPDLTYWGKHRHVMGKGS...EWWESLKIGGGAAPIETSEGWLLFYHGVSGTCNGYVYSI
Uhgb_MP NGRFAMLSRPSDNGHTPFGDIFYSESPDMEFWGRHRHVMSPAAFEVSAWQCTKIGAGPIPVETPEGWLLIYHGVLHSCNGYVYSF
RaMP2 NGKYVMFSRPSDSGHTPFGDMFISQSPDMKYWGEHRHVMGPL....RAWESKKIGAGPIPIETSEGWLCFYHGVLESCNGFVYSF
5B0P NGKYYALHRPSLKS.IGNLDIWIASSPDLRSFGDHRHLLGIRP...GEYDSGRVGGGCVPIKTEEGWLILYHGATE..ENRYVM
7FIP NGKYVLFHRR.......MPDIWIAYSDDLVNWYNHKIIMSPKS...HTWESKKIGIAGPPIKRDEGWLLIYHGVDN...NNVYRL
Tm1225 GAALLDLDDPSKVLYRSRYYLLTPEEEYETVGFVPNVVFPCAALCDADTGRVAIYYGAADTHVALAFGYIDEIVDFVKRNSX...
RhMOP130A GGAILDIDNPSIVKYRCETFLLTPEEWYEERGFVPNVCFPCATIHDSESGKIAIYYGAADSYVGLAFTELDDIIDYIKEHSVVTT
RiGH130_2 GGAILDIDNPSIVKYRCETFLLTPEEWYEERGFVPNVCFPCATIHDSESGKIAIYYGAADSYVGLAFTELDDIIDYIKENSVVTP
Uhgb_MP GSALLDLDEPWKVKFRSGPYLLAPREPYECMGDVPNVCFPCAALHDNETGRIAIYYGCADTVTGLAFGYIPEIIEFTKRTSII..
RaMP2 SACILDKDEPWKVKYRCAEYLLSPQKIYECVGDVQNVTFPCATLVDADTGRIAIYYGCADTCVSMAFTTVDDVVDYVKSHSSV..
5B0P GAALLDLNDPTIVLKRTKTPILEPVADYEKNGFFGDVVFACGAIQEGDT..LHMYYGVADTSMAGCDMKISEILHQLEVEAKLEH
7FIP GVALLDLKDPSKVIARQKEPILEPELDWEINGLVPNVVFSCGAVEVNDM..YVYYGAADTHIGVAVIEKEKVKF
Tm1225 ....
RhMOP130A TDTEIGKR
RiGH130_2 EDTEIGRR
Uhgb_MP ....
RaMP2 ....
5B0P HHHHH...
7FIP ....

B)

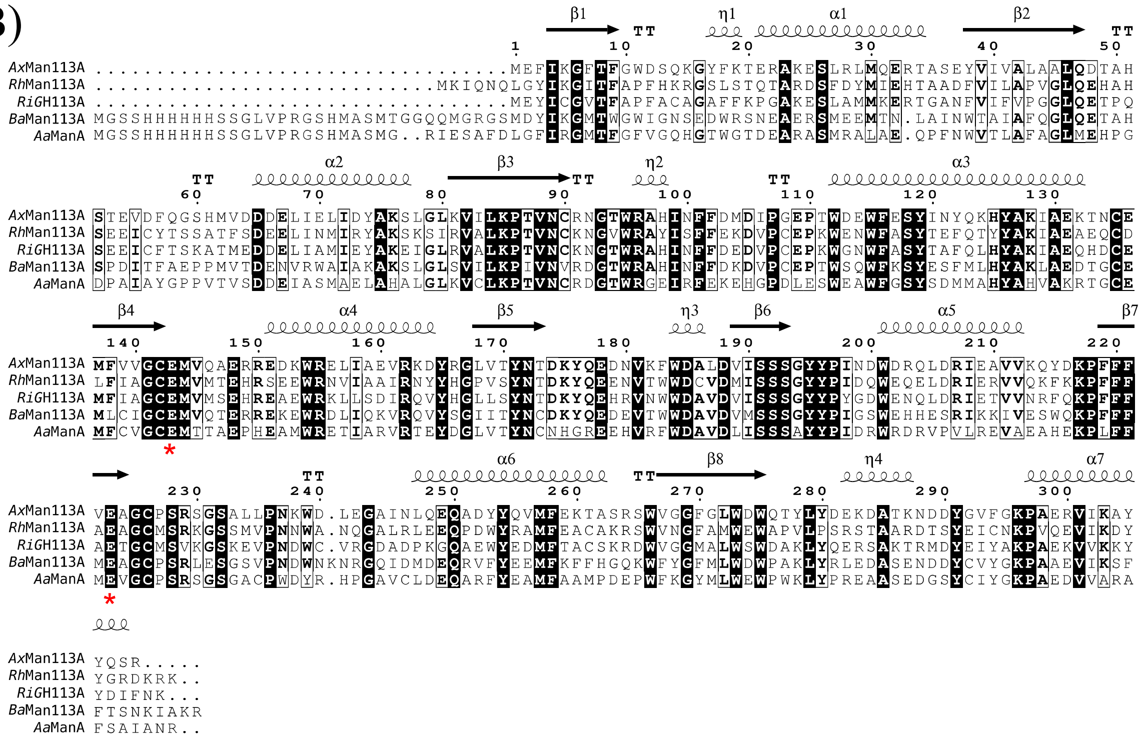
AxMan113A ..............................MEFIKGFTFGWDSQKGYFKTERAKESLRLMQERTASEYVIVALAALQDTAH
RhMan113A ..........................MKIQNQLGYIKGITFAPFHKRGSLSTQTARDSFDYMIEHTAADFVILAPVGLQEHAH
RiGH113A ..............................MEYICGVTFAPFACAGAFFKPGAKESLAMMKERTGANFVIFVPGGLQETPQ
BaMan113A MGSSHHHHHHSSGLVPRGSHMASMTGGQQMGRGSMDYIKGMTWGWIGNSEDWRSNEAERSMEEMTN.LAINWTAIAFQGLQETAH
AaManA MGSSHHHHHHSSGLVPRGSHMASMG..RIESAFDLGFIRGMTFGFVGQHGTWGTDEARASMRALAE.QPFNWVTLAFAGLMEHPG
AxMan113A STEVDFQGSHMVDDDELIELIDYAKSLGLKVILKPTVNCRNGTWRAHINFFDMDIPGEPTWDEWFESYINYQKHYAKIAEKTNCE
RhMan113A SEEICYTSSATFSDEELINMIRYAKSKSIRVALKPTVNCKNGVWRAYISFFEKDVPCEPKWENWFASYTEFQTYYAKIAEAEQCD
RiGH113A SEEICFTSKATMEDDELIAMIEYAKEIGLRVALKPTVNCKNGTWRAHINFFDEDVPCEPKWGNWFASYTAFQLHYAKIAEQHDCE
BaMan113A SPDITFAEPPMVTDENVRWAIAKAKSLGLSVILKPIVNVRDGTWRAHINFFDKDVPCEPTWSQWFKSYESFMLHYAKLAEDTGCE
AaManA DPAIAYGPPVTVSDDEIASMAELAHALGLKVCLKPTVNCRDGTWRGEIRFEKEHGPDLESWEAWFGSYSDMMAHYAHVAKRTGCE
AxMan113A MFVVGCEMVQAERREDKWRELIAEVRKDYRGLVTYNTDKYQEDNVKFWDALDVISSSGYYPINDWDRQLDRIEAVVKQYDKPFFF
RhMan113A LFIAGCEMVMTEHRSEEWRNVIAAIRNYYHGPVSYNTDKYQEENVTWWDCVDMISSSGYYPIDQWEQELDRIERVVQKFKKPFFF
RiGH113A MFIAGCEMVMSEHREAEWRKLLSDIRQVYHGLLSYNTDKYQEHRVNWWDAVDVISSSGYYPYGDWENQLDRIETVVNRFQKPFFF
BaMan113A MLCIGCEMVQTERREKEWRDLIQKVRQVYSGIITYNCDKYQEDEVTWWDAVDVMSSSGYYPIGSWEHHESRIKKIVESWQKPFFF
AaManA MFCVGCEMTTAEPHEAMWRETIARVRTEYDGLVTYNCNHGREEHVRFWDAVDLISSSAYYPIDRWRDRVPVLREVAEAHEKPLFF
AxMan113A VEAGCPSRSGSALLPNKWD.LEGAINLQEQADYYQVMFEKTASRSWVGGFGLWDWQTYLYDEKDATKNDDYGVFGKPAERVIKAY
RhMan113A AEAGCMSRKGSSMVPNNWA.NQGALRLEEQPDWYRAMFEACAKRSWVNGFAMWEWAPVLPSRSTAARDTSYEICNKPVQEVIKDY
RiGH113A AETGCMSVKGSKEVPNDWC.VRGDADPKGQAEWYEDMFTACSKRDWVGGMALWSWDAKLYQERSAKTRMDYEIYAKPAEKVVKKY
BaMan113A MEAGCPSRLESGSVPNDWNKNRGQIDMDEQRVFYEEMFKFFHGQKWFYGFMLWDWPAKLYRLEDASENDDYCVYGKPAAEVIKSF
AaManA MEVGCPSRSGSGACPWDYR.HPGAVCLDEQARFYEAMFAAMPDEPWFKGYMLWEWPWKLYPREAASEDGSYCIYGKPAEDVVARA
AxMan113A YQSR.....
RhMan113A YGRDKRK..
RiGH113A YDIFNK...
BaMan113A FTSNKIAKR
AaManA FSAIANR..

C)

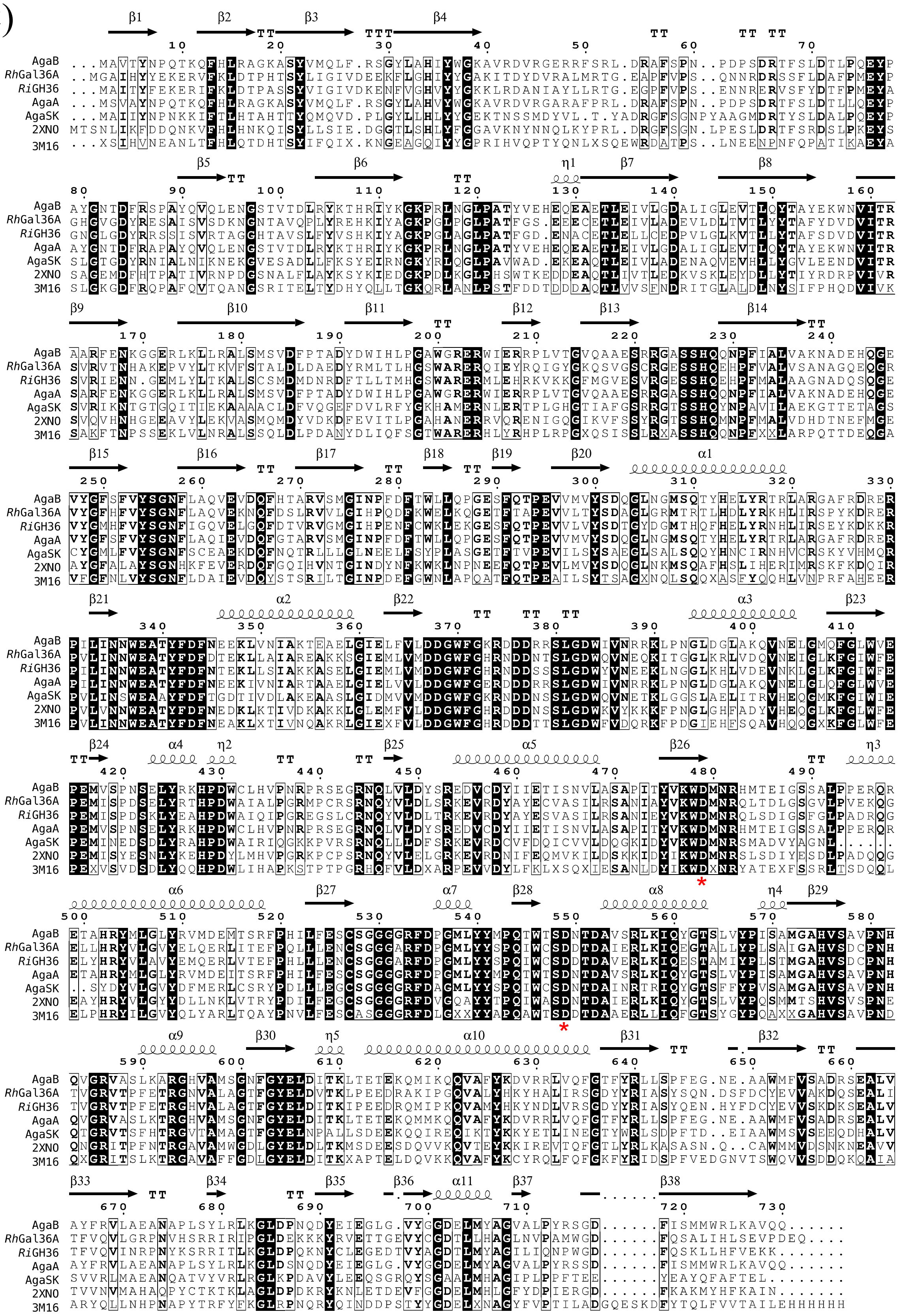

D)

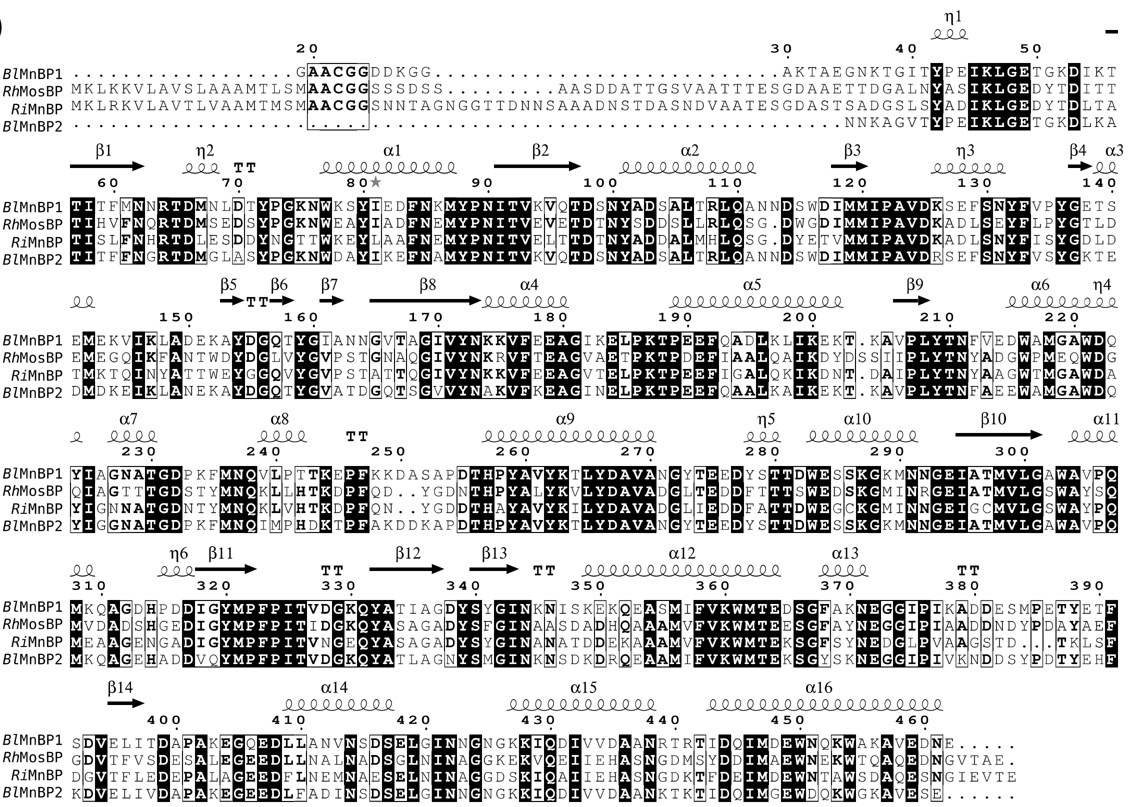

Figure S7. Multiple Sequence Alignment (MSA) with selected *R. hominis* protein sequences, *R. intestinalis* homologs and proteins obtained by the BLASTp search of the PDB database as mentioned in Table S4. Sequence alignment with Clustal Omega (https://www.ebi.ac.uk/). Conserved residues are highlighted in black (important functions marked with a star), and semiconserved (>50%) are boxed in black with conservations in bold. The order of sequences is: top hit in BLASTp, query sequence (*R. hominis* protein), *R. intestinalis* homolog, and remaining BLAST hits in order of highest identity. Amino acid numbering and secondary structure is indicated at the top of the sequence. **A**: MSA for *Rh*MOP130A: catalytic residue (D104) and phosphate binding residues in Uhgb_MP (R174 and H234) (pdb: 4UDK, Ladevése et al., 2013, Li et al 2022) are conserved in the sequences (D110, R174. H234 in *Rh*MOP130A and D118, R182, H243 in tm1225). **B**: MSA for *Rh*Man113A: catalytic residues E143 and E223 in *Ax*Man113A (pdb: 5YLH, You et al 2018) are conserved in the sequences (E149, E229 in *Rh*Man113A). **C**: MSA for *Rh*Gal36A: catalytic residues D479 and D549 in AgaB (pdb: 4FNQ, Merceron et al., 2012) are conserved in the sequences (D479 and D549 in *Rh*Gal36A). **D**: MSA for *Rh*MosBP: several key residues for mannan binding in *Bl*MnBP1 (pdb: 6I5R, Ejby et al., 2019) are conserved. All three residues that contribute to hydrophobic stacking interactions with mannotriose (W216, W303, and Y339 in *Bl*MnBP1 corresponds to W235, W320, and Y356 in *Rh*MosBP) and four (W283, E284, Q307, D338) out of the nine interacting polar residues are conserved.

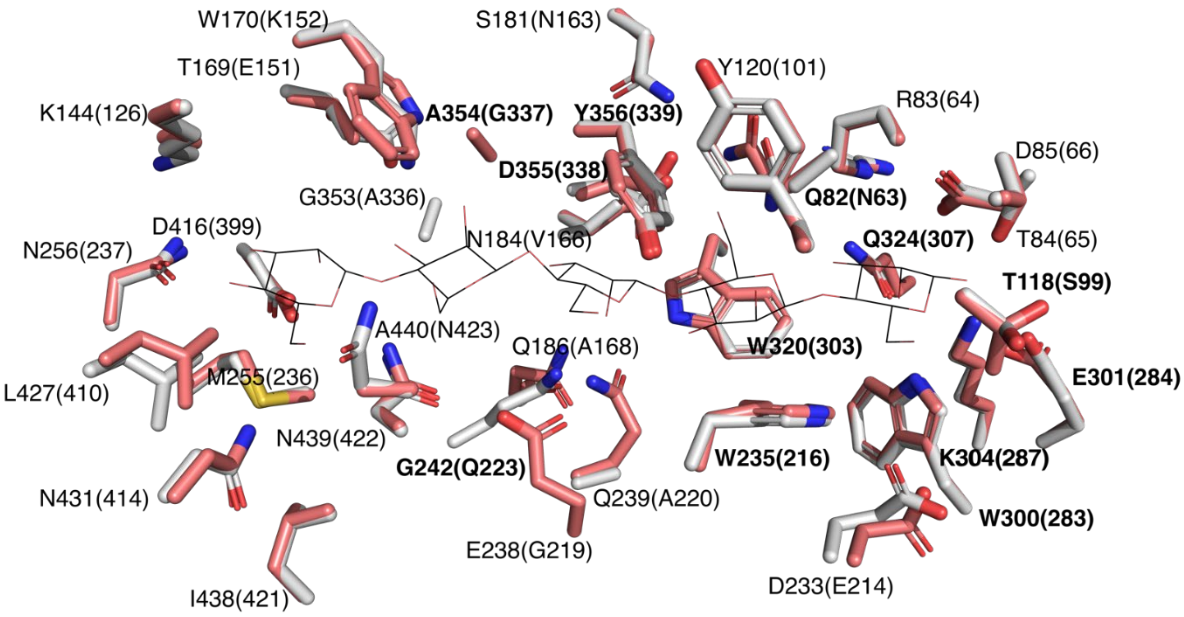

Figure S8. Predicted mannan-oligosaccharide binding site of homology model generated for *Rh*MosBP using *Bl*MnBP1 (pdb: 6I5R) as a template. The homology model of *Rh*MosBP (orange) is superimposed with the crystal structure (pdb: 6I5W) of *Bl*MnBP1 (grey) in complex with manno-pentaose (displayed with thin black lines). Side chains from either structure within 4Å of the ligand are presented. The residues are labelled with the residue in *Rh*MosBP followed by the corresponding residue in *Bl*MnBP1 in parenthesis. Residues described as to contribute to substrate binding for mannotriose in *Bl*MnBP1 (Ejby et al 2019) are highlighted in bold. All 3 hydrophobic stacking residues are conserved between the structures (W235, W320, and Y356) as well as 4 (W283, E284, Q307, D338) of 9 residues that provide polar interactions. The remaining 5 residues are not judged to cause steric hindrance in mannan-oligosaccharide binding for *Rh*MosBP and are variable between the MosBPs analysed in the MSA.

A MGMSKYKMISPKVPNVPWQERPAELNGAPVWRYKENPIIGRNPVKGVARIFNSAVMPYGDEFIGVFRGEQTNGIPY
IYLGRSKDAIHWNFEENKINFVDENGESFMPVYAYDPRLVKVEDTYYIIWCQDFYGAAIGMAKTTDFKTFTRIENP
FLPFNRNAVLFPRKIGGNFMLLSRPSDSGHTPFGDIFISESPDLTYWGKHRHVMGKGSEWWESLKIGGGAAPIETS
EGWLLFYHGVSGTCNGYVYSIGGAILDIDNPSIVKYRCETFLLTPEEWYEERGFVPNVCFPCATIHDSESGKIAIY
YGAADSYVGLAFTELDDIIDYIKEHSVVTTTDTEIGKRGGENLYFQGAAAELALVPRGSSAHHHHHHHHHH

B MGMKIQNQLGYIKGITFAPFHKRGSLSTQTARDSFDYMIEHTAADFVILAPVGLQEHAHSEEICYTSSATFSDEEL
INMIRYAKSKSIRVALKPTVNCKNGVWRAYISFFEKDVPCEPKWENWFASYTEFQTYYAKIAEAEQCDLFIAGCEM
VMTEHRSEEWRNVIAAIRNYYHGPVSYNTDKYQEENVTWWDCVDMISSSGYYPIDQWEQELDRIERVVQKFKKPFF
FAEAGCMSRKGSSMVPNNWANQGALRLEEQPDWYRAMFEACAKRSWVNGFAMWEWAPVLPSRSTAARDTSYEICNK
PVQEVIKDYYGRDKRKAAALEHHHHHH

C MGAIHYYEKERVFKLDTPHTSYLIGIVDEEKFLGHIYYGAKITDYDVRALMRTGEAPFVPSQNNRDRSSFLDAFPM
EYPGHGVGDYRESAISVSDKNGNTAVQPLYREHKIYAGKPGLPGLPATFGSEEECETLEIVLADEVLDLTVTLYYT
AFYDVDVITRSVRVTNHAKEPVYLTKVFSTALDLDAEDYRMLTLHGSWARERQIEYRQIGYGKQSVGSCRGESSHQ
EHPFVALVSANAGQEQGRVYGFHFVYSGNFLAQVEKNQFDSLRVVLGIHPQDFKWELKQGETFTAPEVVLTYSDAG
LGRMTRTLHDLYRKHLIRSPYKDRERPVLINNWEATYFDFDTEKLLAIAREAKKSGIEMLVMDDGWFGHRNDDNTS
LGDWQVNEQKITGGLKRLVDQVNEIGLKFGIWFEPEMISPDSELYRTHPDWAIALPGRMPCRSRNQYVLDLSRKEV
RDYAYECVASILRSANIAYVKWDMNRQLTDLGSGVLPVEKQGELLHRYVLGVYELQERLITEFPQLLLENCSGGGA
RFDPGMLYYSPQIWCSDDTDAIERLKIQEGTALLYPLSAIGAHVSDCPNHTVGRVTPFETRGNVALAGTFGYELDV
TKLPEEDRAKIPGQVALYHKYHALIRSGDYYRIASYSQNDSFDCYEVVAKDQSEALITFVQVLGRPNVHSRRIRIP
GLDEKKKYRVETTGEVYCGDTLLHAGLNVPAMWGDFQSALIHLSEVPDEQAAAELALVPRGSSAHHHHHHHHHH

Figure S9. The amino acid sequences of the studied protein constructs, A: *Rh*MOP130A, B: *Rh*Man113A, and C: *Rh*Gal36A. Amino acids added to each respective native sequence have been underlined. A start codon and a glycine residue to adjust the reading frame was added to the sequences of **A** and **B**. The native stop codon was removed from each gene sequence and codons. Plasmid encoded His-tags (with preceding amino acids) were inserted (a His_6-tag for B and a His_{10}-tag for **A** and **C**).

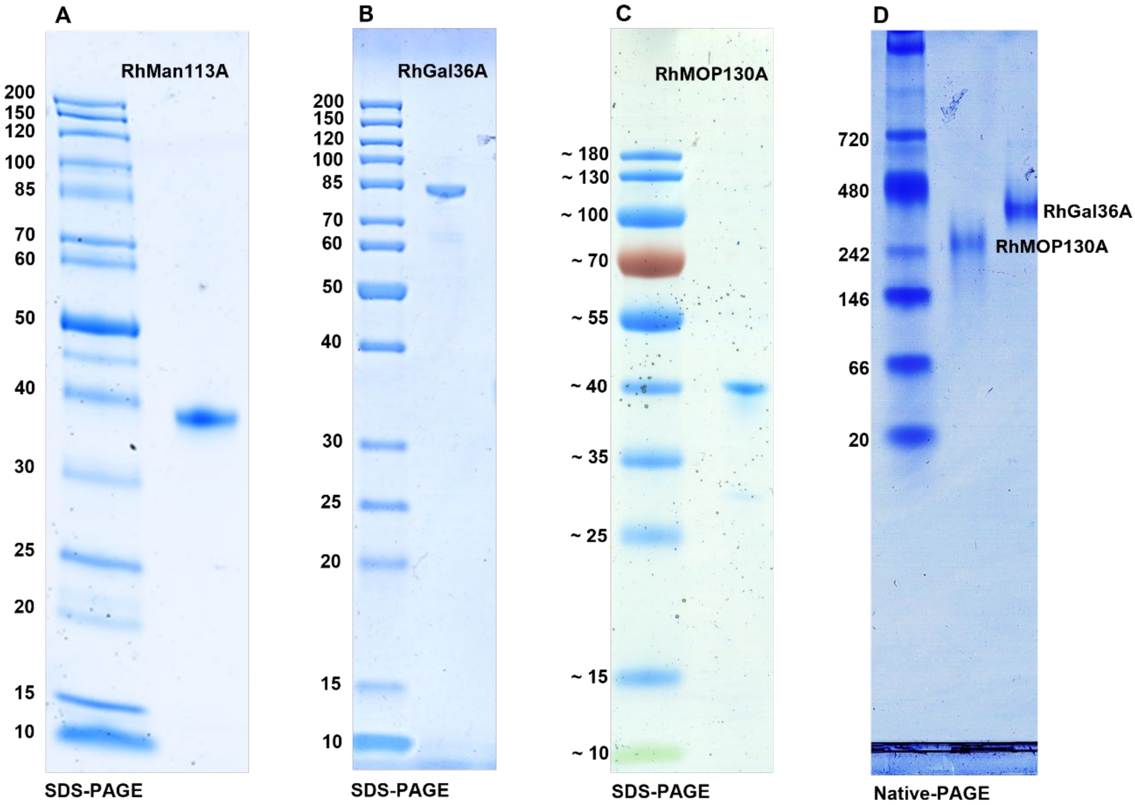

Figure S10. Protein analysis by polyacrylamide gel electrophoresis. Bands of protein ladders have been labelled with their sizes in kDa. The proteins migrated as expected approximately to the theoretical molecular weight of each respective construct (38.6 kDa for *Rh*Man113A, 86.1 for *Rh*Gal36A, and 42.4 kDa for *Rh*MOP130A) **A**: Purified *Rh*Man113A (SDS-PAGE) with a visible a band at 38.3 kDa. **B**: Purified *Rh*Gal36A (SDS-PAGE) with a band at 86.7 kDa. **C**: *Rh*MOP130A (SDS-PAGE) exhibits a band ~41 kDa, and **D**: native-PAGE of purified *Rh*Gal36A and *Rh*MOP130A. The proteins migrated to approximately 346 kDa and 232 kDa in the native-PAGE. Lanes that are not relevant have been cut out from the gel.

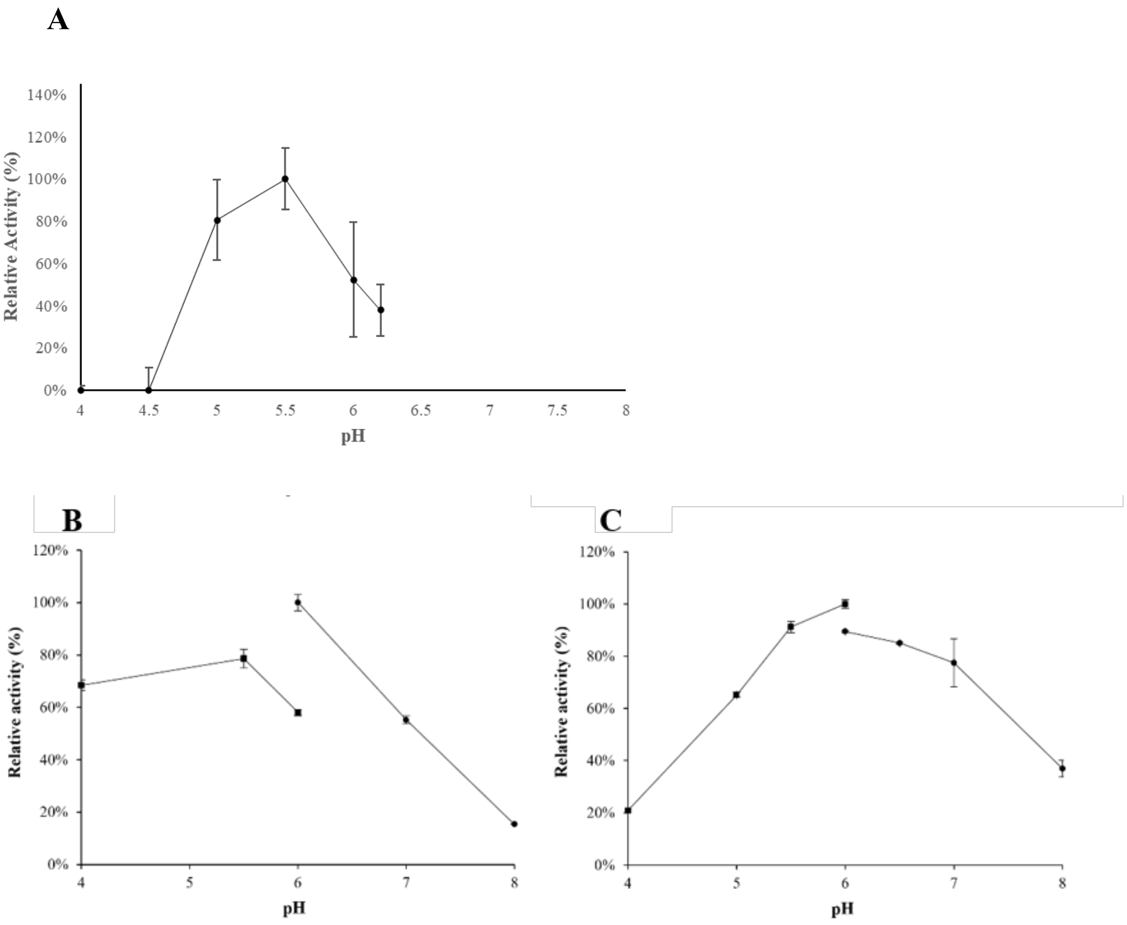

Figure S11. The effect of pH on enzyme activity. A: *Rh*MOP130A (synthesis direction): determined by the phosphate release assay with M_4 after 10 min at 37°C in 50 mM sodium citrate from pH 4 to 6.2. **B**: *Rh*Man113A: determined by measuring the mannose release when incubated with mannotetraose (M_4) for 30 min at 30°C in 50mM sodium citrate buffer (pH 4-6) and sodium phosphate buffer (pH 6-8). **C**: *Rh*Gal36A: determined by pNP-gal hydrolysis after 10 min at 37°C and pH 4-8 in 50mM sodium citrate buffer (pH 4-6) and sodium phosphate buffer (pH 6-8).

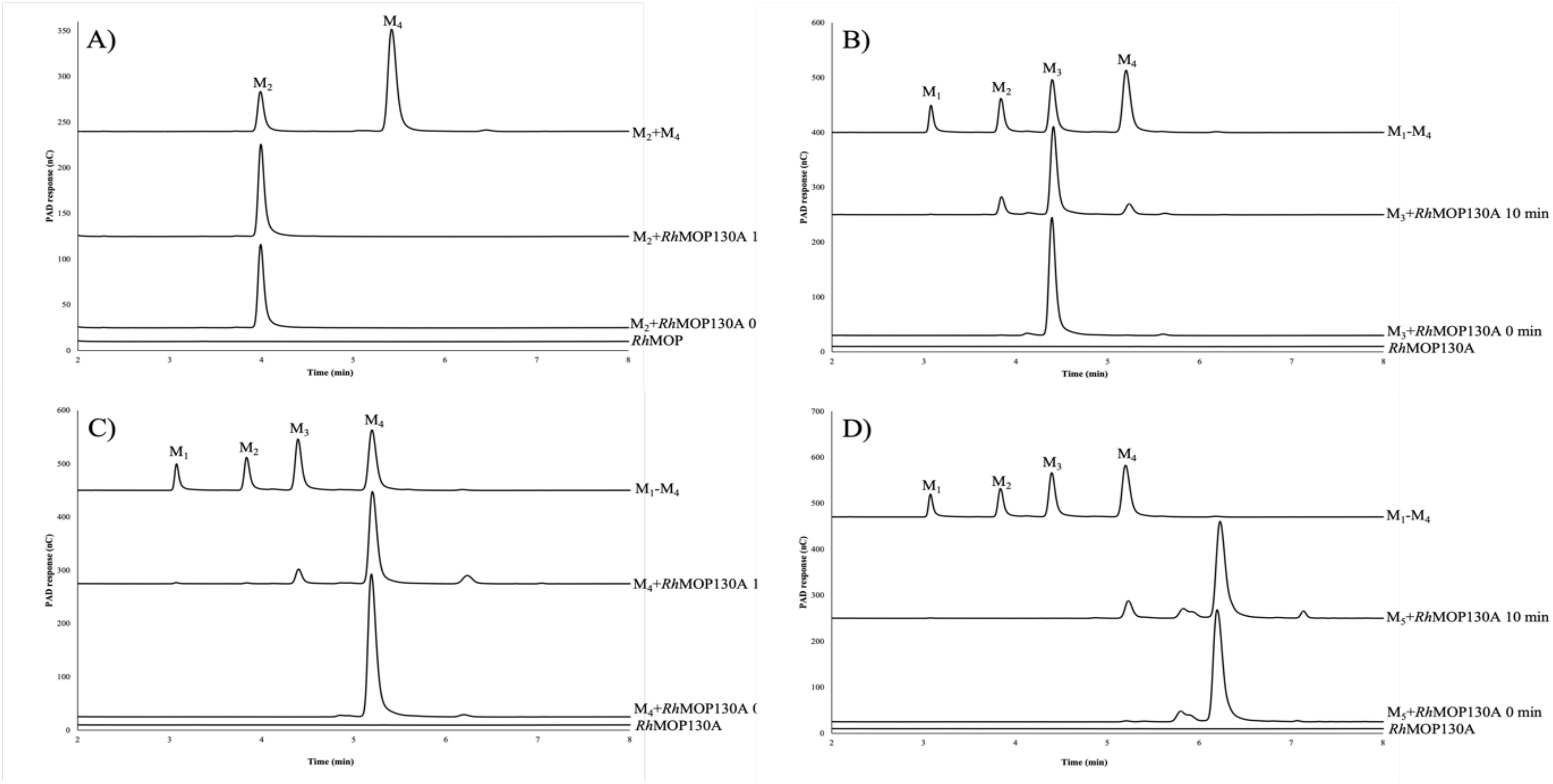

Figure S12. *Rh*MOP130A incubated with different mannan-oligosaccharides analysed by HPAEC. Each incubation consisted of 0.14 mg/ml of *Rh*MOP130A, 10 mM appropriate manno-oligosaccharide (mannobiose, M2, to mannotetraose, M4), and 10 mM phosphate in 100 mM sodium citrate, pH 6.0 for 10 minutes at 37 °C. Top chromatograms: M2 and M4 **(A)**, control injections of M1 (mannose), M2, M3, and M4 (**B, C, D**). Middle chromatograms: *Rh*MOP130A incubated with **A**: 10 mM of M2, **B**: 10 mM of M3, **C**: 10 mM of M4, **D**: 10 mM of M5. Bottom chromatograms: corresponding reactions terminated at 0 min.

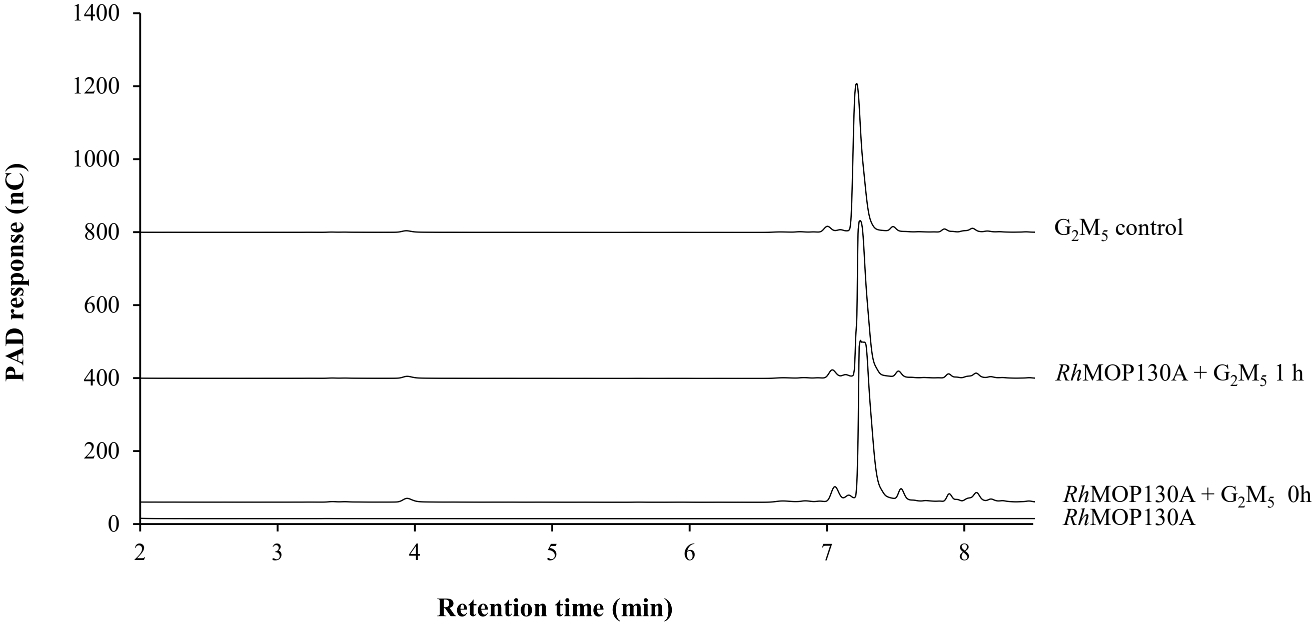

Figure S13. *Rh*MOP130A incubated with G2M5 analysed on a PA200 column using HPAEC-PAD. The mannan-oligosaccharide release of incubations with 0.14 mg/ml *Rh*MOP130A incubated with 10mM di-galactosylated mannopentaose (G_2M_5) in 100 mM sodium citrate pH 6.0 at 37°C for 0-1 h. From top to bottom, the chromatograms in the figure are: control injection of 10 µM G_2M_5, then the reaction with G_2M_5 terminated after 24, 1, 0 h respectively, followed by an *Rh*MOP130A enzyme control.

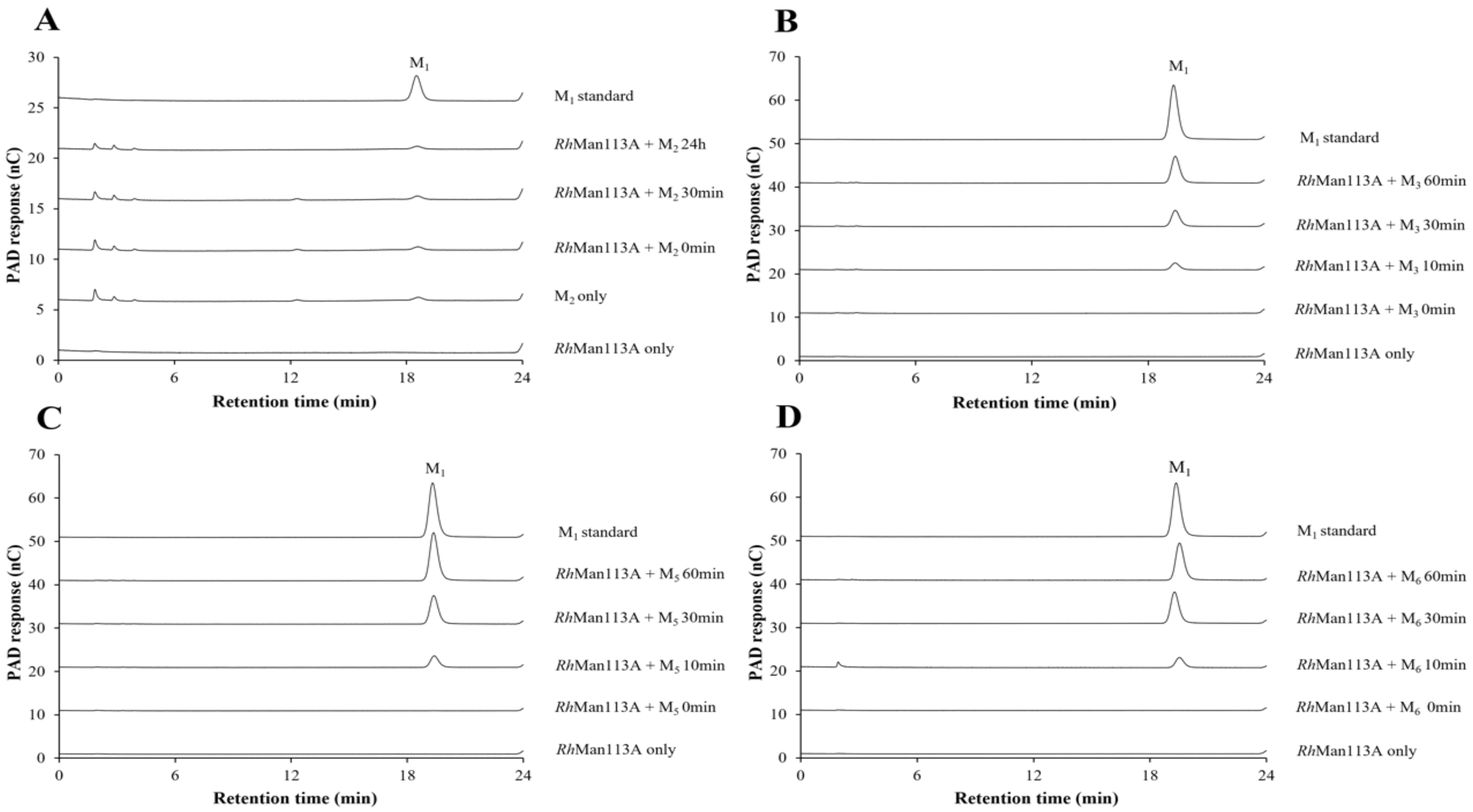

Figure S14. HPEAC-PAD (PA20 column) analysis of the mannose release when *Rh*Man113A (5 µg/ml) was incubated with 5 mM mannan-oligosaccharides. A: mannobiose (M_2), B: mannotriose (M_3), C: mannopentaose (M_5), and D: mannohexaose (M_6). The reactions were performed in triplicates at 30°C in 50 mM sodium phosphate buffer pH 6. Aliquots from the reaction mixtures were collected and the reaction was terminated after 0-, 10-, 30-, and 60-minutes. Reaction time-points for incubation with M2 was 0-, 30 minutes, and 24 hours.

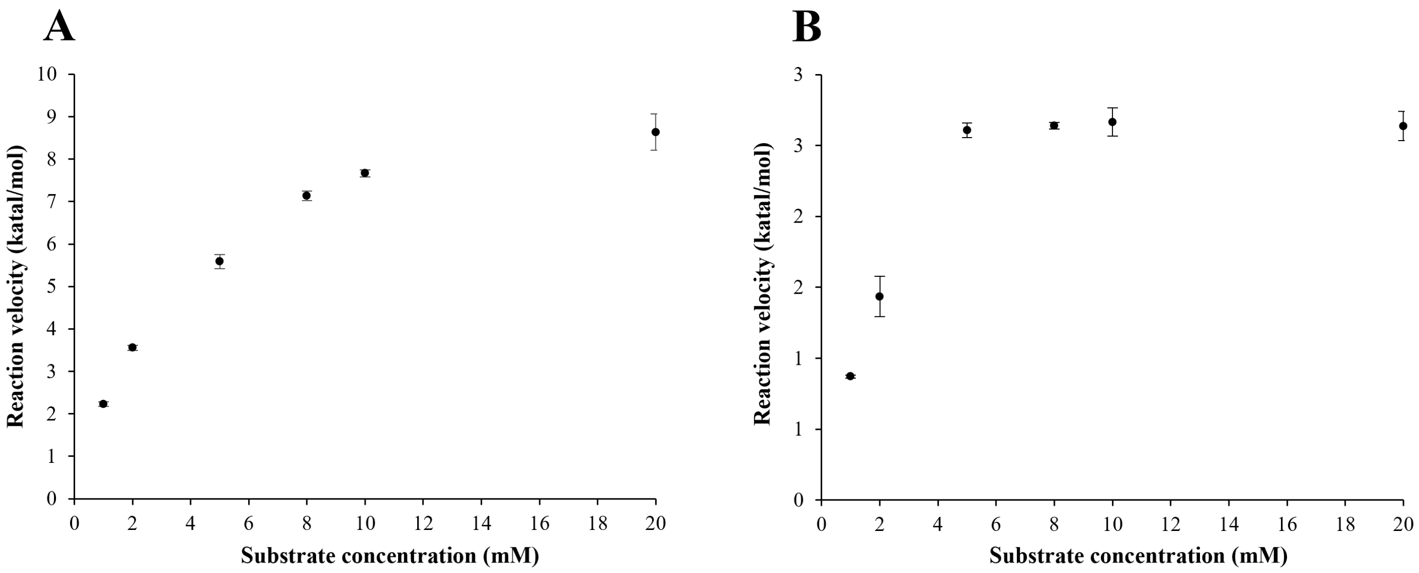

Figure S15. Michaelis-Menten kinetics of *Rh*Man113A. A: mannotetraose (M_4) and B: mannopentaose (M_5). The incubations were performed in triplicates at 30°C and pH 6 using 5 µg/ml enzyme. The reactions were terminated after 30 minutes.

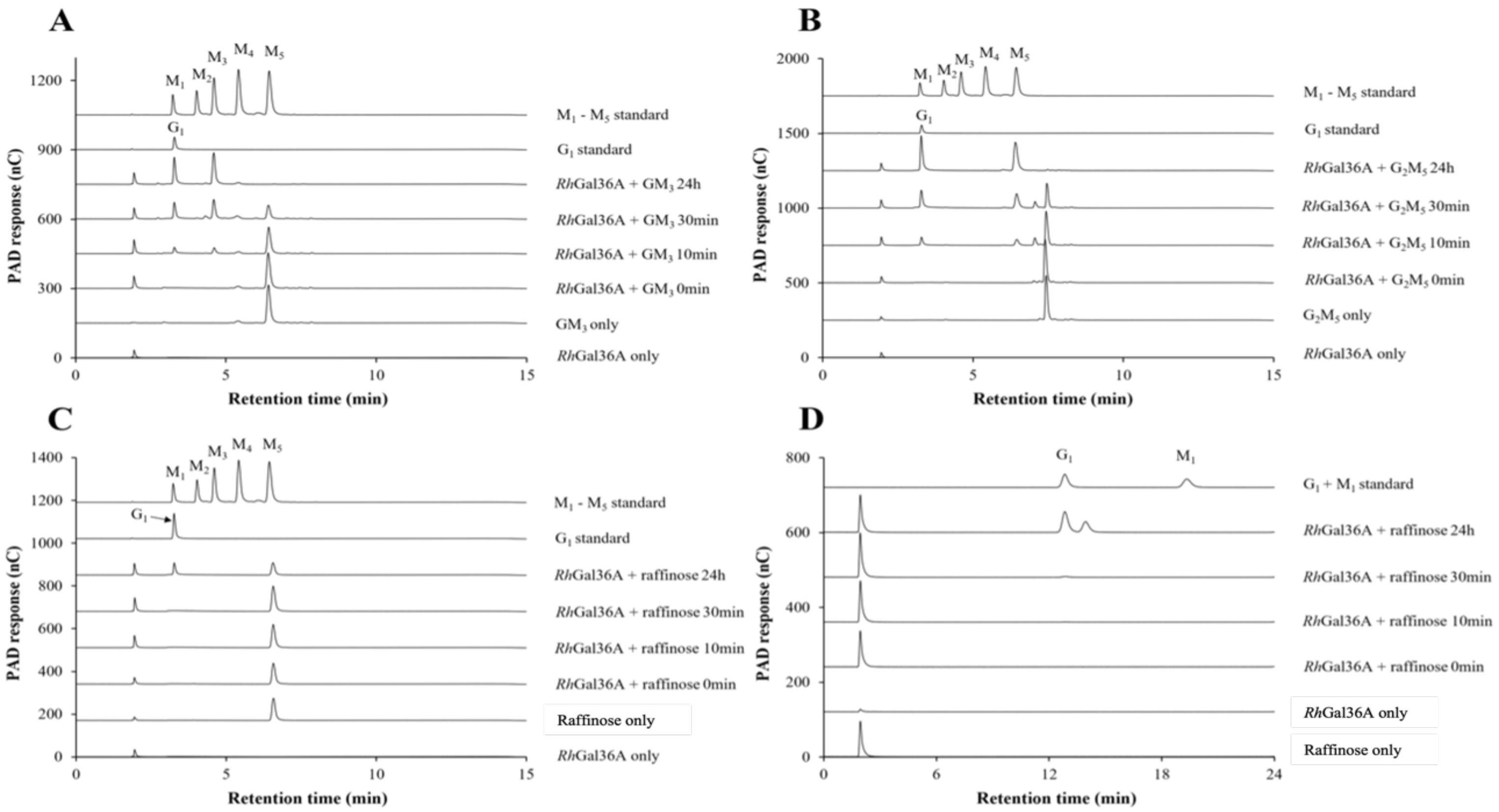

Figure S16. Galactose release by *Rh*Gal36A when incubated with galactose-containing substrates. All reactions were made in triplicates at 30°C in 50 mM sodium citrate buffer pH 5.5 using 5 µg/ml enzyme and 5 mM substrate. **A**: *Rh*Gal36A incubated with 6^1-α-D-Galactosyl-mannotriose (GM3), **B**: *Rh*Gal36A incubated with 6^3, 6^4-α-D-Galactosyl-mannopentaose (G_2M_5), **C-D**: *Rh*Gal36A incubated with raffinose. Reactions aliquots were collected and terminated after 0-, 10-, 30 minutes, and 24 hours. Reaction products were analysed using HPAEC-PAD with a PA200 column (**A, B, C**) and a PA20 column (**D**). G_1 indicates galactose (migrates as M1 with PA200 column). The G_1 formation was confirmed by PA20-analysis for all incubations.

Supplementary text

S1. Pre-inoculum preparation

For the cultivation on glucose, the inoculum was prepared from the stock cultures of *B. adolescentis* and *R. hominis* by first inoculating (2%, v/v) into 7.0 mL of MCB or mMCB and incubated at 37 °C for 24 h. Next, 0.5% (v/v) inoculum of *B. adolescentis* or *R. hominis* from previous cultivation was inoculated into 10 mL of MCB or mMCB, followed by anaerobic incubation at 37 °C for 12 h. Subsequently, a second passage into the MCB or mMCB medium was carried out under the same conditions. Later, the third subculture of 1.5 % or 2.0 % (v/v) of *B. adolescentis* or *R. hominis*, respectively, was inoculated in the same medium and was incubated at 37 °C for 3 h. Subsequently, the optical density (OD) at 600 nm was determined to be 0.238 and 0.207 for *B. adolescentis* and *R. hominis*, respectively. 2.0% (v/v) of this inoculum was then used as pre-culture for inoculating the growth medium (100.0 mL) for either *B. adolescentis* on MCB or *R. hominis* on mMCB for monocultivations. For cocultivation, 2% (v/v) of both *B. adolescentis* and *R. hominis* were used as pre-culture for inoculating 100 mL of MCB.

For cultivation on MOS/GMOS, the inoculum was prepared as mentioned above, except during the third subculture, wherein, *B. adolescentis* and *R. hominis* were incubated for 6 h at 37 °C. The OD at 600 nm was determined to be 0.201 & 0.234 for *B. adolescentis* & *R. hominis*, respectively. 2.0% (v/v) of this inoculum was then used as a pre-culture for inoculating the monocultures and cocultures as mentioned above.

S2. Gene copy number estimation

The gene copy number per mL was calculated using the following equation (1) (Whelan et al., 2003):

Equation 1:

$$\text{Gene copy number/mL} = \frac{6.02 \times 10^{23}\ (\text{copy/mol}) \times \text{DNA amount (g)}}{\text{Length of amplicon (bp)} \times \text{average mass of one bp (g/mol)}}$$

Where the 6.02 x 1023 is the number of molecules per mole; the length of amplicon for *rpo*B and *rec*A used a marker gene in *R. hominis* & *B. adolescentis* were 107 & 112 bp, respectively; the average mass of 1 bp was considered 660; the amount of DNA was determined as 10^(C_T-b/m), where 10 is the log quantity, C_T or cycle threshold value is obtained from qPCR run, b is the intercept from the standard curve and m is the slope from the standard curve.

Furthermore, the E value (PCR amplification efficiency) for each primer set was determined (target and reference genes) based on a qPCR assay with 10-fold serial dilution of amplification products (4.0 ng to 4.0 pg) and plotting the logarithm of the concentrations as a linear function of C_T values. E values were calculated as $10^{-1/\text{slope}}$ using the slope of each standard curve.

References for supplementary material

(The references are also given in main text)

La Rosa, S.L.; Leth, M.L.; Michalak, L.; Hansen, M.E.; Pudlo, N.A.; Glowacki, R.; Pereira, G.; Workman, C.T.; Arntzen, M.; Pope, P.B.; et al. The Human Gut Firmicute Roseburia Intestinalis Is a Primary Degrader of Dietary β-Mannans. *Nat. Commun.* **2019**, *10*, 1–14, doi:10.1038/s41467-019-08812-y.

Bhattacharya, A.; Wiemann, M.; Stålbrand, H. β-Mannanase BoMan26B from Bacteroides Ovatus Produces Mannan-Oligosaccharides with Prebiotic Potential from Galactomannan and Softwood β-Mannans. *Lwt* **2021**, *151*, 112215, doi:10.1016/j.lwt.2021.112215.

Ladevéze, S.; Tarquis, L.; Cecchini, D.A.; Bercovici, J.; André, I.; Topham, C.M.; Morel, S.; Laville, E.; Monsan, P.; Lombard, V.; et al. Role of Glycoside Phosphorylases in Mannose Foraging by Human Gut Bacteria. *J. Biol. Chem.* **2013**, *288*, 32370–32383,

doi:10.1074/jbc.M113.483628

Li, A.; Benkoulouche, M.; Ladeveze, S.; Durand, J.; Cioci, G.; Laville, E.; Potocki-Veronese, G. Discovery and Biotechnological Exploitation of Glycoside-Phosphorylases. *Int. J. Mol. Sci.* **2022**, *23*, doi:10.3390/ijms23063043.

Merceron, R.; Foucault, M.; Haser, R.; Mattes, R.; Watzlawick, H.; Gouet, P. The Molecular Mechanism of Thermostable α-Galactosidases AgaA and AgaB Explained by X-Ray Crystallography and Mutational Studies. *J. Biol. Chem.* **2012**, *287*, 39642–39652, doi:10.1074/jbc.M112.394114.

Ejby, M.; Guskov, A.; Pichler, M.J.; Zanten, G.C.; Schoof, E.; Saburi, W.; Slotboom, D.J.; Abou Hachem, M. Two Binding Proteins of the ABC Transporter That Confers Growth of Bifidobacterium Animalis Subsp. Lactis ATCC27673 on β-Mannan Possess Distinct Manno-Oligosaccharide-Binding Profiles. *Mol. Microbiol.* **2019**, *112*, 114–130, doi:10.1111/mmi.14257.

Whelan, J.A.; Russell, N.B.; Whelan, M.A. A Method for the Absolute Quantification of cDNA Using Real-Time PCR. *J. Immunol. Methods* **2003**, *278*, 261–269, doi:10.1016/S0022-1759(03)00223-0.

.
